# Backbone conformation affects duplex initiation and duplex propagation in hybridisation of synthetic H-bonding oligomers†
†Electronic supplementary information (ESI) available. CCDC 1835177. For ESI and crystallographic data in CIF or other electronic format see DOI: 10.1039/c8ob00819a


**DOI:** 10.1039/c8ob00819a

**Published:** 2018-05-23

**Authors:** Giulia Iadevaia, Diego Núñez-Villanueva, Alexander E. Stross, Christopher A. Hunter

**Affiliations:** a Department of Chemistry , University of Cambridge , Lensfield Road , Cambridge CB21EW , UK . Email: herchelsmith.orgchem@ch.cam.ac.uk

## Abstract

Synthetic oligomers equipped with complementary H-bond donor and acceptor side chains form multiply H-bonded duplexes in organic solvents. Comparison of the duplex forming properties of four families of oligomers with different backbones shows that formation of an extended duplex with three or four inter-strand H-bonds is more challenging than formation of complexes that make only two H-bonds. The stabilities of 1 : 1 complexes formed between length complementary homo-oligomers equipped with either phosphine oxide or phenol recognition modules were measured in toluene. When the backbone is very flexible (pentane-1,5-diyl thioether), the stability increases uniformly by an order of magnitude for each additional base-pair added to the duplex: the effective molarities for formation of the first intramolecular H-bond (duplex initiation) and subsequent intramolecular H-bonds (duplex propagation) are similar. This flexible system is compared with three more rigid backbones that are isomeric combinations of an aromatic ring and methylene groups. One of the rigid systems behaves in exactly the same way as the flexible backbone, but the other two do not. For these systems, the effective molarity for formation of the first intramolecular H-bond is the same as that found for the other two backbones, but additional H-bonds are not formed between the longer oligomers. The effective molarities are too low for duplex propagation in these systems, because the oligomer backbones cannot adopt conformations compatible with formation of an extended duplex.

## Abstract 2

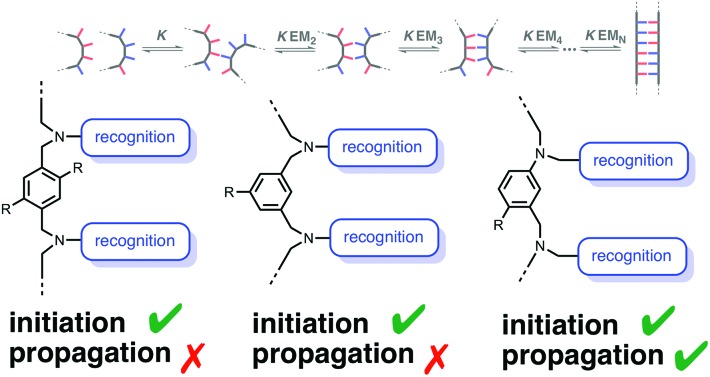
Forming the first intramolecular H-bond is straightforward, but forming subsequent intramolecular interactions is difficult, because the backbone imposes more severe constraints.

## Introduction

Nucleic acids store and express genetic information *via* sequence selective duplex formation and template directed synthesis.^1^ Variation in the chemical structure of the backbone,^2^ the base pairing system,^3^ the phosphate diester linker^4^ and the sugar^5^ have been explored, and the ability to form a stable duplex is maintained despite these modifications. This observation suggests that oligomers with very different chemical structures may also be able to form duplexes in a sequence selective manner. A number of synthetic oligomers have been reported that form duplexes through different types of non-covalent interaction, including metal–ligand coordination,^6^ aromatic stacking,^7^ salt bridges^8^ and H-bonding.^9^

**Fig. 1:**
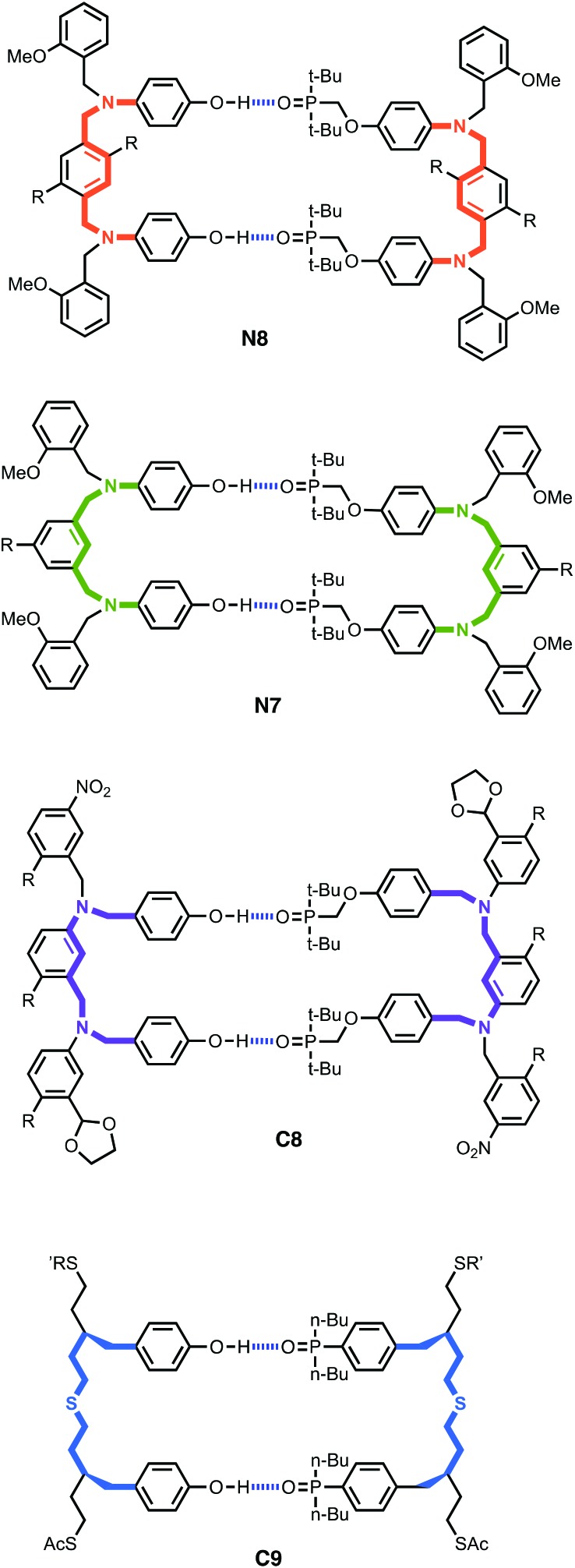
H-bonded duplexes. Different backbones lead to equally stable 2-mer duplexes. The C8 backbone is highlighted in purple, the N8 backbone in orange, the N7 backbone in green, and the C9 in blue. R is 2-ethylhexyloxy and R′ is *n*-hexyl.

Fig. 1 shows the structures of four different H-bonded duplexes formed by oligomers equipped with phenol and phosphine oxide recognition units.^10^ All combinations of 2-mers that have the N7, N8 and C8 backbones shown in Fig. 1 form duplexes with similar stabilities.10b,d In fact, the effective molarities for formation of the intramolecular H-bonds that lead to duplex formation in all of the systems shown in Fig. 1 are remarkably similar (7–20 mM).^11^ These results suggest that conformational properties of the backbone are not critical for duplex formation, so oligomers with the highly flexible C9 backbone form duplexes that have similar stability to the duplexes formed by oligomers with the more rigid C8 backbone. Here we describe a new family of oligomers where the conformational properties of the backbone compromise duplex formation between longer oligomers.

**Fig. 2:**
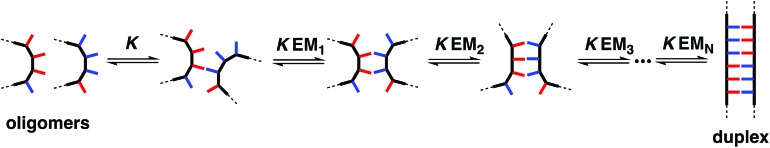
Stepwise assembly of a duplex from two complementary oligomers. *K* is the association constant for formation of an intermolecular interaction between two complementary H-bonding sites, and EM_*N*_ are the stepwise effective molarities for formation of the intramolecular interactions that lead to zipping up of the duplex.

The stepwise equilibria for the assembly of two complementary oligomers into a duplex are shown in Fig. 2.^12^ After the first intermolecular interaction, that has an association constant of *K*, the subsequent intramolecular interactions have an equilibrium constant of *K*EM_*N*_, where EM_*N*_ is the stepwise effective molarity, and *N* is the number of intramolecular interactions formed. Fully bound duplexes are formed, if the values of *K*EM_*N*_ are all significantly greater than one. By comparing the stabilities of duplexes of increasing chain length, it is possible to experimentally determine values of EM_*N*_. In this paper, we show that the effective molarity for the first intramolecular interaction responsible for duplex initiation (EM_1_) can be very different from the values of EM for the subsequent intramolecular interactions that lead to duplex propagation.

The synthesis of the homo-oligomers up to four recognition units in length was previously reported for the C8 and C9 backbones.10*a* Here we describe the synthesis of the corresponding homo-oligomers with the N7 and N8 backbones. Measurement of the stabilities of the duplexes formed by all complementary pairs of oligomers allows characterisation of the stepwise effective molarities for duplex initiation and propagation.

## Results and discussion

### Synthesis

**Scheme 1:**
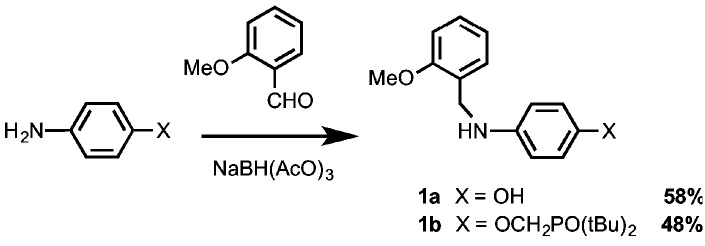


A convergent approach was taken to obtain the oligomers from a set of common building blocks. Secondary anilines **1a** and **1b**, which were used as chain end caps, were prepared *via* reductive amination of the corresponding primary anilines (Scheme 1). The H-bond donor primary aniline, 4-aminophenol, is commercially available, and synthesis of the H-bond acceptor primary aniline bearing a phosphine oxide was reported previously.10*b*

**Scheme 2:**
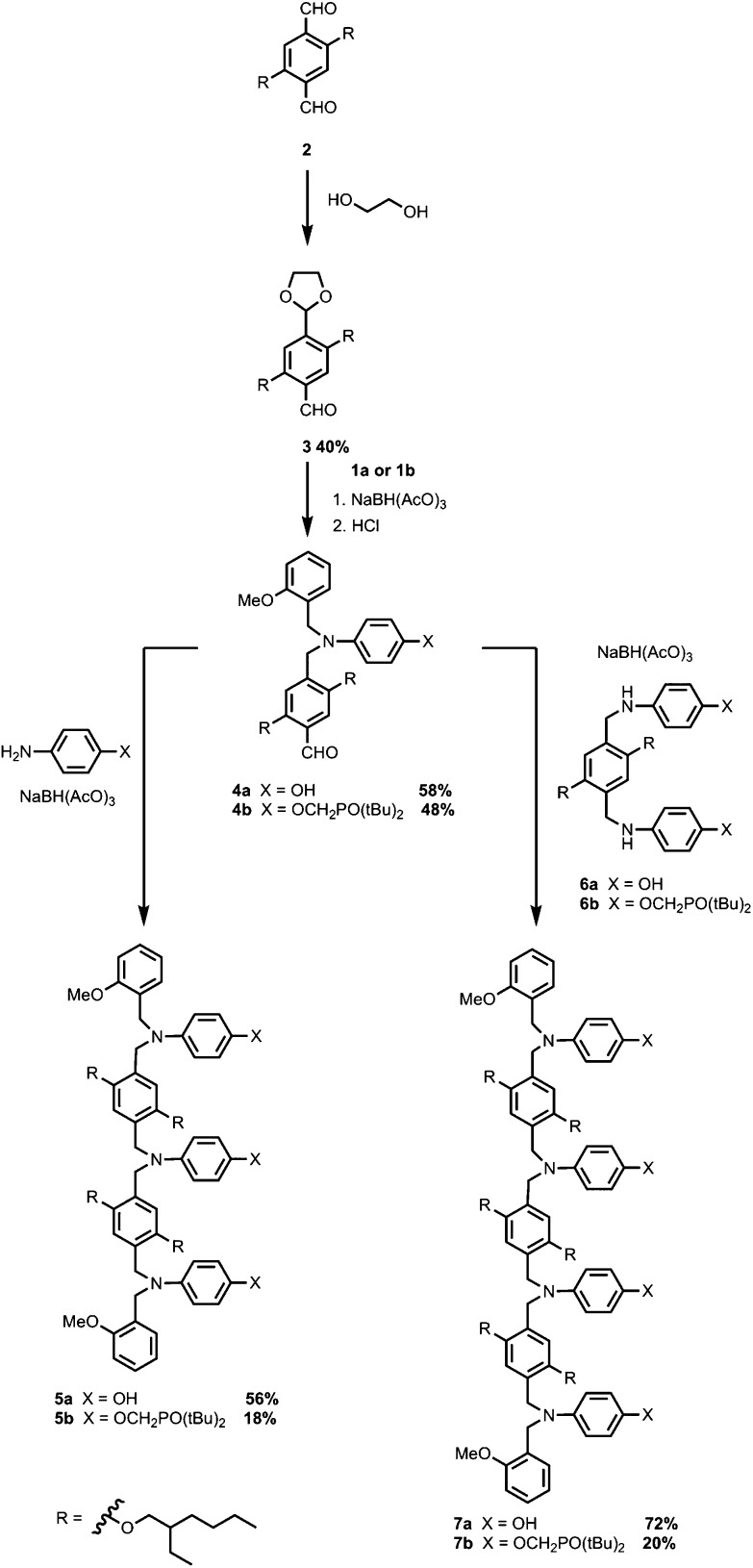


Scheme 2 shows the synthetic route to the 3-mers and 4-mers for the N8 backbone. Monoprotected aldehyde **3** was prepared by refluxing **2** with one equivalent of ethylene glycol in toluene (Scheme 2).^13^ Aldehydes **4a** and **4b** were synthesized by reductive amination of **3** with **1a** or **1b**, followed by acetal deprotection (Scheme 2). 3-mers **5a** and **5b** were obtained by reductive amination of **4a** or **4b** with the corresponding primary aniline. The bisanilines **6a** and **6b** were prepared from **2** as described previously.10*b* Reductive amination of aldehyde **4a** with aniline **6a** gave the all donor 4-mer **7a**, and reductive amination of aldehyde **4b** with aniline **6b** gave the all acceptor 4-mer **7b**.

**Scheme 3:**
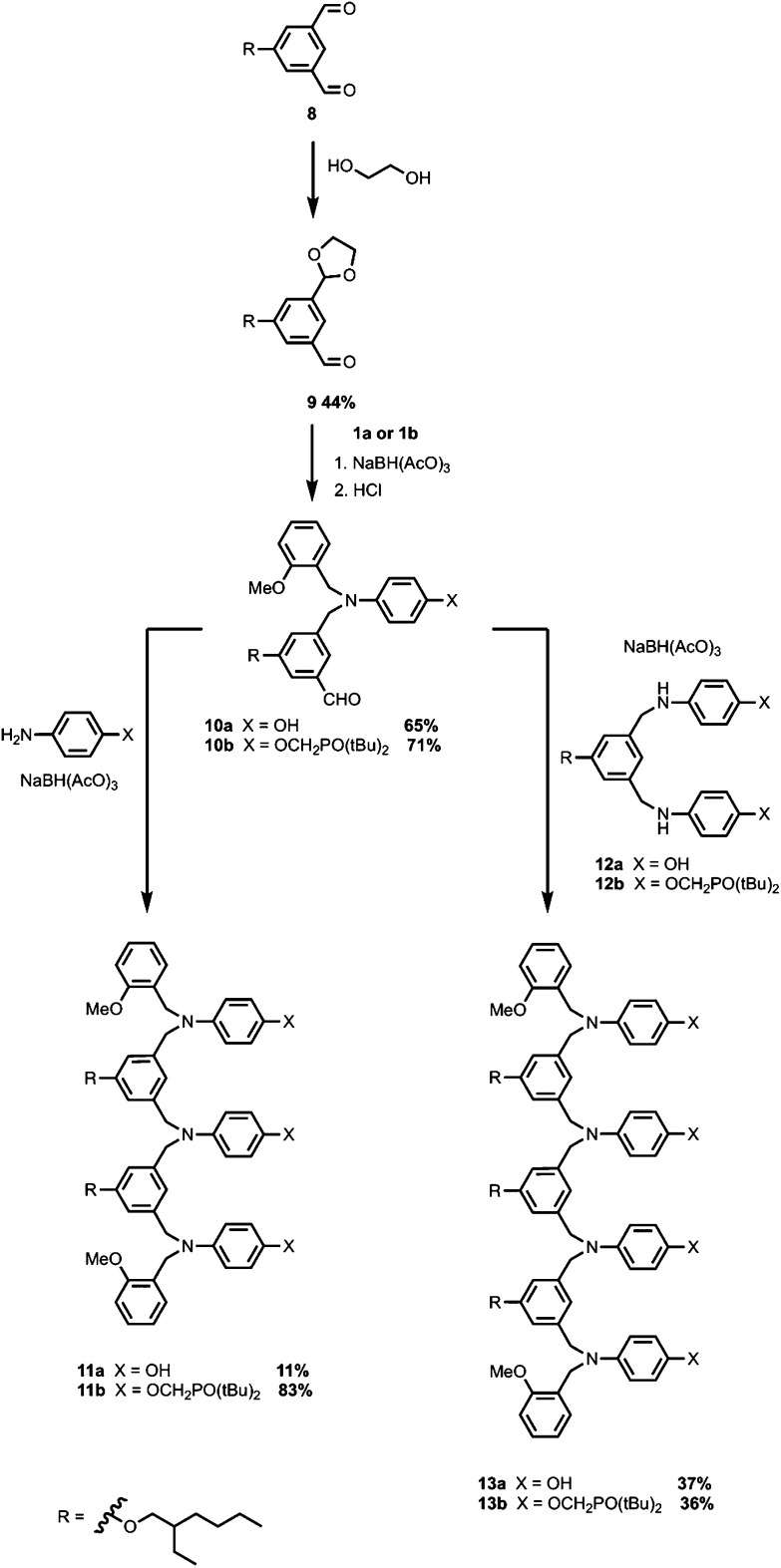


A similar synthetic route was used for the synthesis of the N7 backbone oligomers (Scheme 3). Reductive amination of monoprotected aldehyde **9** with **1a** or **1b** gave aldehydes **10a** and **10b** respectively. The 3-mers **11a** and **11b** were obtained by reductive amination of **10a** or **10b** with the corresponding primary aniline. The bisanilines **12a** and **12b** were prepared from **8** as described previously.10*b* Reductive amination of aldehyde **10a** with **12a** the all donor 4-mer **13a**, and reductive amination of aldehyde **10b** with **12b** gave the all acceptor 4-mers **13b** (Scheme 3).

### NMR binding studies

Association constants between complementary H-bond donor (D) and H-bond acceptor (A) oligomers were determined *via*^31^P NMR titrations in toluene-*d*_8_. In all cases, the H-bond acceptor phosphine oxide oligomer was used as the host, and the data fit well to 1 : 1 binding isotherms. The results are summarized in Table 1, together with data for the corresponding 1-mer and 2-mer complexes previously reported.10a,b,d Table 1 also reports the complexation-induced changes in chemical shift of the ^31^P signals. The large increases in ^31^P chemical shift are characteristic of H-bond formation.^14^ The association constants for the 3-mer complexes (AAA·DDD) and for the 4-mer complexes (AAAA·DDDD) are of the same order of magnitude as the association constant for the corresponding 2-mer complexes (AA·DD) (Table 1).

**Table 1:** Association constants *K*_N_ and limiting complexation-induced changes in ^31^P NMR chemical shift for formation of 1 : 1 complexes in toluene-*d*_8_ at 298 K

Backbone	Complex	log *K*_*N*_/M^–1^	Δ*δ*^31^P/ppm
—	A·D	2.4 ± 0.1	5.0
N8	AA·DD	3.4 ± 0.1	3.9
N8	AAA·DDD	3.5 ± 0.2	3.8, 4.7
N8	AAAA·DDDD	3.8 ± 0.1	4.3, 4.3
N7	AA·DD	3.1 ± 0.2	6.9
N7	AAA·DDD	3.4 ± 0.1	7.6, 7.6
N7	AAAA·DDDD	3.3 ± 0.1	8.5, 8.6

**Fig. 3:**
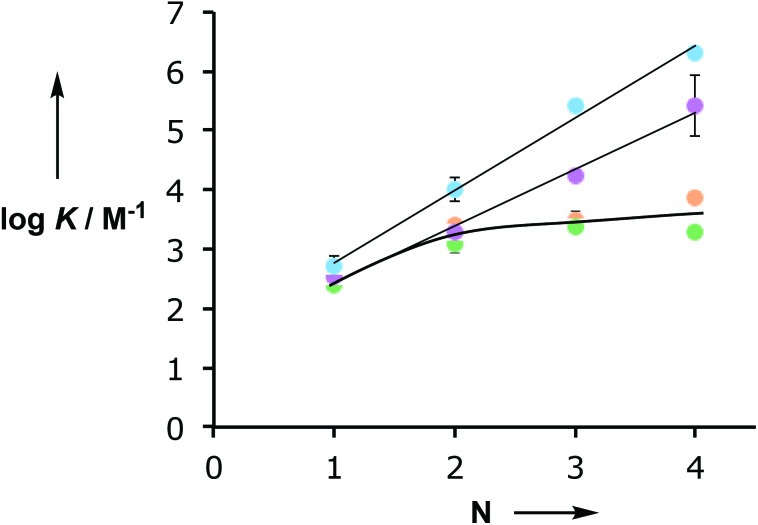
Relationship between the association constant for duplex formation (log *K*_*N*_) and the number of recognition units in an oligomer (*N*) for the C8 (purple),10*a* C9 (blue),10*d* N8 (orange) and N7 (green) backbones.

Fig. 3 shows the relationship between the association constant for duplex formation (log *K*_*N*_) and the number of potential H-bonding interactions (*N*). The association constants for the C8 and C9 backbones increase uniformly by an order of magnitude for each H-bonding site added to the chain. For the N8 and N7 backbones, the 2-mer duplex is an order of magnitude more stable than the singly H-bonded 1-mer complex, but there is no significant increase in association constant for the 3-mer or 4-mer complexes. These results suggest that in the longer N8 and N7 oligomers only two H-bonds are formed and that the fully assembled duplexes are not stable. One would expect a corresponding difference in the magnitudes of the complexation-induced changes in ^31^P NMR chemical shift for the systems that are not fully H-bonded, but the changes in chemical shift are actually larger for the longer N8 and N7 oligomers. This discrepancy must reflect some difference in three-dimensional structure for these complexes or the formation of higher order aggregates that do not significantly affect the shape of the binding isotherm.

### Conformational analysis

**Fig. 4:**
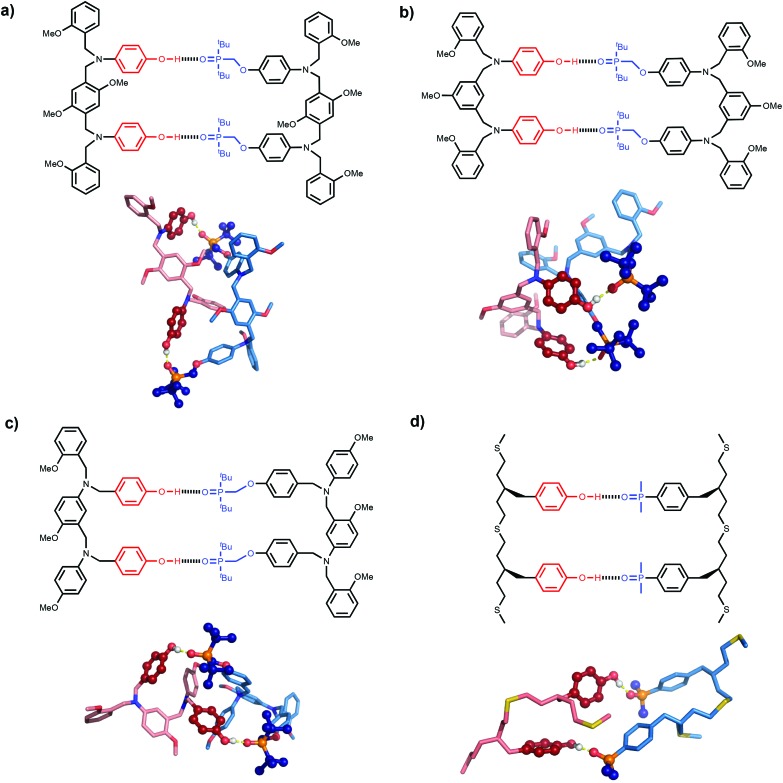
Lowest energy conformations of the 2-mer AA·DD duplexes from conformational searches (MMFFs force-field and CHCl_3_ solvation implemented in Macromodel)^15^ for the (a) N8, (b) N7, (c) C8 and (d) C9 backbones. The H-bond donor oligomer is shown in red and the H-bond acceptor oligomer in blue. The recognition modules are shown as atomic spheres, H-bonds are shown in yellow, and hydrogen atoms on carbon are not shown for clarity. The chemical structures highlight the key interactions made in the calculated structures.

**Fig. 5:**
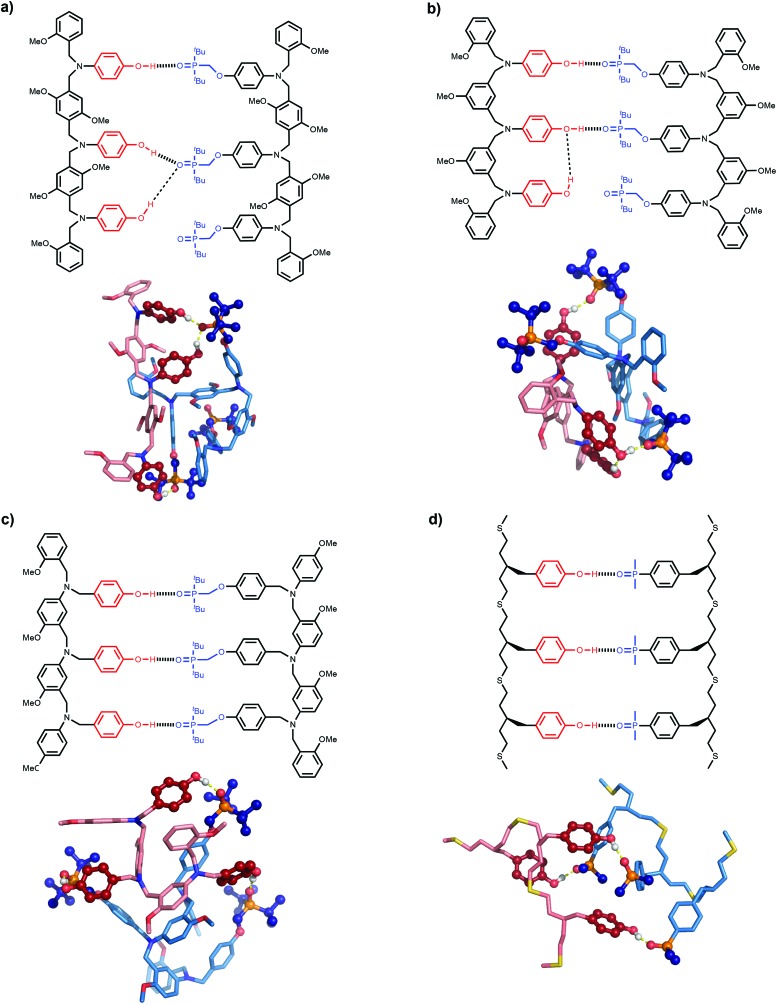
Lowest energy conformations of the 3-mer AAA·DDD duplexes from conformational searches (MMFFs force-field and CHCl_3_ solvation implemented in Macromodel)^15^ for the (a) N8, (b) N7, (c) C8 and (d) C9 backbones. The H-bond donor oligomer is shown in red and the H-bond acceptor oligomer in blue. The recognition modules are shown as atomic spheres, H-bonds are shown in yellow, and hydrogen atoms on carbons are not shown for clarity. The chemical structures highlight the key interactions made in the calculated structures.

The difference in behaviour between the four different systems is likely to be due to geometrical incompatibility of the N8 and N7 backbones with the fully bound duplex. The geometrical properties of the backbones were therefore investigated using molecular mechanic calculations.^15^ By using a distance constraint to make sure that at least one intermolecular H-bond is formed in the duplex, it is possible to restrict the conformational search space to a tractable size. Fig. 4 shows the lowest energy structures obtained from conformational searches for 2-mer AA·DD duplexes with the four different backbones. In all cases, the fully assembled duplex with two A·D H-bonds was found. Fig. 5 shows the lowest energy structures obtained from conformational searches for 3-mer AAA·DDD duplexes with the four different backbones. In this case, the fully assembled duplex with three A·D H-bonds was found for the C8 and C9 backbones, but not for the N8 and N7 backbones. In the N8 and N7 backbone structures, only two of the recognition units are properly paired. Moreover, the fully H-bonded duplex with all three A·D H-bonds was not located at all in the conformational search for either of these systems, which suggests that this structure is considerably higher in energy than the global energy minimum.

The computational results are consistent with the experimental observations. All of the backbones are compatible with formation of the first and second H-bond of the duplex, but only the C8 and C9 backbones can make the third H-bond. For the two complexes that do not form the fully assembled duplex, the unsatisfied H-bond donor makes an additional interaction with an alternative site. For the N8 backbone, the unpaired terminal phenol interacts with the central phosphine oxide that is already engaged in a H-bond with the complementary central phenol. For the N7 backbone, the unpaired terminal phenol interacts with the central phenol that is already H-bonded to the complementary central phosphine oxide. The experimental association constants indicate that if these additional interactions are present, then they do not contribute significantly to the stabilities of the complexes.

**Fig. 6:**
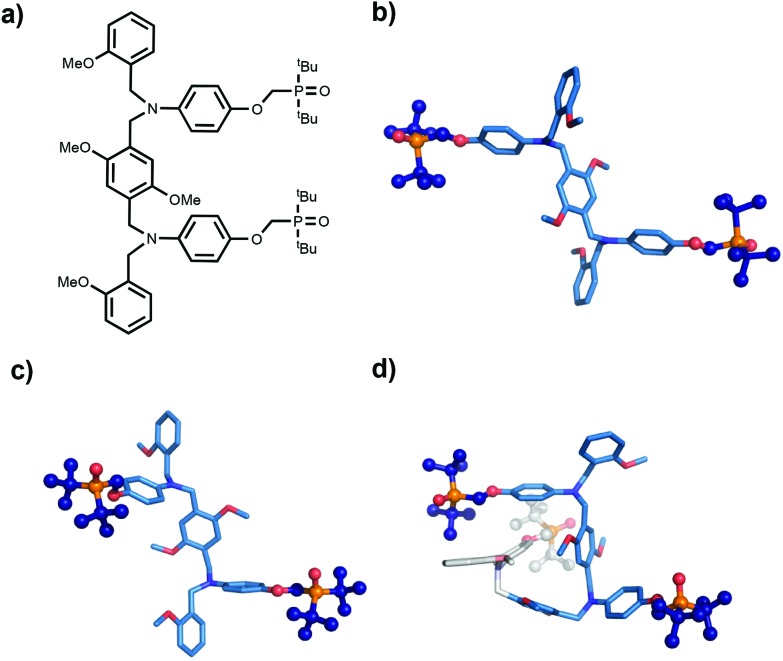
Conformation of the N8 backbone. (a) Chemical structure of the AA 2-mer; (b) X-ray structure of the AA 2-mer; (c) conformation of the AA 2-mer in the lowest energy molecular mechanics structure of the AA·DD duplex; (d) conformation of the AAA 3-mer in the lowest energy molecular mechanics structure of the AAA·DDD duplex. The first two recognition modules that are H-bonded in the duplex are highlighted in blue for comparison with (b) and (c), and the third recognition module that is not involved in H-bonding in the duplex is shown in grey.

The molecular mechanics calculations should provide some insight into the reasons for the failure of the N8 and N7 backbones, but the structures in Fig. 5 are rather complicated. Further experimental information was obtained from X-ray crystallography. Single crystals suitable for X-ray analysis were obtained for the phosphine oxide 2-mer with the N8 backbone. Fig. 6 compares the backbone conformation in the solid state with the conformations of the 2-mer and 3-mer backbones from the energy minimum molecular mechanics structures of the duplexes shown in Fig. 4 and 5.

The X-ray and the molecular mechanics structures of the 2-mer are very similar: the two recognition modules are oriented in an *anti* arrangement, and this motif is repeated in the 3-mer molecular mechanics structure. The X-ray structure indicates that the molecular mechanics calculations provide a reasonable description of the conformational properties of these molecules. Moreover, the results suggest a reason why the N8 backbone does not make an extended multiply H-bonded duplex. Duplex formation requires a *syn* arrangement of the recognition modules with all of the interaction sites pointing in the same direction, but the structures in Fig. 6 imply that the N8 backbone prefers to adopt an alternating *anti* arrangement of recognition modules along the chain.

## Conclusions

**Fig. 7:**
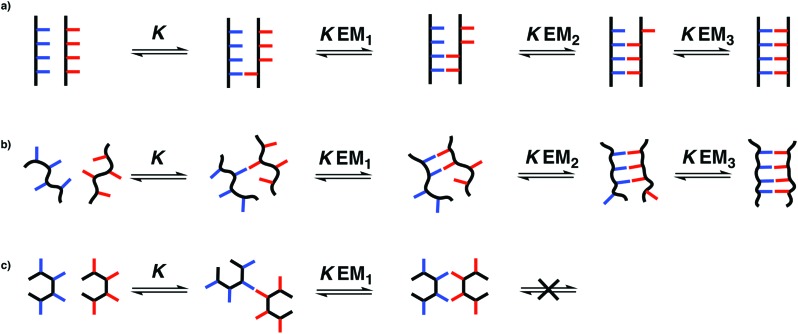
Relationship between the conformational properties of the backbone and stepwise effective molarities in the formation of non-covalent duplexes. (a) A rigid backbone will form a duplex if it is geometrically compatible. (b) A very flexible backbone will always be able find a conformational state that is geometrically compatible with the duplex. (c) If a rigid backbone is not geometrically compatible with the duplex, the first effective molarity for duplex initiation (EM_1_) and the subsequent effective molarities for duplex propagation (EM_2_) will be different, so only the 2-mer duplex will assemble.

The conformational flexibility of the backbone plays an important role in determining the ability of complementary H-bonding oligomers to form a fully bound duplex. For a relatively rigid backbone that has conformational properties compatible with duplex formation (*e.g.* C8), the stability of the duplex increases uniformly with the number of recognition sites present in the oligomers. For the C8 systems, the effective molarity for formation of the first intramolecular H-bond, *i.e.* duplex initiation (EM_1_), and the effective molarities for subsequent intramolecular interactions that lead to zipping up of the duplex, *i.e.* duplex propagation (EM_2_, EM_3_*etc.*), have similar values (Fig. 7a). The same is true for highly flexible backbones (*e.g.* C9) that will always be able to find conformations compatible with a fully bound duplex (Fig. 7b).

The other two backbones described in this paper (N8 and N7) have quite different properties. These oligomers have relatively rigid backbones that appear to be incompatible with a fully assembled duplex. The effective molarities for duplex initiation (EM_1_) for both of these systems are similar to the values found for the C8 and C9 backbones, but the effective molarities for duplex propagation (EM_2_) are much lower. The reason is that formation of a doubly H-bonded complex imposes little conformational constraint on the backbone, whereas formation of a triply H-bonded complex can only be achieved within a much more restricted conformational window. As a result, the N7 and N8 backbones are not compatible with the formation of an extended multiply H-bonded duplex, even though the 2-mers behave in exactly the same way as the C8 and C9 2-mers.

The results described here, together with our previous observations on the folding of AD 2-mers, indicate that the choice of backbone is critical to the successful development of synthetic information molecules designed to form H-bonded duplexes between complementary recognition sites. If the backbone is very flexible (C9), duplex formation will always be favourable for homo-sequence oligomers, but intramolecular interactions will complete with duplex formation in mixed sequence oligomers. The solution is to use a more rigid backbone, but as the results here show, this approach carries the risk that the conformational states accessible to the backbone may not be compatible with duplex formation. We have studied three isomeric backbones (C8, N7 and N8) that are a semi-flexible combination of an aromatic ring and two methylene groups. One of these systems forms stable duplexes and the other two do not. Conformational analysis using molecular modelling is consistent with the experimental properties of these systems and should provide a useful tool for future backbone design.

## Conflicts of interest

There are no conflicts to declare.

## Supplementary Material

Supplementary information

Crystal structure data
