# Supplementary material for: Backbone conformation affects duplex initiation and duplex propagation in hybridisation of synthetic H-bonding oligomers
Source: Org Biomol Chem. 2018 May 23;16(22):4183–90. doi: 10.1039/c8ob00819a (PMC5989393; doi:10.1039/c8ob00819a)
Supplement: Supplementary file 1 [file OB-016-C8OB00819A-s001.pdf]

Electronic Supplementary Material (ESI) for Organic & Biomolecular Chemistry.
This journal is © The Royal Society of Chemistry 2018

Backbone conformation affects duplex initiation and duplex propagation in hybridisation of synthetic H-bonding oligomers

Giulia Iadevaia, Diego Núñez-Villanueva, Alexander E. Stross, Christopher A. Hunter*

Department of Chemistry, University of Cambridge, Lensfield Road, Cambridge CB2 1EW, UK.

Supporting information

Synthesis S2
Binding studies S50
Molecular mechanic calculations S52
X-ray structure of the AA 2-mer of backbone N8 .... S53
References S54

Synthesis

Synthesis of 1a

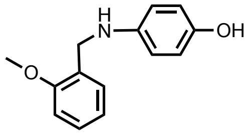

A mixture of *para*-aminophenol (2.10 g, 19 mmol), 2-methoxybenzaldehyde (2.10 ml, 17 mmol) and $NaBH(AcO)_3$ (5.40 g, 25 mmol) in DCM (120 ml) was stirred under nitrogen at room temperature for 1 h. The solution was then washed with saturated aqueous $NaHCO_3$ (1 x 100 ml), water (1 x 100 ml) and brine (1 x 100 ml), dried with $MgSO_4$, and the solvent was removed under reduced pressure. The crude product was then purified by column chromatography on silica eluting with hexane/EtOAc (70:30). The product was isolated as a brown solid (2.25 g, 58%).

^{1}H NMR (400 MHz, CD_3CN): δ 7.30-7.23 (m, 2H), 6.99-6.88 (m, 2H), 6.63-6.51 (m, 4H), 6.30 (s, 1H), 4.43 (s, 1H), 4.23 (s, 2H), 3.87 (s, 3H);

^{13}C NMR (100.6 MHz, $CDCl_3$): δ 157.5, 149.0, 141.4, 129.6, 128.6, 127.1, 120.6, 116.4, 116.1, 110.4, 55.4, 45.8;

MS (ES+): m/z (%) = 230.1184 (100) [$M+H^+$];

HRMS (ES+): calcd for $C_{14}H_{16}NO_2$ 230.1181, found 230.1184;

FT-IR (thin film): ν_{max} /cm^{-1} 3328, 3028, 2938, 2836, 1588, 1601.

m.p.: 116-118 °C.

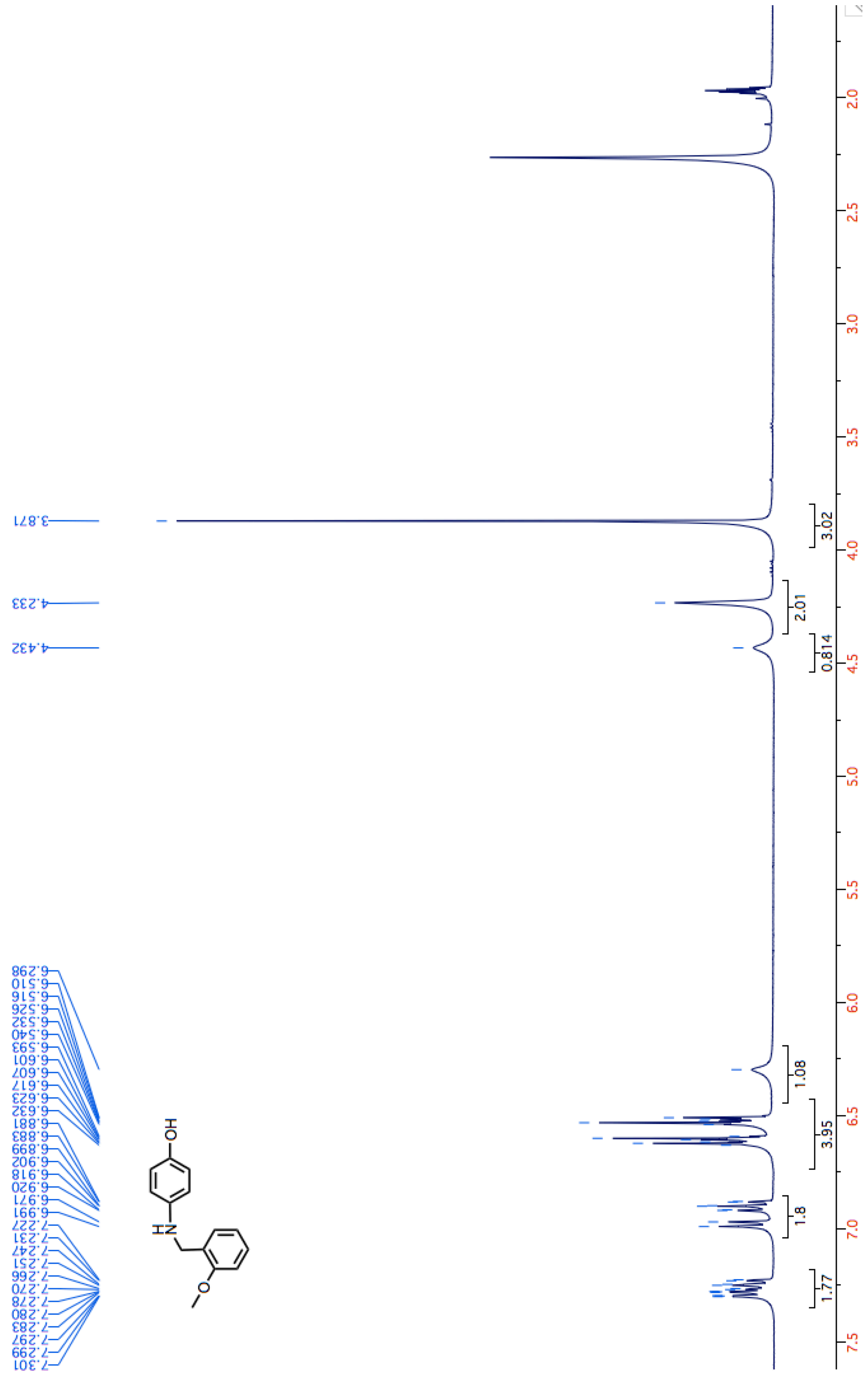
3.871
4.233
4.432
6.298
6.510
6.516
6.526
6.532
6.540
6.593
6.601
6.607
6.617
6.623
6.632
6.881
6.883
6.899
6.902
6.918
6.920
6.971
6.991
7.227
7.231
7.247
7.251
7.266
7.270
7.278
7.280
7.283
7.297
7.299
7.301
OH
HN
O
3.02
2.01
0.814
1.08
3.95
1.8
1.77
2.0
2.5
3.0
3.5
4.0
4.5
5.0
5.5
6.0
6.5
7.0
7.5

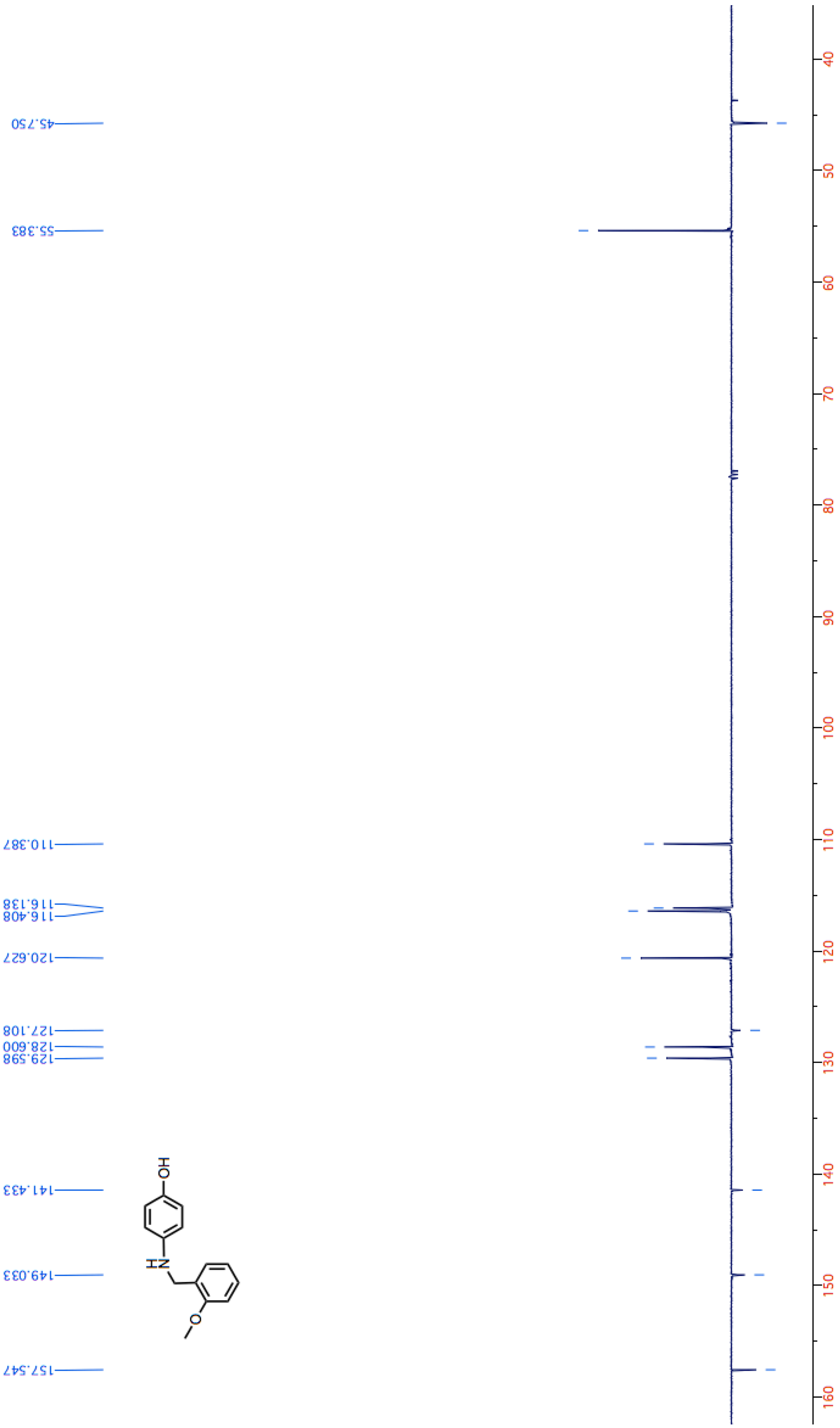
45.750
55.383
110.387
116.138
116.408
120.627
127.108
128.600
129.598
141.433
149.033
157.547
HO
NH
O
40
50
60
70
80
90
100
110
120
130
140
150
160

Synthesis of 1b

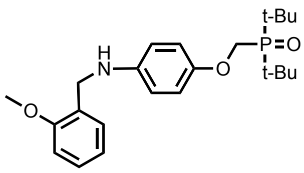

A mixture of di-*tert*-butyl((4-((2-methoxybenzyl)amino)phenoxy)methyl)phosphine oxide (0.17 g, 0.6 mmol), 2-methoxybenzaldehyde (0.06 ml, 0.5 mmol) and $NaBH(AcO)_3$ (0.16 g , 0.75 mmol) in DCE (10 ml) was stirred under nitrogen at room temperature for 1 h. The solution was then washed with saturated aqueous $NaHCO_3$ (1 x 10 ml), water (1 x 10 ml) and brine (1 x 10 ml), dried with $MgSO_4$, and the solvent was removed under reduced pressure. The crude product was then purified by column chromatography on silica eluting with DCM/EtOH (95:5). The product was isolated as a brown solid (0.105 g, 48%).

^{1}H NMR (400 MHz, CD_3CN): δ 7.27-7.20 (m, 2H), 6.96-6.85 (m, 2H), 6.81-6.79 (m, 2H), 6.61-6.58 (m, 2H), 4.27 (d, J = 6, 2H), 4.24 (s, 2H), 3.84 (s, 3H);
^{31}P NMR (162.0 MHz, CD_3CN): δ 55.6;
^{13}C NMR (100.6 MHz, CD_3CN): δ 157.6, 150.9, 150.9, 143.3, 128.6, 128.2, 127.5, 120.2, 115.3, 114.1, 110.5, 63.6, 62.9, 55.1, 43.1, 35.2, 34.6, 25.8;
MS (ES+): m/z (%) = 404.2 (100) [M+H$^+$];
HRMS (ES+): calcd for $C_{23}H_{35}NO_3P$ 404.2355, found 404.2339;
FT-IR (thin film): ν_{max} /cm^{-1} 3306, 2953, 1510.
m.p.: 129-130 °C.

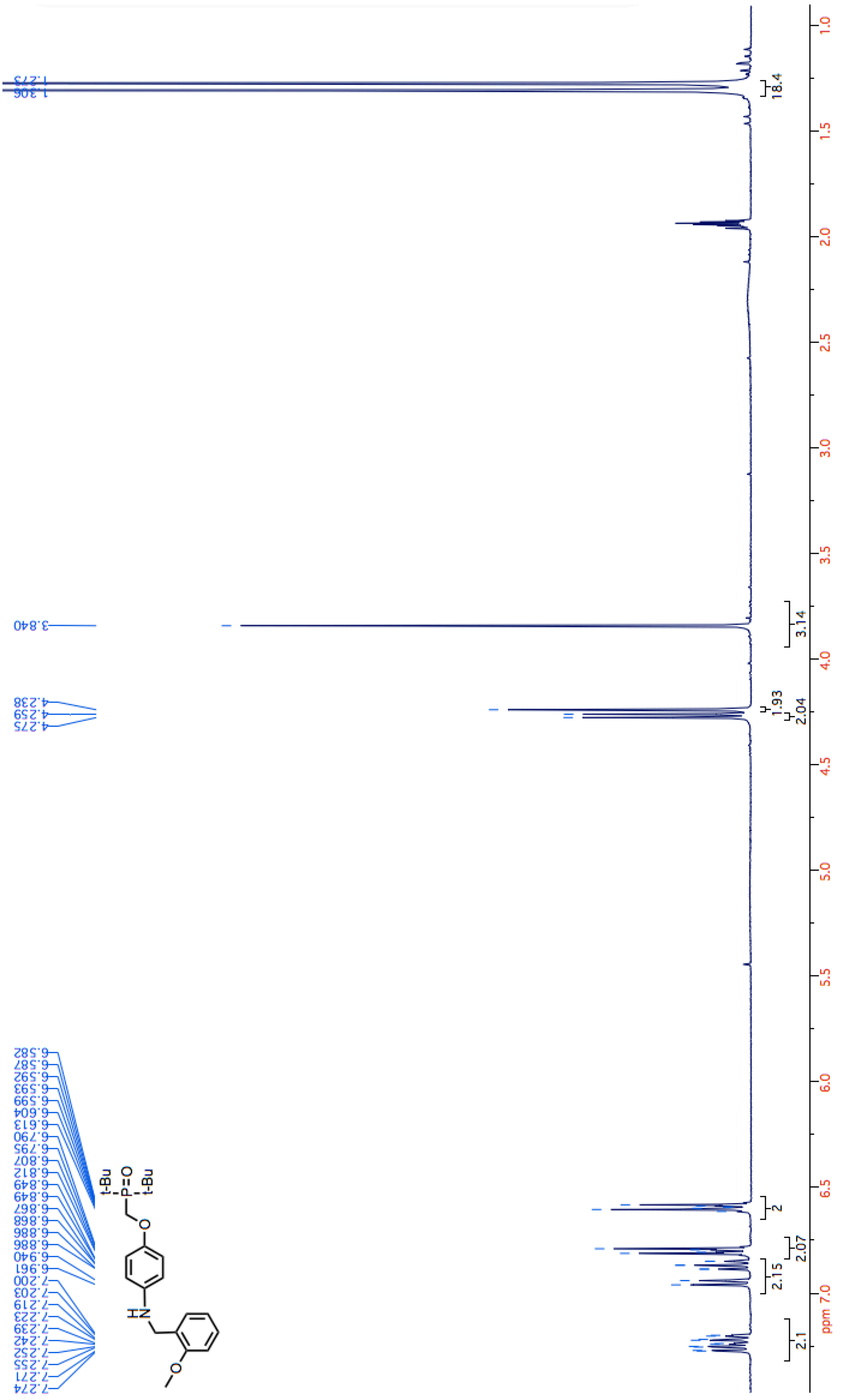

1.273
1.306
3.840
4.238
4.259
4.275
18.4
3.14
1.93
2.04
2
2.07
2.15
2.1
t-Bu
P=O
HN
ppm
1.0
1.5
2.0
2.5
3.0
3.5
4.0
4.5
5.0
5.5
6.0
6.5
7.0

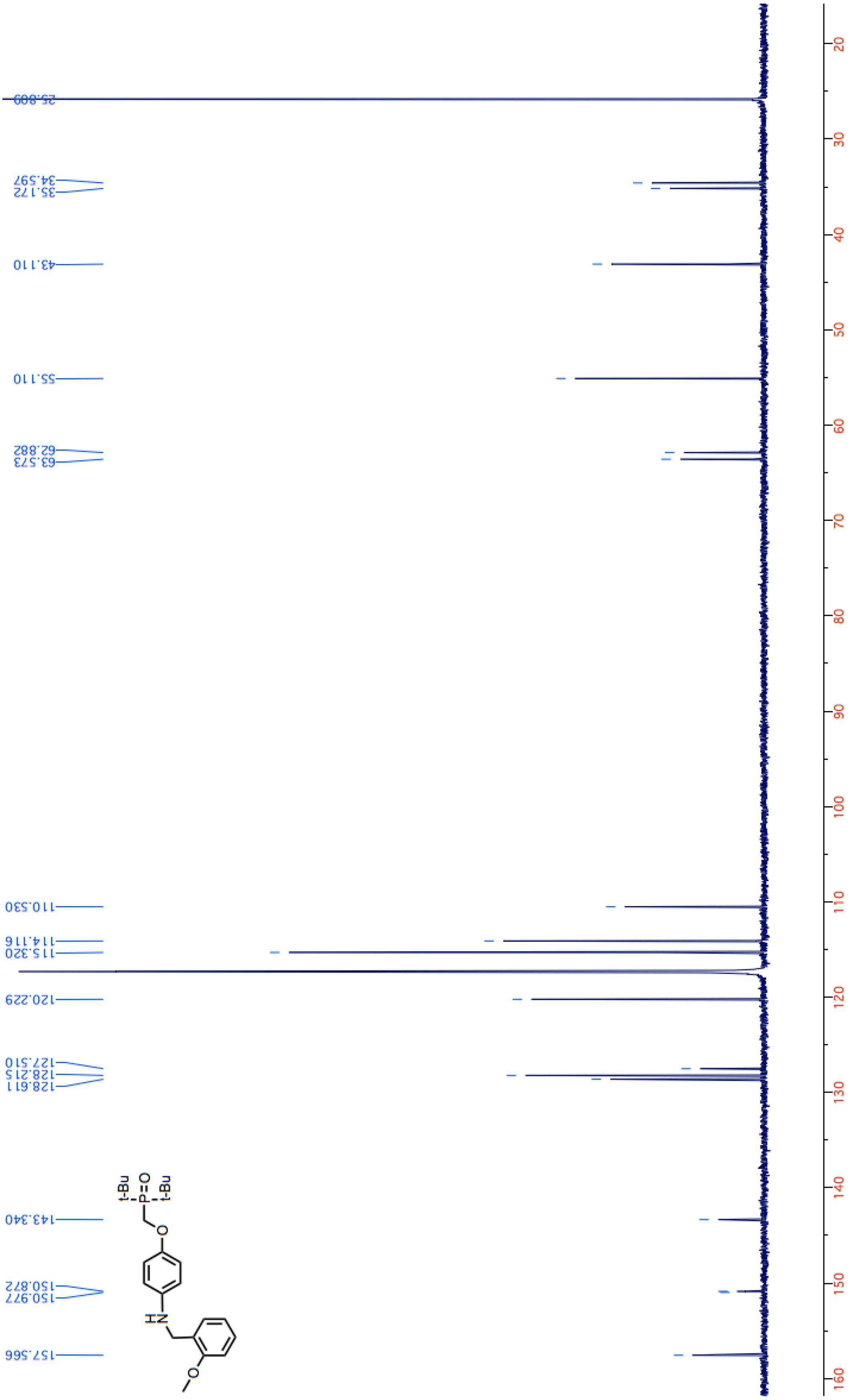
25.809
34.597
35.172
43.110
55.110
62.882
63.573
110.530
114.116
115.320
120.229
127.510
128.215
128.611
143.340
150.872
150.977
157.566
t-Bu
P=O
t-Bu
O
HN
O
20
30
40
50
60
70
80
90
100
110
120
130
140
150
160

Synthesis of 3 [1]

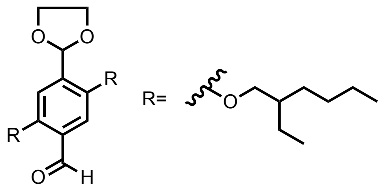

A mixture of **2** (3.0 g, 7.6 mmol), ethane-1,2-diol (0.4 ml, 7.6 mmol) and a catalytic amount of *p*-toluenesulfonic acid in toluene (50 ml) was refluxed for 12 h under nitrogen. After cooling to room temperature, the solution was washed with water (3 x 30 mL) and brine (1 x 30 ml), dried with $MgSO_4$, and the solvent was removed under reduced pressure. The crude product was then purified by column chromatography on silica eluting with hexane/DCM (90:10). The product was isolated as a yellow oil (1.3 g, 40%).

^{1}H NMR (250 MHz, $CDCl_3$): δ 10.47 (s, 1H), 7.32 (s, 1H), 7.20 (s, 1H), 6.10 (s, 1H), 4.19-4.01 (m, 4H), 4.07-3.87 (m, 4H), 1.78-1.71 (m, 2H), 1.51-1.30 (m, 16H), 0.96-0.88 (m, 12H);
^{13}C NMR (62.9 MHz, $CDCl_3$): δ 189.5, 156.3, 151.5, 134.0, 125.6, 111.7, 110.0, 98.7, 71.7, 71.5, 65.4, 39.6, 39.4, 30.7, 30.6, 29.2, 29.0, 24.1, 23.9, 23.1, 23.0, 14.1, 11.2, 11.1;
MS (ES+): m/z (%) = 435.3 (100) [$M+H^+$];
HRMS (ES+): calcd for $C_{26}H_{43}O_5$ 435.3110, found 435.3115;
FT-IR (thin film): ν_{max} /cm^{-1} 2956, 2927, 2873, 2859, 1682.

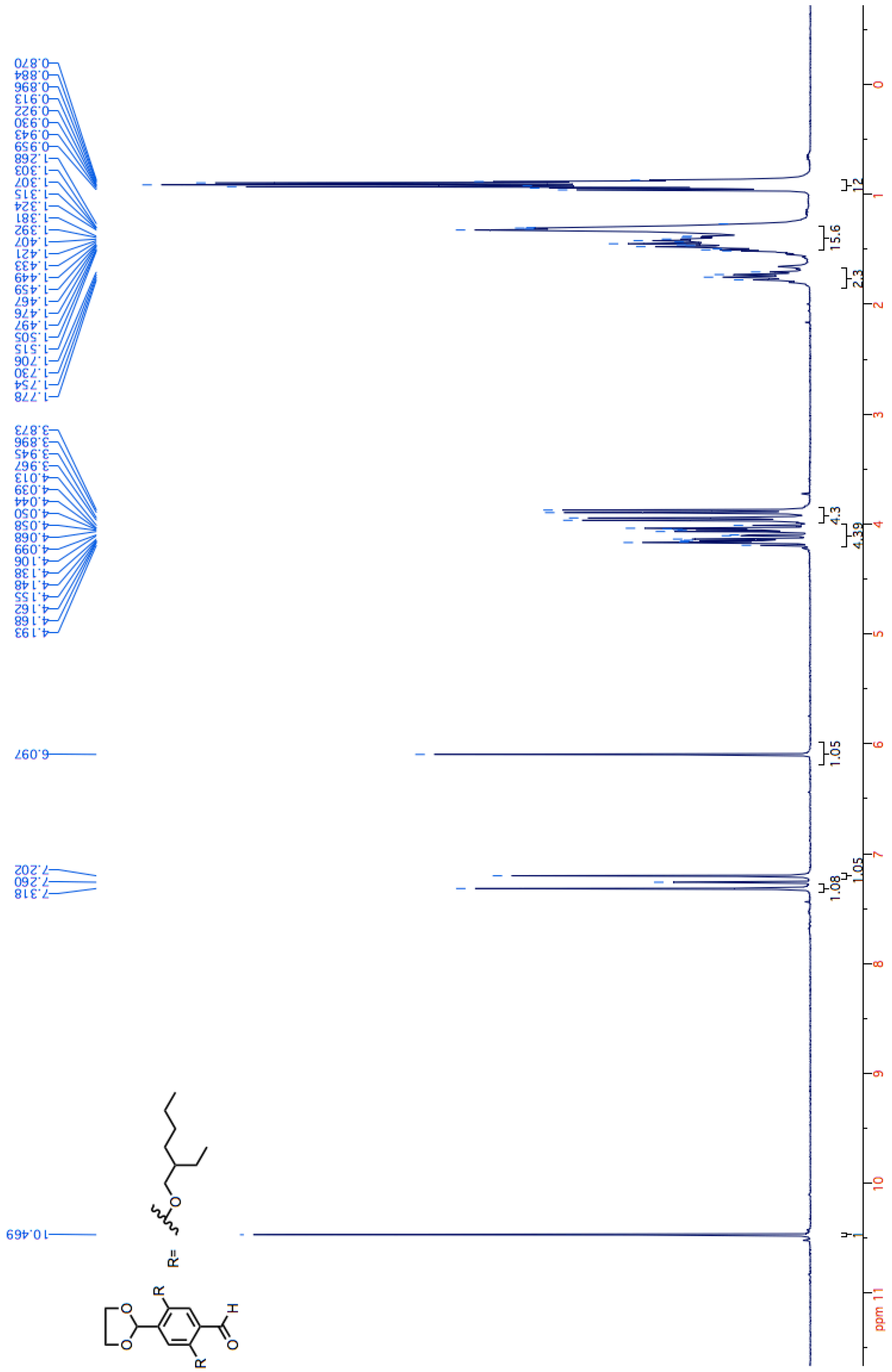

0.870
0.884
0.896
0.913
0.922
0.930
0.943
0.959
1.268
1.303
1.307
1.315
1.324
1.381
1.392
1.407
1.421
1.433
1.449
1.459
1.467
1.476
1.497
1.505
1.515
1.706
1.730
1.754
1.778
3.873
3.896
3.945
3.967
4.013
4.039
4.044
4.050
4.058
4.068
4.099
4.106
4.138
4.148
4.155
4.162
4.168
4.193
6.097
7.202
7.260
7.318
10.469
R=
R
O
H
12
15.6
2.3
4.3
4.39
1.05
1.05
1.08
1
ppm

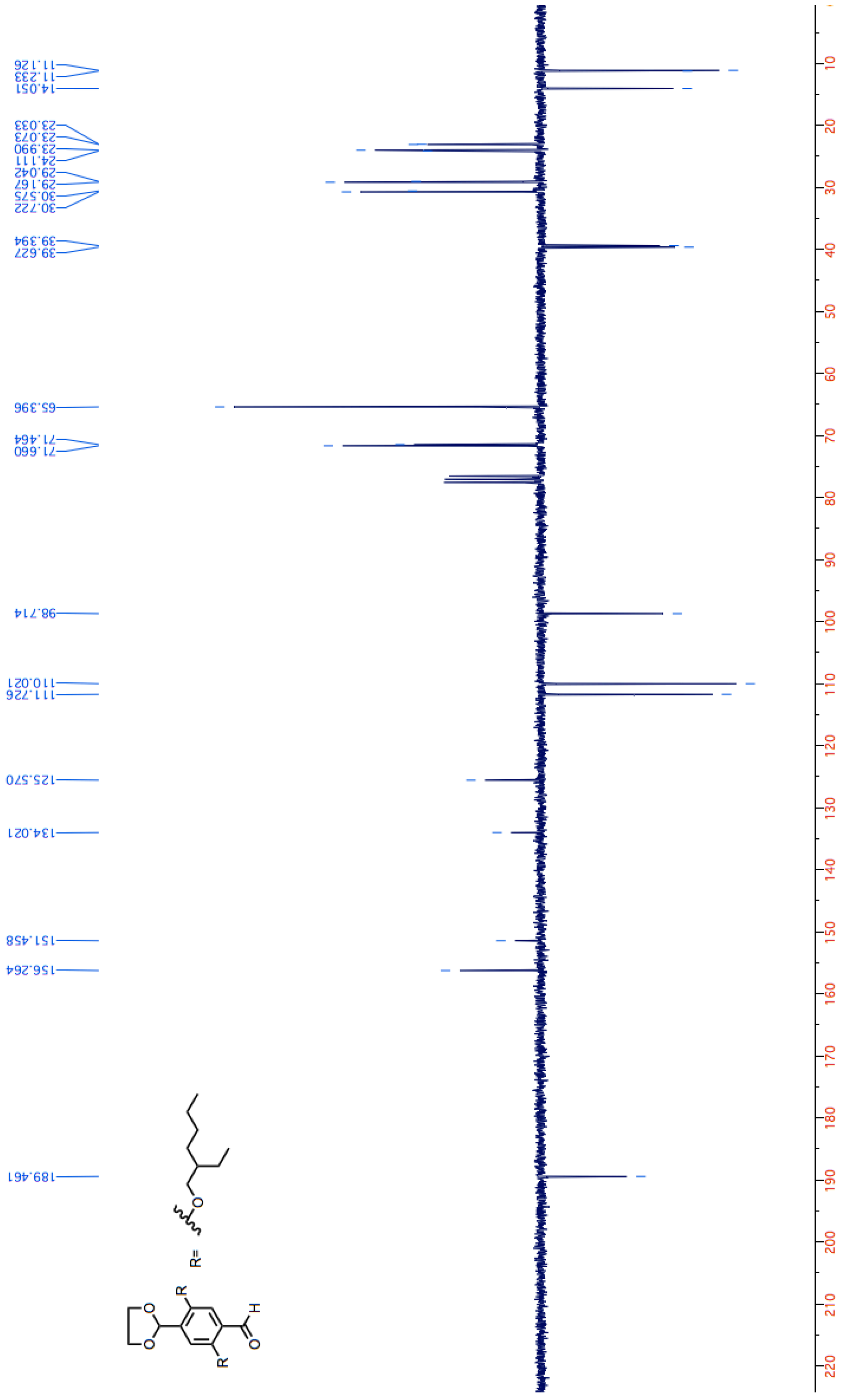
11.126
11.233
14.051
23.033
23.073
23.990
24.111
29.042
29.167
30.575
30.722
39.394
39.627
65.396
71.464
71.660
98.714
110.021
111.726
125.570
134.021
151.458
156.264
189.461
R=
R
R
O
O
O
H
O
10
20
30
40
50
60
70
80
90
100
110
120
130
140
150
160
170
180
190
200
210
220

Synthesis of 4a

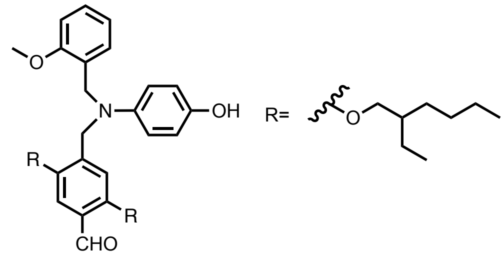

A mixture of **3** (0.66 g, 1.5 mmol), **1a** (0.17 g, 0.74 mmol) and $NaBH(AcO)_3$ (0.45 g, 5.9 mmol) in DCE (2.5 ml) dried with molecular sieves, was stirred under nitrogen at room temperature for 4 h. The solution was then washed with saturated aqueous $NaHCO_3$ (1 x 10 ml), water (1 x 10 ml) and brine (1 x 10 ml) and the solvent was removed under reduced pressure. The crude product was then diluted with DCM (10ml) and left stirring with HCl 4M (10ml) for 12 h at room temperature. The solution was then washed with saturated aqueous $NaHCO_3$ (1 x 20 ml), water (1 x 20 ml) and brine (1 x 20 ml), dried with $MgSO_4$, and the solvent was removed under reduced pressure then purified by column chromatography on silica eluting with $CHCl_3$/MeOH (99:1). The product was isolated as a yellow oil (0.42 g, 94%).

^{1}H NMR (500 MHz, CD_3CN): δ 10.35 (s, 1H), 7.22-7.15 (m, 3H), 6.94-6.91 (m, 2H), 6.84 (td, J = 7, J = 1, 1H), 6.61-6.52 (m, 4H), 6.27 (bs, 1H), 4.55-4.54 (m, 4H), 3.88 (d, 2H), 3.77 (s, 3H), 3.74 (d, J = 6, 2H), 1.69-1.58 (m, 2H), 1.48-1.23 (m, 16H), 0.91-0.83 (m, 12H);
^{13}C NMR (125.7 MHz, CD_3CN): δ 189.5, 158.4, 157.3, 151.7, 149.5, 143.5, 138.4, 128.9, 128.8, 127.7, 124.6, 121.1, 116.7, 115.3, 114.3, 111.5, 109.2, 72.4, 71.6, 56.0, 52.0, 51.5, 40.3, 40.0, 31.5, 31.2, 29.8, 29.7, 24.8, 24.6, 23.7, 23.7, 14.4, 14.4, 11.6, 11.4;
MS (ES+): m/z (%) = 604.4 (100) [$M+H^+$];
HRMS (ES+): calcd for $C_{38}H_{54}NO_5$ 604.4002, found 604.4016;
FT-IR (thin film): ν_{max} /cm^{-1} 2961, 2928, 2864, 1661, 1492, 1489, 1515

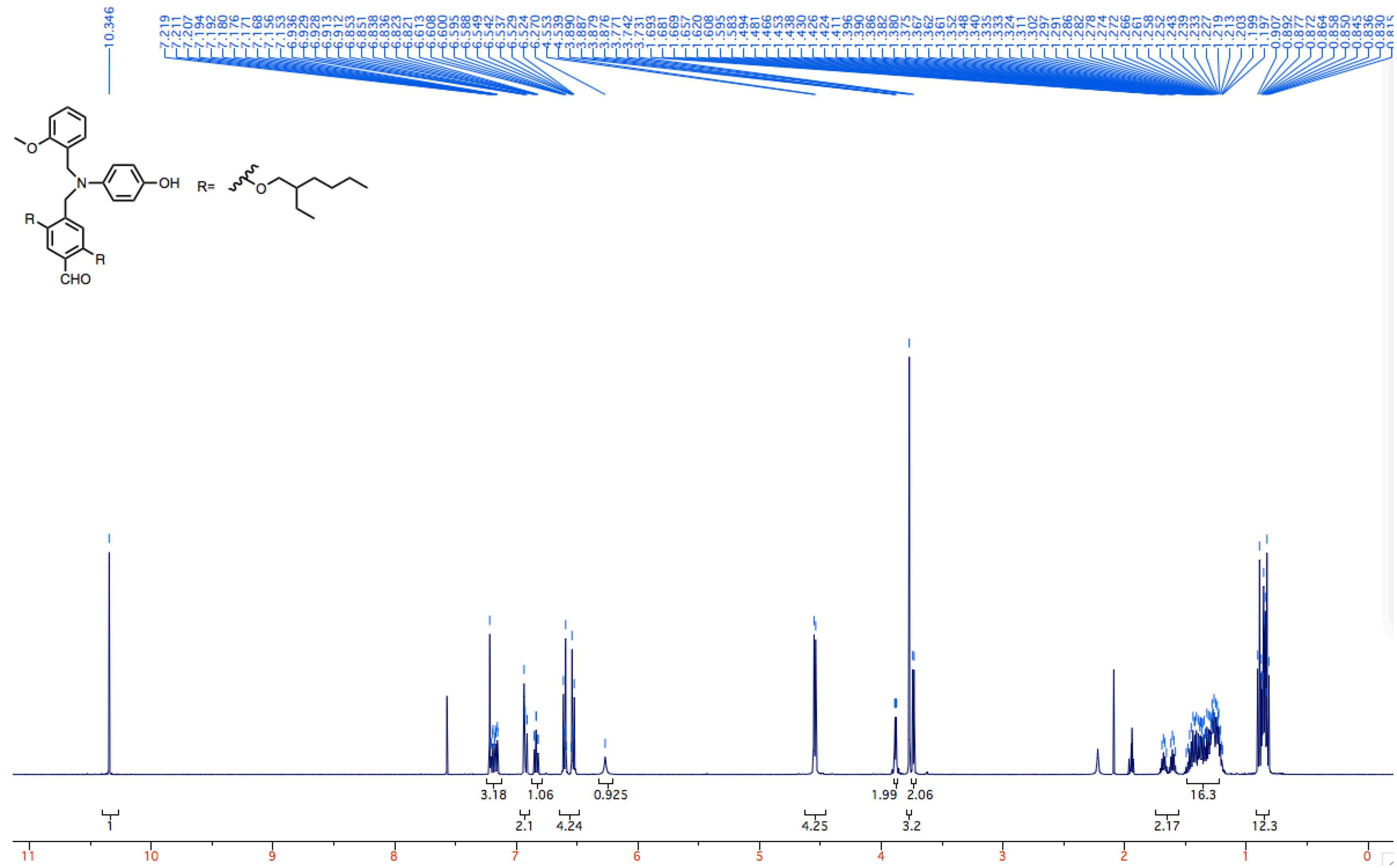
O
N
OH
R
R
CHO
R=
O
10.346
7.219
7.211
7.207
7.194
7.192
7.180
7.176
7.171
7.168
7.156
7.153
6.936
6.929
6.928
6.913
6.912
6.853
6.851
6.838
6.836
6.823
6.821
6.613
6.608
6.600
6.595
6.588
6.549
6.542
6.537
6.529
6.524
6.270
4.553
4.539
3.890
3.887
3.879
3.876
3.771
3.742
3.731
1.693
1.681
1.669
1.657
1.620
1.608
1.595
1.583
1.494
1.481
1.466
1.453
1.438
1.430
1.426
1.424
1.411
1.396
1.390
1.386
1.382
1.380
1.375
1.367
1.362
1.361
1.352
1.348
1.340
1.335
1.333
1.324
1.311
1.302
1.297
1.291
1.286
1.282
1.278
1.274
1.272
1.266
1.261
1.258
1.252
1.243
1.239
1.233
1.227
1.219
1.213
1.203
1.199
1.197
0.907
0.892
0.877
0.872
0.864
0.858
0.850
0.845
0.836
0.830
0.815
3.18
1.06
0.925
1.99
2.06
16.3
1
2.1
4.24
4.25
3.2
2.17
12.3
11
10
9
8
7
6
5
4
3
2
1
0

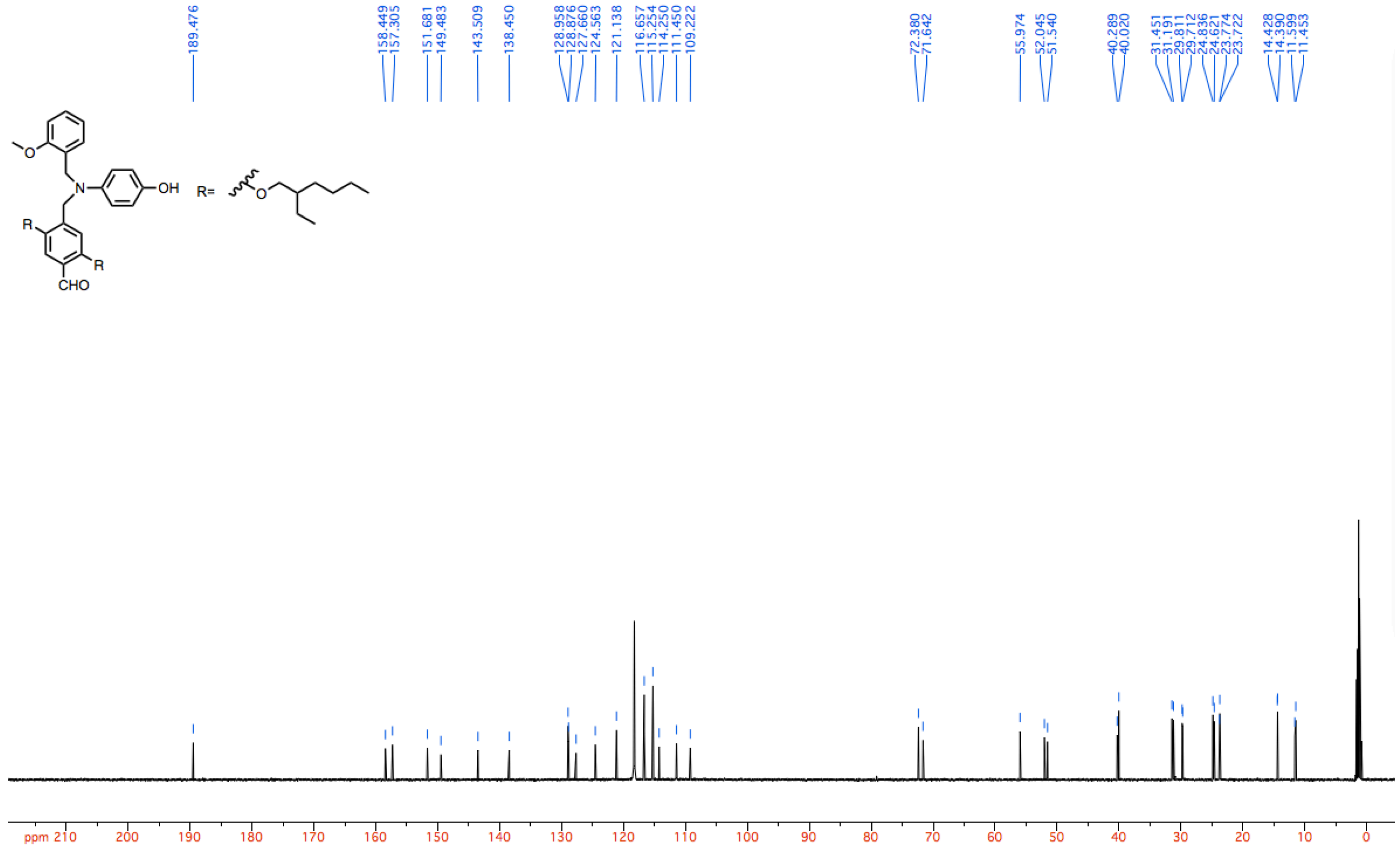

189.476
158.449
157.305
151.681
149.483
143.509
138.450
128.958
128.876
127.660
124.563
121.138
116.657
115.254
114.250
111.450
109.222
72.380
71.642
55.974
52.045
51.540
40.289
40.020
31.451
31.191
29.811
29.712
24.836
24.621
23.774
23.722
14.428
14.390
11.599
11.453
OH
R=
O
R
CHO
N
ppm 210
200
190
180
170
160
150
140
130
120
110
100
90
80
70
60
50
40
30
20
10
0

Synthesis of 4b

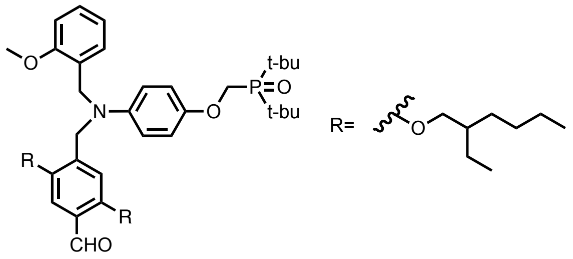

A mixture of **3** (0.19 g, 0.45 mmol), **1b** (0.09 g, 0.22 mmol) and $NaBH(AcO)_3$ (0.13 g, 0.60 mmol) in DCE (0.7 ml) dried with molecular sieves, was stirred under nitrogen at room temperature for 12 h. The solution was then washed with saturated aqueous $NaHCO_3$ (1 x 10 ml), water (1 x 10 ml) and brine (1 x 10 ml), dried with $MgSO_4$, and the solvent was removed under reduced pressure. The crude product was then diluted with DCM (10 ml) and left stirring with HCl 4M (10 ml) for 12 h at room temperature. The solution was then washed with saturated aqueous $NaHCO_3$ (1 x 20 ml), water (1 x 20 ml) and brine (1 x 20 ml) and the solvent was removed under reduced pressure then purified by column chromatography on silica eluting with $CHCl_3$/MeOH (99:1). The product was isolated as an orange oil (0.93 g, 60%).

^{1}H NMR (400 MHz, aceton-*d*$_6$): δ 10.42 (s, 1H), 7.28 (s, 1H), 7.24 (t, J = 8, 2H), 7.03-7.01 (m, 2H), 6.92-6.89 (m, 3H), 6.69 (d, J = 9, 2H), 4.7-4.3 (m, 4H), 4.30 (d, J = 7, 2H), 4.00 (d, J = 5, 2H), 3.87 (s, 3H), 3.84 (d, J = 6, 2H), 1.81-171 (m, 1H), 1.71-1.65 (m, 1H), 1.53-1.28 (m, 34H), 0.98-0.86 (m, 12H);

^{31}P NMR (162.0 MHz, aceton-*d*$_6$): δ 53.8;

^{13}C NMR (100.6 MHz, aceton-*d*$_6$): δ 187.8, 157.5, 156.3, 150.7, 150.7, 150.7, 143.9, 136.9, 128.0, 127.7, 126.3, 123.8, 120.2, 115.1, 113.7, 113.1, 110.4, 108.3, 71.3, 70.6, 63.7, 63.0, 54.8, 51.1, 50.5, 39.4, 39.1, 35.2, 34.7, 30.6, 30.3, 28.1, 25.9, 23.9, 23.7, 22.8, 22.7, 13.5, 13.4, 10.6, 10.5;

MS (ES+): m/z (%) = 778.5 (100) [M+H$^+$], 800.5 (10) [M+Na];

HRMS (ES+): calcd for $C_{47}H_{73}NO_6P$ 778.5176, found 778.5142.

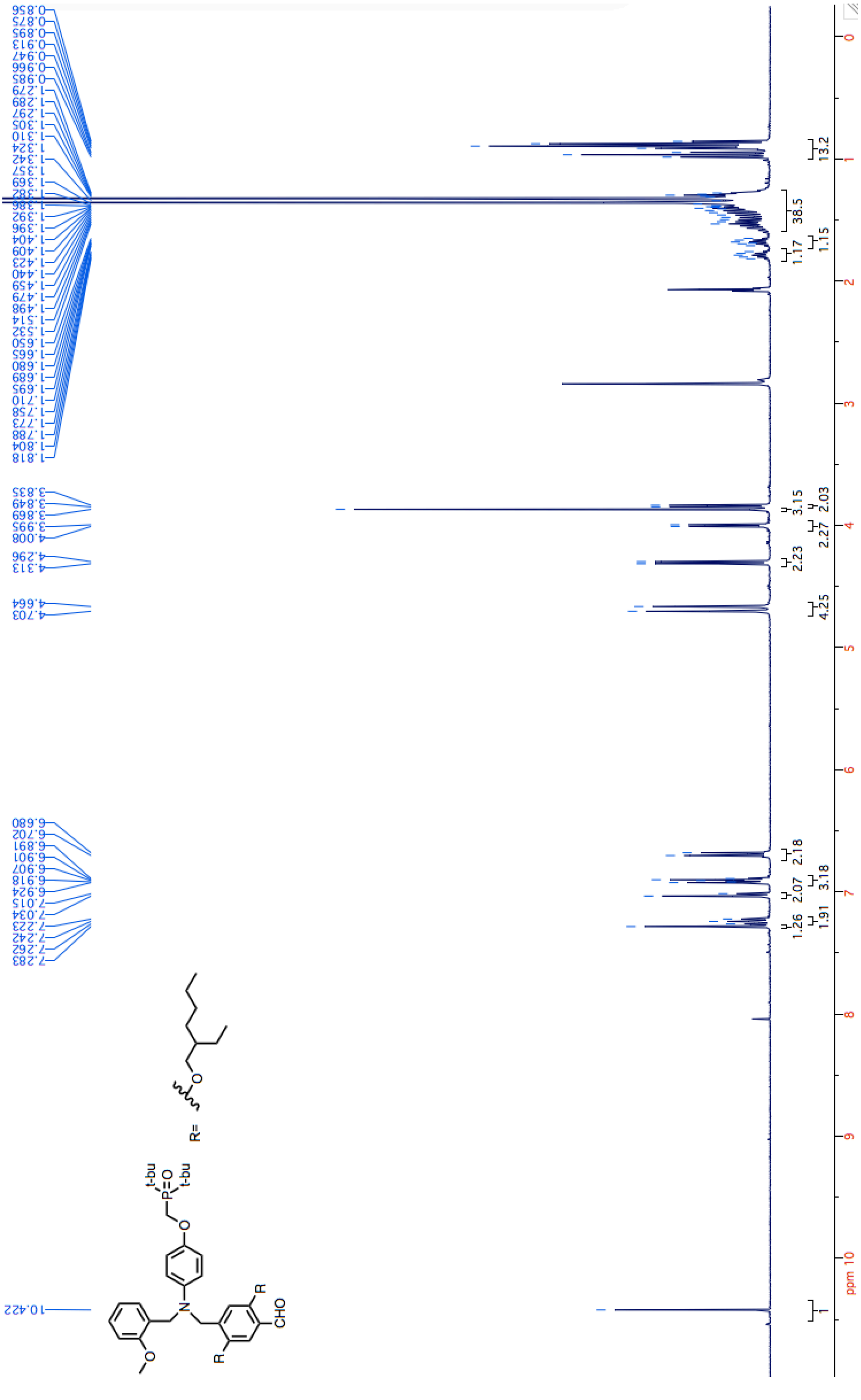

0.856
0.875
0.895
0.913
0.947
0.966
0.985
1.279
1.289
1.297
1.305
1.310
1.324
1.342
1.357
1.369
1.382
1.386
1.392
1.396
1.404
1.409
1.423
1.440
1.459
1.479
1.498
1.514
1.532
1.650
1.665
1.680
1.689
1.695
1.710
1.758
1.773
1.788
1.804
1.818
3.835
3.849
3.869
3.995
4.008
4.296
4.313
4.664
4.703
6.680
6.702
6.891
6.901
6.907
6.918
6.924
7.015
7.034
7.223
7.242
7.262
7.283
10.422
t-bu
P=O
t-bu
N
R
CHO
R=
13.2
38.5
1.17
1.15
3.15
2.03
2.27
2.23
4.25
2.18
2.07
3.18
1.26
1.91
1
ppm

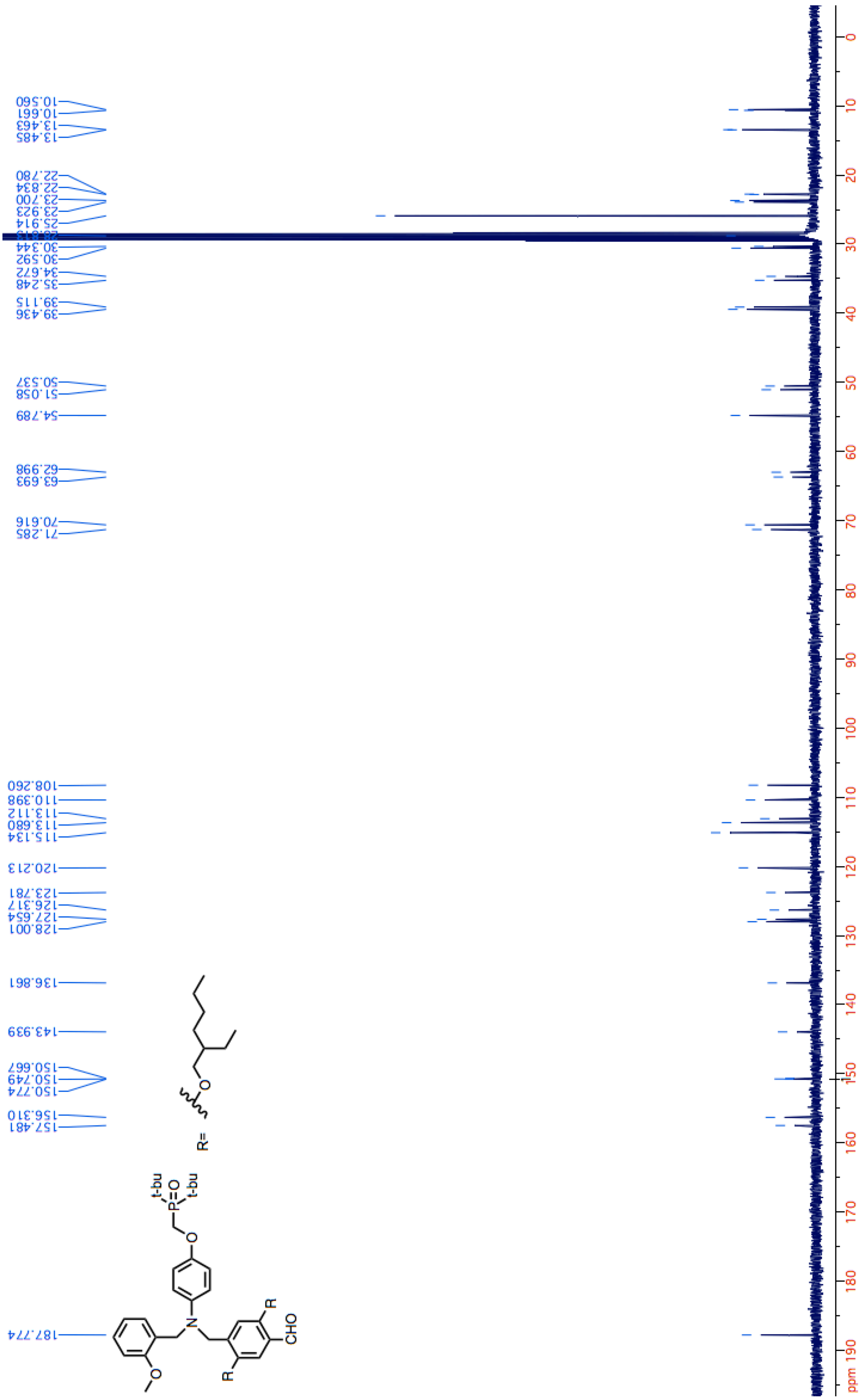
10.560
10.661
13.463
13.485
22.780
22.834
23.700
23.923
25.914
30.344
30.592
34.672
35.248
39.115
39.436
50.537
51.058
54.789
62.998
63.693
70.616
71.285
108.260
110.398
113.112
113.680
115.134
120.213
123.781
126.317
127.654
128.001
136.861
143.939
150.667
150.749
150.774
156.310
157.481
187.774
R=
t-bu
t-bu
CHO
ppm

Synthesis of 5a

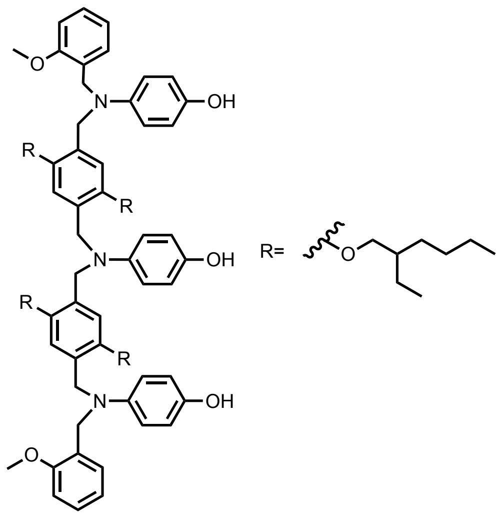

A mixture of **4a** (90 mg, 0.140 mmol), of *para*-aminophenol (4.0 g, 0.036 mmol) and $NaBH(AcO)_3$ (44 mg, 0.09 mmol), AcOH (4 eq) in DCE (100 µl) dried with molecular sieves, was stirred under nitrogen at room temperature for 24 h. The solution was then washed with saturated aqueous $NaHCO_3$ (1 x 10 ml), water (1 x 10 ml) and brine (1 x 10 ml), dried with $MgSO_4$, and the solvent was removed under reduced pressure then purified by column chromatography on silica eluting with hexane/Et_2O (60:40). The product was isolated as a brown oil (26 mg, 56%).

^{1}H NMR (500 MHz, CD_3CN): δ 7.18 (td, J = 8, J = 1, 2H), 7.12 (d, J = 7, 2H), 6.92 (d, J = 8, 2H), 6.82 (td, J = 7, J = 1, 2H), 6.74 (s, 2H), 6.72 (s, 2H) 6.58-6.51 (m, 12H), 6.15 (bs, 3H), 4.48-4.46 (m, 12H), 3.77 (s, 6H), 3.63-3.60 (m, 8H), 1.57-1.53 (m, 4H), 1.38-1.20 (m, 32H), 0.83-0.78 (m, 24H);

^{13}C NMR (125.7 MHz, CD_3CN): δ 158.4, 151.5, 151.4, 149.4, 149.2, 144.0, 143.9, 128.7, 128.6, 127.9, 127.4, 121.1, 116.6, 116.5, 115.5, 115.1, 113.1, 113.0, 111.4, 71.8, 56.0, 51.4, 51.2, 51.2, 40.2, 31.4, 31.4, 29.8, 24.8, 24.7, 23.8, 23.7, 14.4, 14.4, 11.6, 11.5;

MS (ES+): m/z (%) = 1284.8 (100) [M+H$^+$];

HRMS (ES+): calcd for $C_{82}H_{114}N_3O_9$ 1284.8555, found 1284.8569;

FT-IR (thin film): ν_{max} /cm^{-1} 3368, 2927, 2871, 1602, 150.

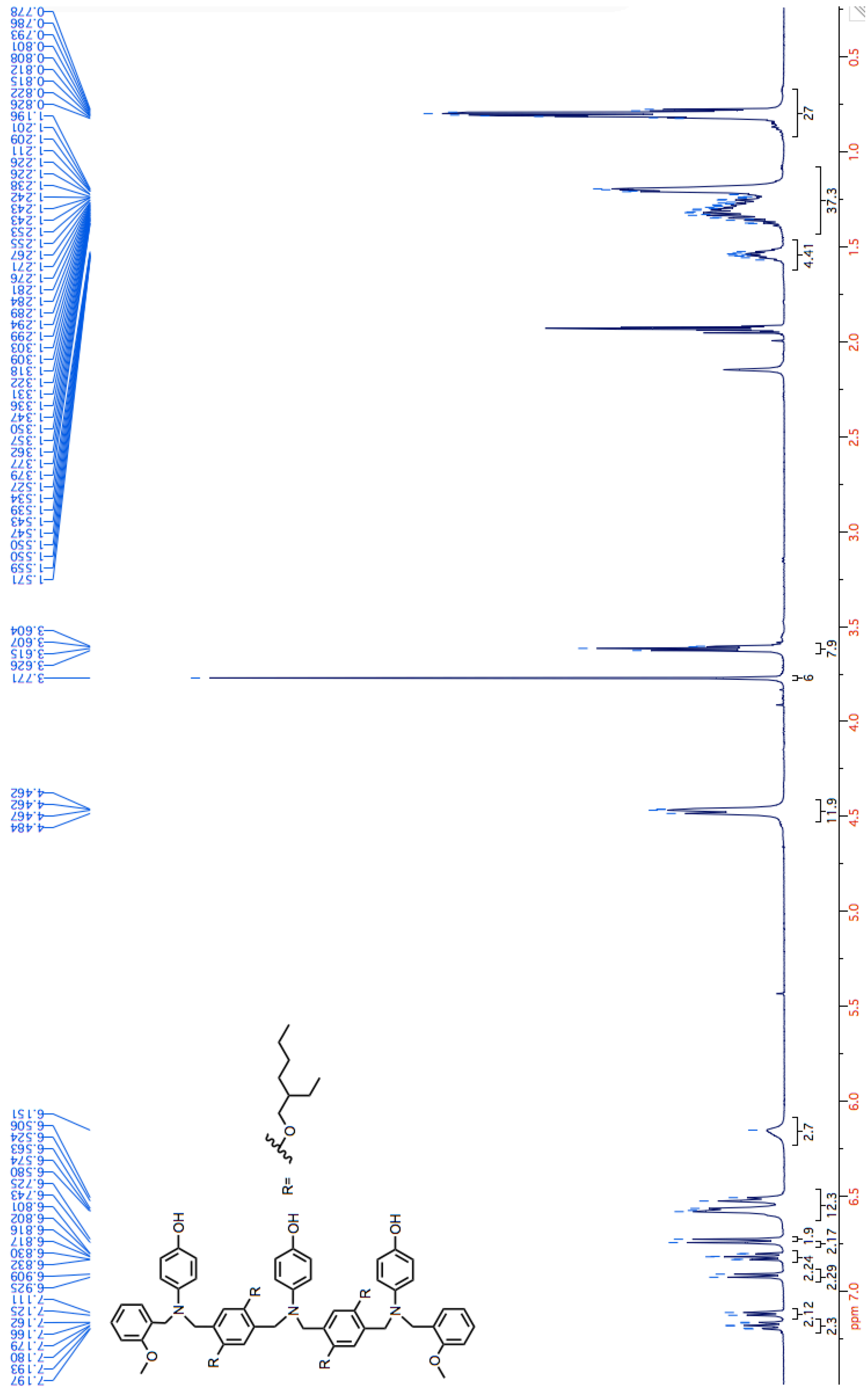
0.778
0.786
0.793
0.801
0.808
0.812
0.815
0.822
0.826
1.196
1.201
1.209
1.211
1.226
1.226
1.238
1.242
1.243
1.243
1.253
1.255
1.267
1.271
1.276
1.281
1.284
1.289
1.294
1.299
1.303
1.309
1.318
1.322
1.331
1.336
1.347
1.350
1.357
1.362
1.377
1.379
1.527
1.534
1.539
1.543
1.547
1.550
1.550
1.559
1.571
3.604
3.607
3.615
3.626
3.771
4.462
4.462
4.467
4.484
6.151
6.506
6.524
6.563
6.574
6.580
6.725
6.743
6.801
6.802
6.816
6.817
6.830
6.832
6.909
6.925
7.111
7.125
7.162
7.166
7.179
7.180
7.193
7.197
R=
OH
N
R
O
27
37.3
4.41
7.9
6
11.9
2.7
12.3
1.9
2.17
2.24
2.29
2.12
2.3
0.5
1.0
1.5
2.0
2.5
3.0
3.5
4.0
4.5
5.0
5.5
6.0
6.5
ppm 7.0

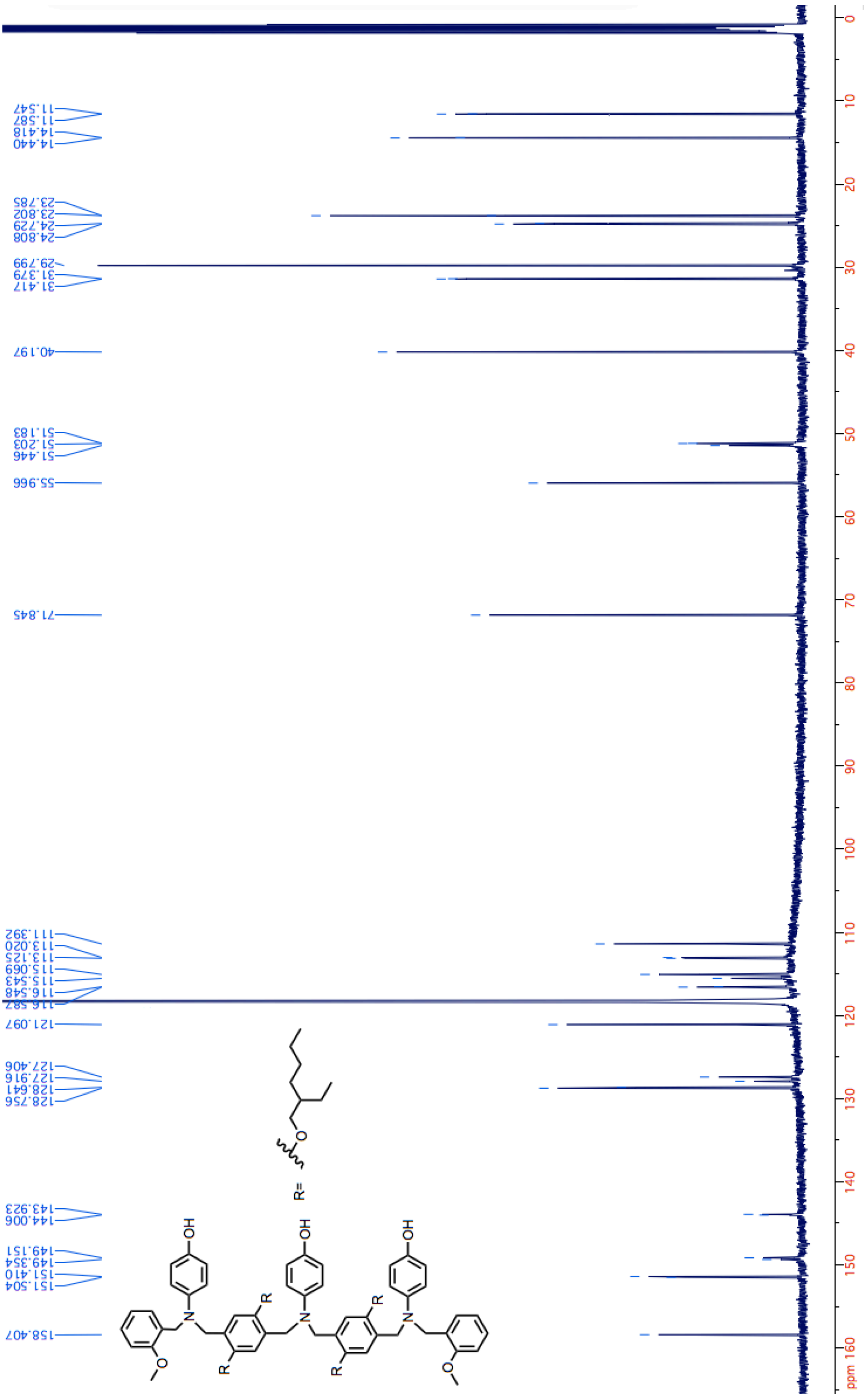

11.547
11.587
14.418
14.440
23.785
23.802
24.729
24.808
29.799
31.379
31.417
40.197
51.183
51.203
51.446
55.966
71.845
111.392
113.020
113.125
115.069
115.543
116.548
116.587
121.097
127.406
127.916
128.641
128.756
143.923
144.006
149.151
149.354
151.410
151.504
158.407
R=
ppm

Synthesis of 5b

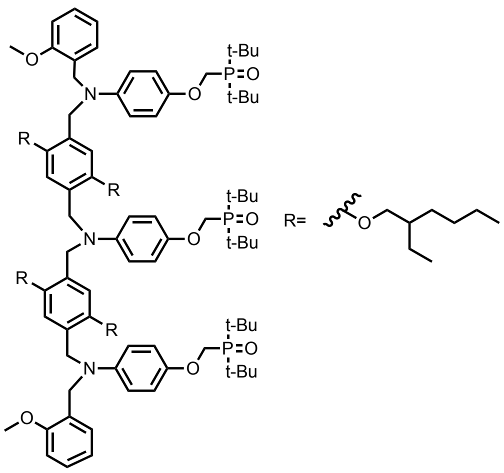

A mixture of **4b** (130 mg, 0.16 mmol), of di-*tert*-butyl((4-((2-methoxybenzyl)amino)phenoxy)methyl)phosphine oxide (12.0 mg, 0.04 mmol), $NaBH(AcO)_3$ (50.0 mg, 0.20 mmol) and AcOH (4 eq) in DCE (100 µl) dried with molecular sieves, was stirred under nitrogen at room temperature for 24 h. The solution was then washed with saturated aqueous $NaHCO_3$ (1 x 10 ml), water (1 x 10 ml) and brine (1 x 10 ml), dried with $MgSO_4$, and the solvent was removed under reduced pressure then purified by column chromatography on silica eluting with EtOAc/MeOH (90:10). The product was isolated as an orange oil (13.0 mg, 18%).

^{1}H NMR (500 MHz, CD_3CN): δ 7.21-7.17 (m, 2H), 7.10-7.08 (m, 2H), 6.94-6.92 (m, 2H), 6.83-6.76 (m, 8H), 6.70 (d, J = 8, 4H), 6.63 (d, J = 9, 2H), 6.57 (d, J = 9, 4H), 4.54 (s, 4H), 4.51 (s, 8H), 4.23 (d, J = 7, 6H), 3.78 (s, 6H), 3.62-3.60 (m, 8H), 1.54-1.50 (m, 4H), 1.33-1.17 (m, 86H), 0.81-0.76 (m, 24H);

^{31}P NMR (202.4 MHz, CD_3CN): δ 55.3;

^{13}C NMR (125.7 MHz, CD_3CN): δ 158.4, 151.4, 151.3, 151.3, 151.3, 151.2, 151.1, 145.1, 145.0, 128.9, 128.5, 127.6, 127.2, 121.1, 116.0, 116.0, 114.8, 114.5, 113.1, 112.9, 111.5, 71.8, 71.8, 64.4, 64.1, 63.8, 63.8, 56.0, 51.3, 51.2, 51.1, 40.1, 40.1, 36.1, 35.6, 31.3, 31.3, 29.8, 26.8, 24.8, 24.7, 23.8, 23.8, 14.5, 14.5, 11.6, 11.6;

MS (ES+): m/z (%) = 1807.2 (70) [M+H$^+$], 1830.2 (100) [M+Na];

HRMS (ES+): calcd for $C_{109}H_{171}N_3O_{12}P_3$ 1807.2076, found 1807.2063;

FT-IR (thin film): ν_{max} /cm^{-1} 2957, 2927, 2871, 1680, 1601, 1511,

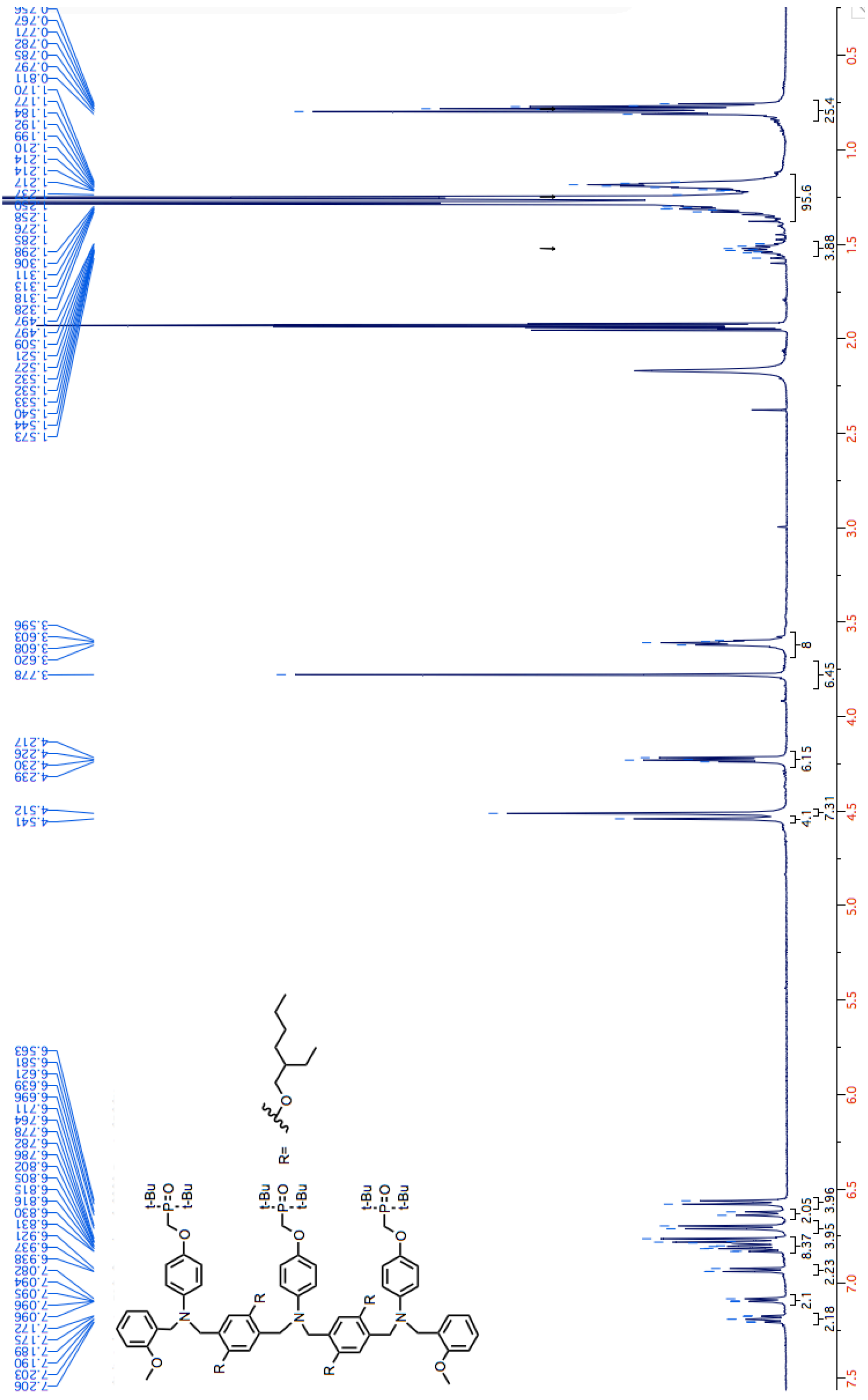
0.756
0.767
0.771
0.782
0.785
0.797
0.811
1.170
1.177
1.184
1.192
1.199
1.210
1.214
1.214
1.217
1.237
1.250
1.258
1.276
1.285
1.298
1.306
1.311
1.313
1.318
1.328
1.497
1.497
1.509
1.521
1.527
1.532
1.532
1.533
1.540
1.544
1.573
3.596
3.603
3.608
3.620
3.778
4.217
4.226
4.230
4.239
4.512
4.541
6.563
6.581
6.621
6.639
6.696
6.711
6.764
6.778
6.782
6.786
6.802
6.805
6.815
6.816
6.830
6.831
6.921
6.937
6.938
7.082
7.094
7.095
7.096
7.096
7.172
7.175
7.189
7.190
7.203
7.206
25.4
95.6
3.88
8
6.45
6.15
4.1
7.31
3.96
2.05
8.37
3.95
2.23
2.1
2.18
0.5
1.0
1.5
2.0
2.5
3.0
3.5
4.0
4.5
5.0
5.5
6.0
6.5
7.0
7.5
t-Bu
P=O
t-Bu
O
N
R
R=
O

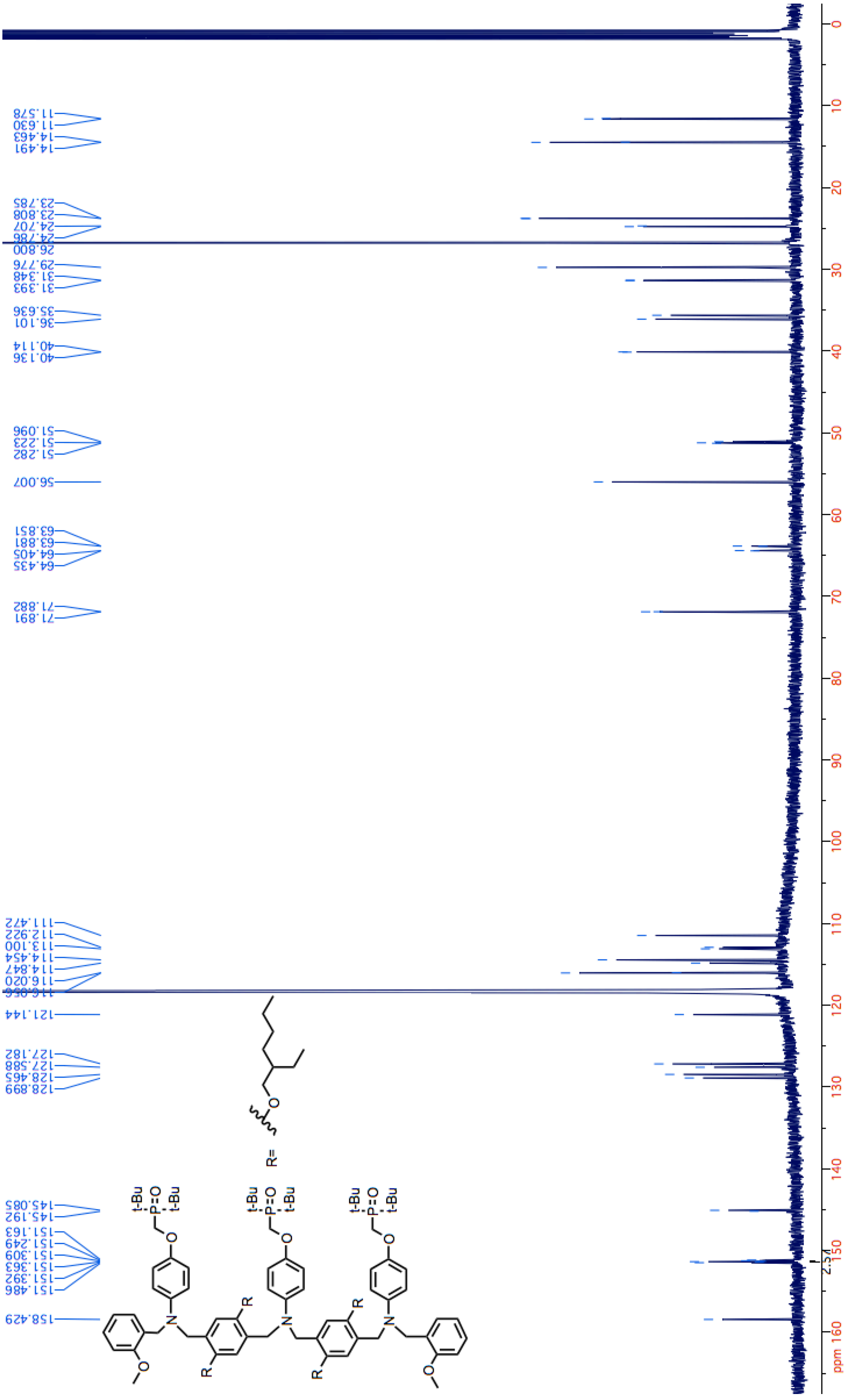
11.578
11.630
14.463
14.491
23.785
23.808
24.707
24.786
26.800
29.776
31.348
31.393
35.636
36.101
40.114
40.136
51.096
51.223
51.282
56.007
63.851
63.881
64.405
64.435
71.882
71.891
111.472
112.922
113.100
114.454
114.847
116.020
121.144
127.182
127.588
128.465
128.899
145.085
145.192
151.163
151.249
151.309
151.363
151.392
151.486
158.429
t-Bu
R=
ppm

Synthesis of 7a

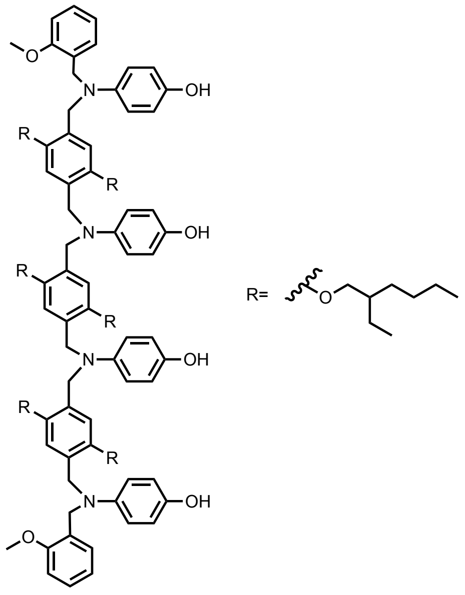

A mixture of **4a** (0.390 g, 0.64 mmol), of **6a** (0.093 g, 0.16 mmol) and $NaBH(AcO)_3$ (0.190 g, 0.9 mmol), AcOH (4 eq) in DCE (1 ml) dried with molecular sieves, was stirred under nitrogen at room temperature for 6 h. The solution was then washed with saturated aqueous $NaHCO_3$ (1 x 10 ml), water (1 x 10 ml) and brine (1 x 10 ml), dried with $MgSO_4$, and the solvent was removed under reduced pressure then purified by column chromatography on silica eluting with hexane/Et_2O (60:40). The product was isolated as a brown oil (0.203 g, 72%).

^{1}H NMR (500 MHz, CD_3CN): δ 7.17 (td, J = 8, J = 1, 2H), 7.11 (dd, J = 7, J = 1, 2H), 6.91-6.89 (m, 2H), 6.81 (td, J = 7, J = 1, 2H), 6.74-6.72 (m, 6H), 6.59-6.50 (m, 16H), 6.20 (bs, 4H), 4.47-4.46 (m, 16H), 3.76 (s, 6H), 3.63-3.59 (m, 12H), 1.56-1.50 (m, 6H), 1.38-1.16 (m, 48H), 0.81-0.76 (m, 36H);

^{13}C NMR (125.7 MHz, CD_3CN): δ 158.4, 151.5, 151.4, 151.3, 149.3, 149.1, 144.0, 143.9, 128.7, 128.5, 127.9, 127.3, 127.3, 121.1, 116.5, 116.5, 115.5, 115.0, 113.0, 113.0, 112.9, 111.3, 71.8, 55.9, 51.4, 51.2, 51.1, 51.1, 40.2, 40.1, 40.1, 31.4, 31.4, 31.3, 29.8, 24.7, 24.7, 23.7, 23.7, 14.4, 14.4, 11.5, 11.5;

MS (ES+): m/z (%) = 1753.2 (100) [$M+H^+$];

HRMS (ES+): calcd for $C_{112}H_{159}N_4O_{12}$ 1752.1955, found 1752.1964;

FT-IR (thin film): ν_{max} /cm^{-1} 3625, 3541, 3441, 2964, 2929, 2867, 1500.

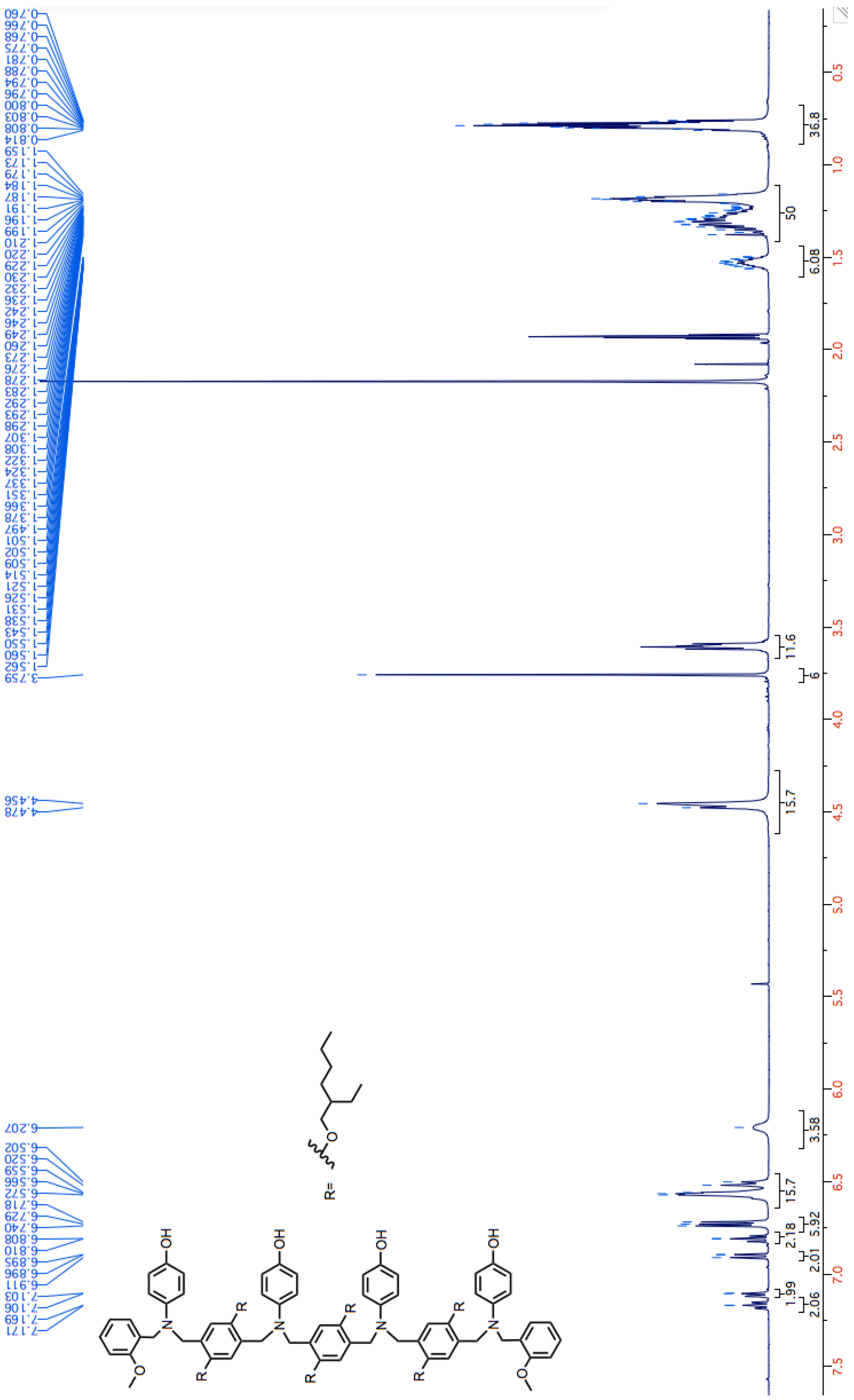

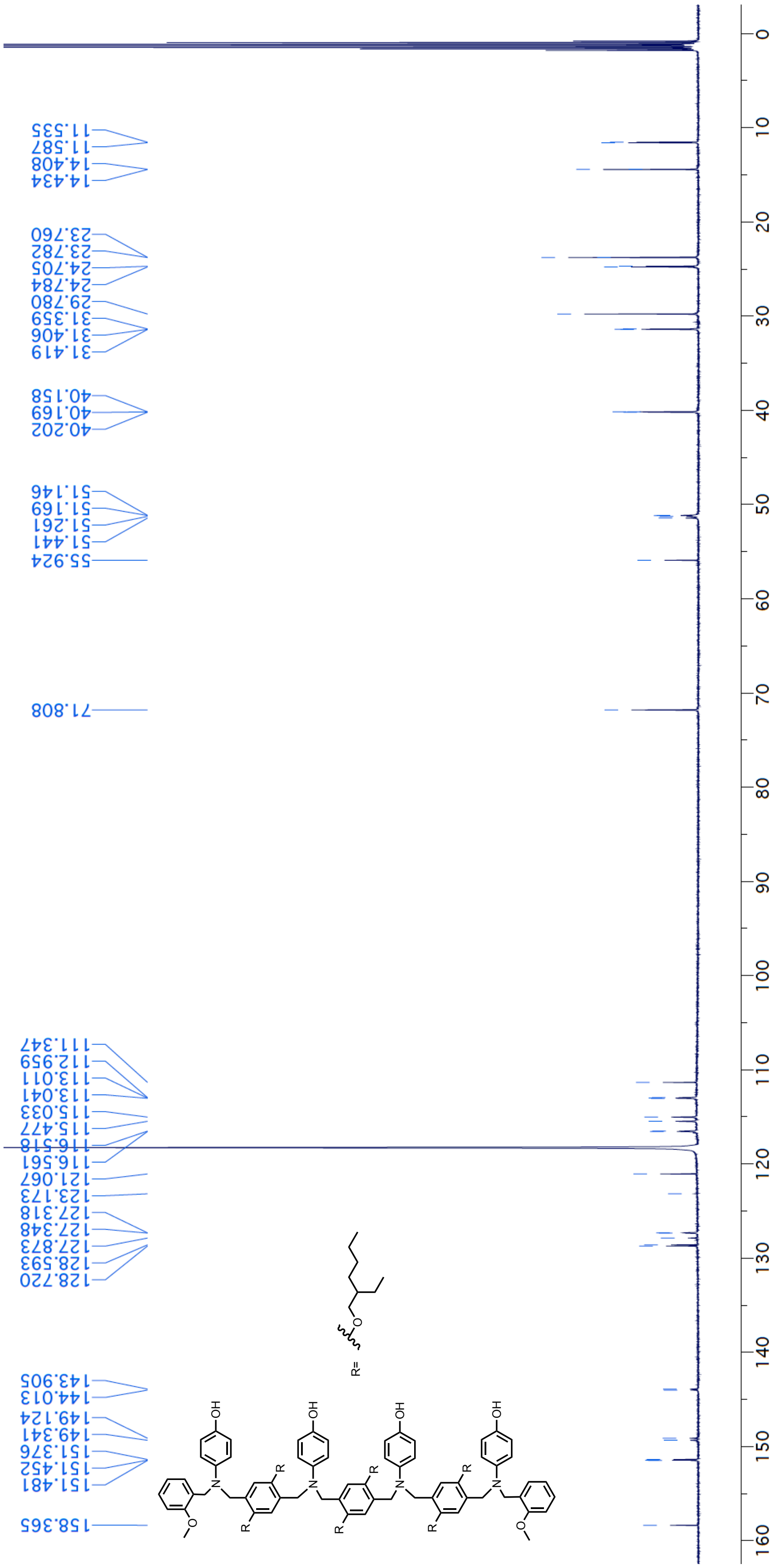
11.535
11.587
14.408
14.434
23.760
23.782
24.705
24.784
29.780
31.359
31.406
31.419
40.158
40.169
40.202
51.146
51.169
51.261
51.441
55.924
71.808
111.347
112.959
113.011
113.041
115.033
115.477
116.518
116.561
121.067
123.173
127.318
127.348
127.873
128.593
128.720
143.905
144.013
149.124
149.341
151.376
151.452
151.481
158.365
R=

Synthesis of 7b

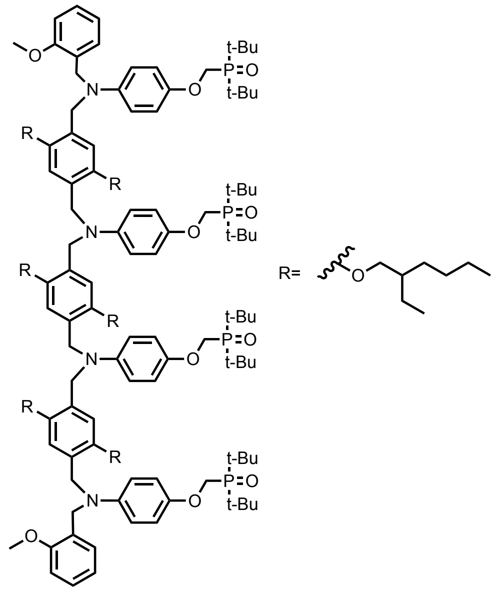

A mixture of **4b** (60 mg, 0.07 mmol), of **6b** (18 mg, 0.02 mmol) and NaBH(AcO)$_3$ (23 mg, 0.1 mmol), AcOH (4 eq) in DCE (100 µl) dried with molecular sieves, was stirred under nitrogen at room temperature for 24 h. The solution was then washed with saturated aqueous NaHCO$_3$ (1 x 10 ml), water (1 x 10 ml) and brine (1 x 10 ml), dried with MgSO$_4$, and the solvent was removed under reduced pressure then purified by column chromatography on silica eluting with EtOAc/MeOH (90:10). The product was isolated as an orange oil (10 mg, 20%).

^{1}H NMR (500 MHz, CD$_3$CN): δ 7.19 (td, J = 8, J = 1, 2H), 7.09 (dd, J = 7, J = 1, 2H), 6.93-6.92 (m, 2H), 6.83-6.76 (m, 10H), 6.71-6.69 (m, 6H), 6.64-6.57 (m, 8H), 4.53-4.51 (m, 16H), 4.23 (d, J = 7, 8H), 3.78 (s, 6H), 3.62-3.59 (m, 12H), 1.53-1.51 (m, 6H), 1.32-1.16 (m, 120H), 0.79-0.76 (m, 36H);
^{31}P NMR (202.4 MHz, CD$_3$CN): δ 55.4;
^{13}C NMR (125.7 MHz, CD$_3$CN): δ 157.3, 150.3, 150.3, 150.2, 150.2, 150.2, 150.1, 150.0, 144.1, 143.9, 127.7, 127.3, 126.4, 126.0, 125.9, 125.9, 120.0, 114.9, 114.8, 113.7, 113.3, 111.8, 111.7, 110.3, 70.7, 63.3, 63.2, 62.7, 62.7, 54.9, 50.2, 50.1, 50.0, 49.9, 39.0, 39.0, 38.9, 35.0, 34.5, 30.2, 30.2, 28.7, 25.7, 23.6, 23.6, 23.5, 22.6, 22.6, 13.3, 13.3, 10.5, 10.4;
MALDI MS: m/z (%) = 2448.9 (100) [M+H$^+$], calcd for $C_{148}H_{234}N_4O_{16}P_4$ 2447.66;
FT-IR (thin film): ν_{max} /cm^{-1} 2960, 2920, 2873.

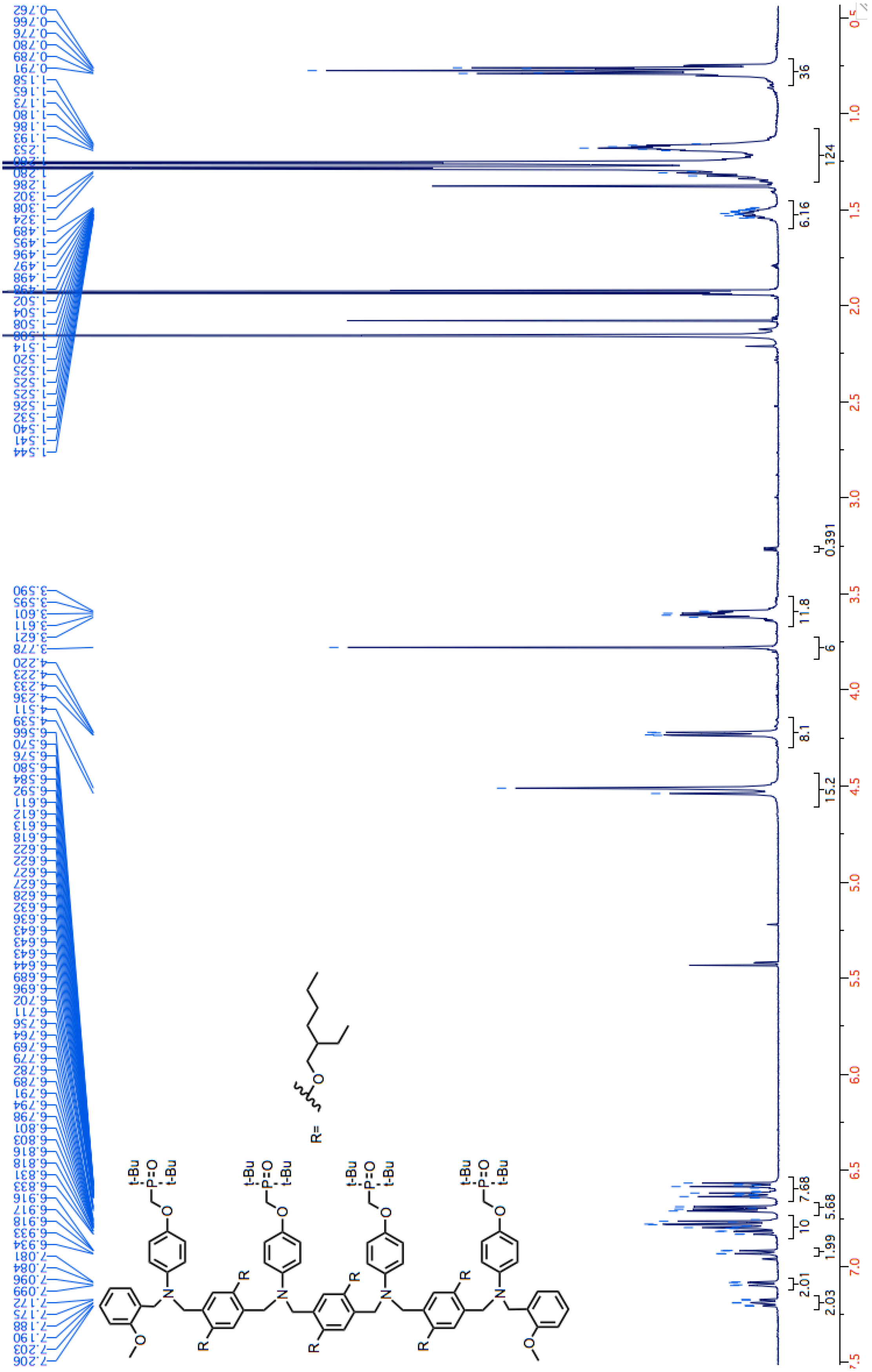
0.5
1.0
1.5
2.0
2.5
3.0
3.5
4.0
4.5
5.0
5.5
6.0
6.5
7.0
36
124
6.16
0.391
11.8
6
8.1
15.2
7.68
5.68
10
1.99
2.01
2.03
t-Bu
P=O
R
N
O
R=

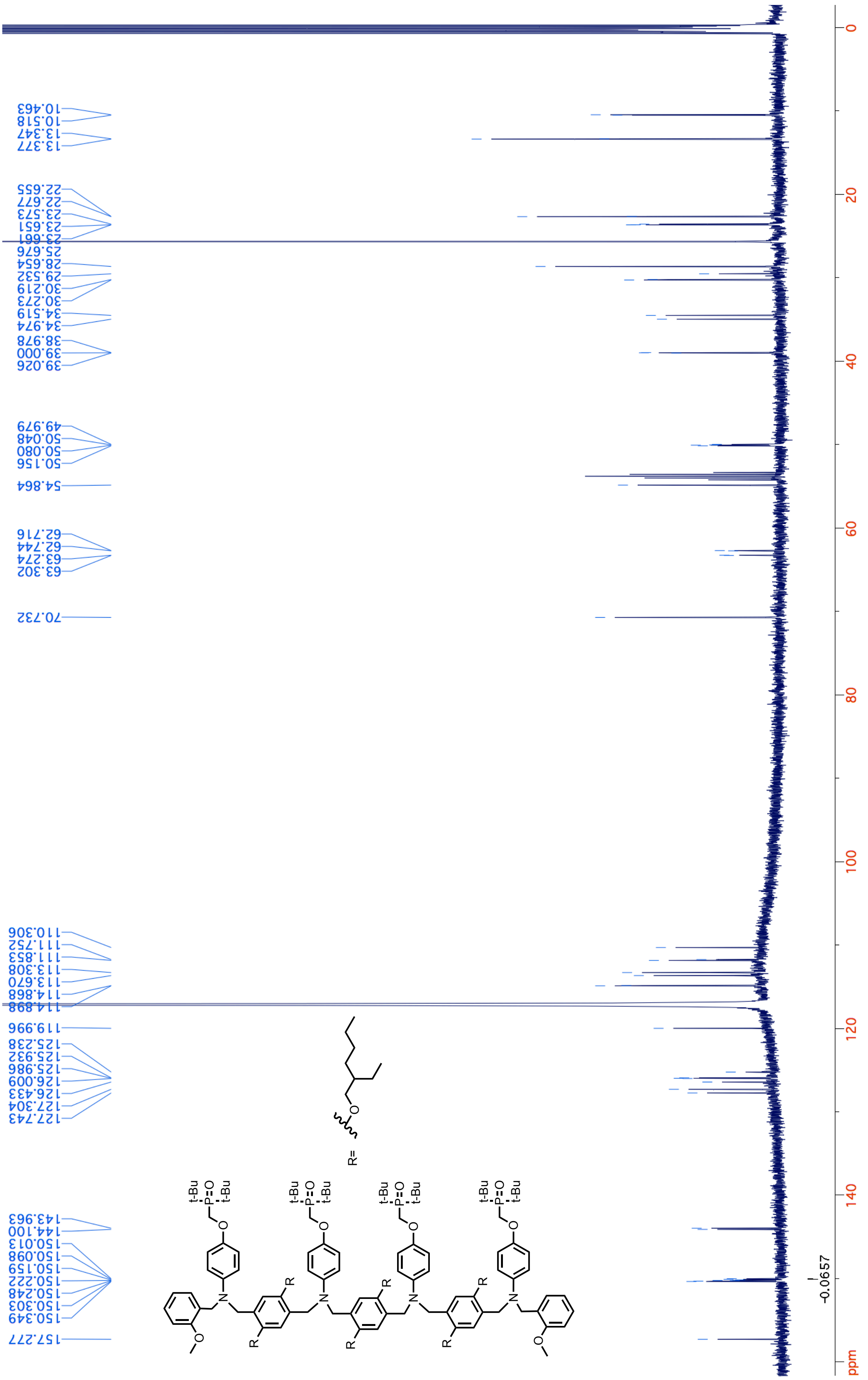
10.463
10.518
13.347
13.377
22.655
22.677
23.573
23.651
23.661
25.676
28.654
29.532
30.219
30.273
34.519
34.974
38.978
39.000
39.026
49.979
50.048
50.080
50.156
54.864
62.716
62.744
63.274
63.302
70.732
110.306
111.752
111.853
113.308
113.670
114.868
114.898
119.996
125.238
125.932
125.986
126.009
126.433
127.304
127.743
143.963
144.100
150.013
150.098
150.159
150.222
150.248
150.303
150.349
157.277
-0.0657
ppm
R=
t-Bu
P=O

Synthesis of 9 [1]

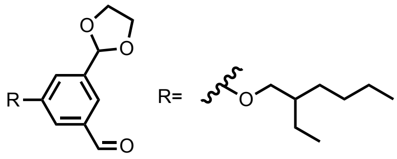

A mixture of **8** (3.8 g, 14 mmol), ethane-1,2-diol (0.08 ml, 14 mmol) and a catalytic amount of p-toluenesulfonic acid in toluene (80 ml) was refluxed for 12 h under nitrogen. After cooling to room temperature, the solution was washed with water (3 x 100 mL) and brine (1 x 100 ml), dried with $MgSO_4$, and the solvent was removed under reduced pressure. The crude product was then purified by column chromatography on silica eluting with hexane /DCM from 0 to 20% DCM. The product was isolated as a yellow oil (1.87 g, 44%).

^{1}H NMR (400 MHz, CD_3CN): δ 9.95 (s, 1H), 7.54 (s, 1H), 7.39 (s, 1H), 7.28 (s, 1H), 5.78 (s, 1H), 4.10-3.94 (m, 6H), 1.75-1.69 (m, 1H), 1.51-1.29 (m, 8H), 0.94-0.86 (m, 6H);
^{13}C NMR (100.6 MHz, CD_3CN): δ 192.1, 160.0, 141.4, 138.2, 120.3, 119.1, 114.2, 102.5, 70.9, 65.3, 39.2, 30.2, 28.8, 23.6, 22.8, 13.4, 10.4;
MS (ES+): m/z (%) = 307.2 (30) [$M+H^+$], 348.2 (100) [$M+H^+$ CH_3CN];
HRMS (ES+): calcd for $C_{18}H_{27}O_4$ 307.1909 found 307.1906;
FT-IR (thin film): ν_{max} /cm^{-1} 3054, 2962, 2931, 2875, 1699, 1597.

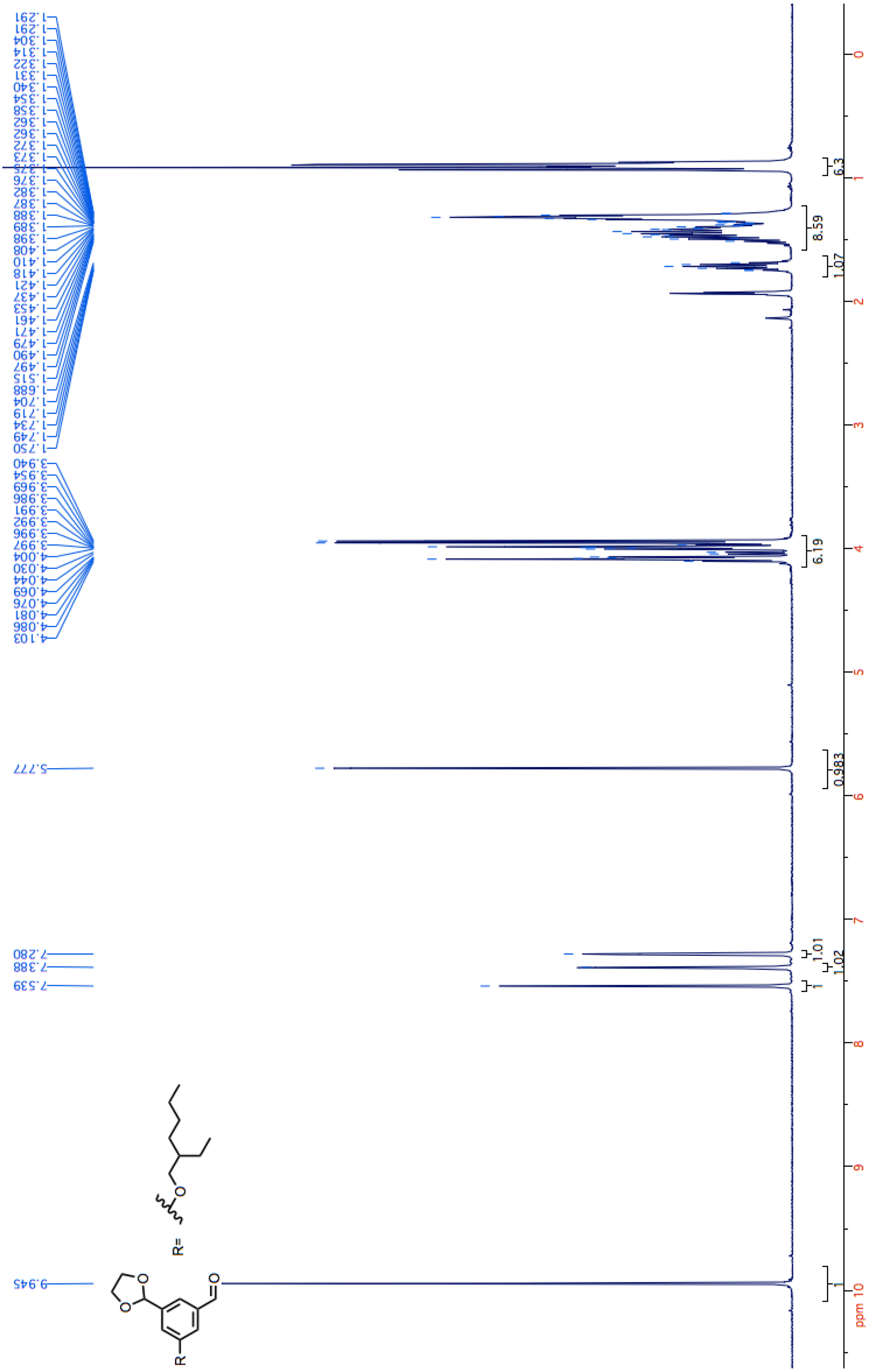
1.291
1.291
1.304
1.314
1.322
1.331
1.340
1.354
1.358
1.362
1.362
1.372
1.373
1.375
1.376
1.382
1.387
1.388
1.389
1.398
1.408
1.410
1.418
1.421
1.437
1.453
1.461
1.471
1.479
1.490
1.497
1.515
1.688
1.704
1.719
1.734
1.749
1.750
3.940
3.954
3.969
3.986
3.991
3.992
3.996
3.997
4.004
4.030
4.044
4.069
4.076
4.081
4.086
4.103
5.777
7.280
7.388
7.539
9.945
R=
6.3
8.59
1.07
6.19
0.983
1.01
1.02
1
1
ppm

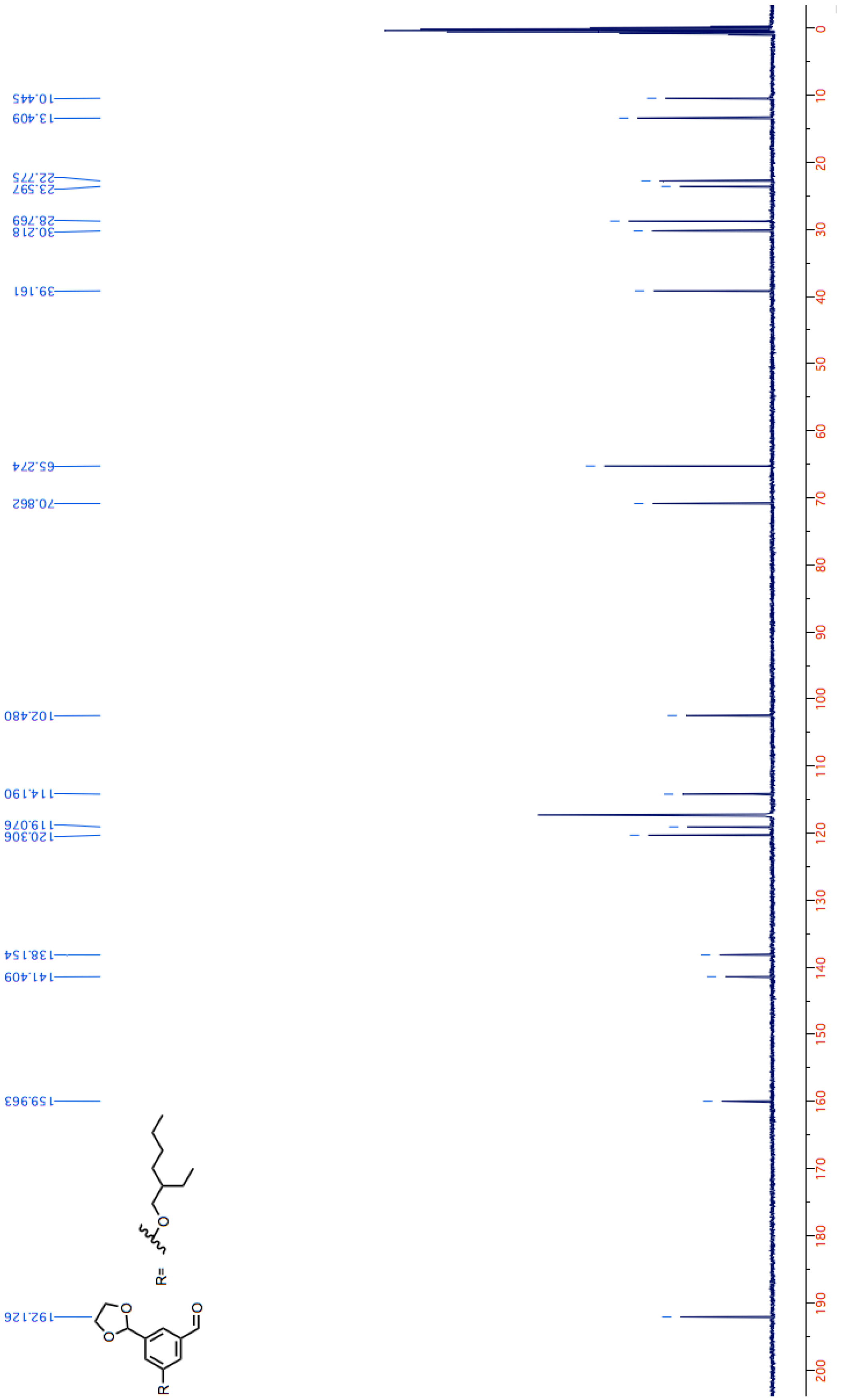
10.445
13.409
22.775
23.597
28.769
30.218
39.161
65.274
70.862
102.480
114.190
119.076
120.306
138.154
141.409
159.963
192.126
R=
0
10
20
30
40
50
60
70
80
90
100
110
120
130
140
150
160
170
180
190
200

Synthesis of 10a

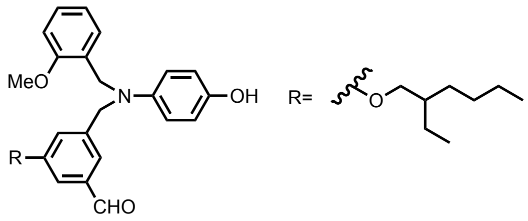

A mixture of **9** (1.0 g, 3.2 mmol), **1a** (0.37 g, 1.6 mmol) and $NaBH(AcO)_3$ (0.95 g, 4.5 mmol) in DCE (6 ml) dried with molecular sieves, dried with molecular sieves, was stirred under nitrogen at room temperature for 2 h. The solution was then washed with saturated aqueous $NaHCO_3$ (1 x 10 ml), water (1 x 10 ml) and brine (1 x 10 ml) and the solvent was removed under reduced pressure. The crude product was then diluted with DCM (20ml) and left stirring with HCl 4M (20ml) for 12 h at room temperature. The solution was then washed with saturated acqueous $NaHCO_3$ (1 x 20 ml), water (1 x 20 ml) and brine (1 x 20 ml). The solution was dried with $MgSO_4$ and the solvent was removed under reduced pressure. The crude material was purified by column chromatography on silica eluting with hexane /EtOAc (85:15). The product was isolated as a yellow oil (0.50 g, 65%).

^{1}H NMR (400 MHz, CD_3CN): δ 9.84 (s, 1H), 7.33 (s, 1H), 7.23-7.08 (m, 4H), 6.93-6.91 (m, 1H), 6.84 (t, J = 7, 1H), 6.62-6.55 (m, 4H), 6.28 (s, 1H), 4.54 (s, 4H), 3.85 (d, J = 6, 2H), 3.78 (s, 3H), 1.70-1.64 (m, 1H), 1.48-1.27 (m, 8H), 0.91-0.87 (m, 6H).

^{13}C NMR (100.6 MHz, CD_3CN): δ 192.4, 160.1, 157.5, 148.6, 143.0, 142.5, 138.2, 128.03, 127.9, 126.6, 120.8, 120.2, 119.8, 115.8, 114.6, 112.0, 110.5, 70.7, 55.0, 54.9, 50.5, 39.1, 30.3, 28.8, 23.6, 22.8, 13.5, 10.5;

MS (ES+): m/z (%) = 476.3 (100) [$M+H^+$];

HRMS (ES+): calcd for $C_{30}H_{38}NO_4$ 476.2801 found 476.2786;

FT-IR (thin film): ν_{max} /cm^{-1} 3386, 2929, 2872, 2958, 1695, 1593, 1514.

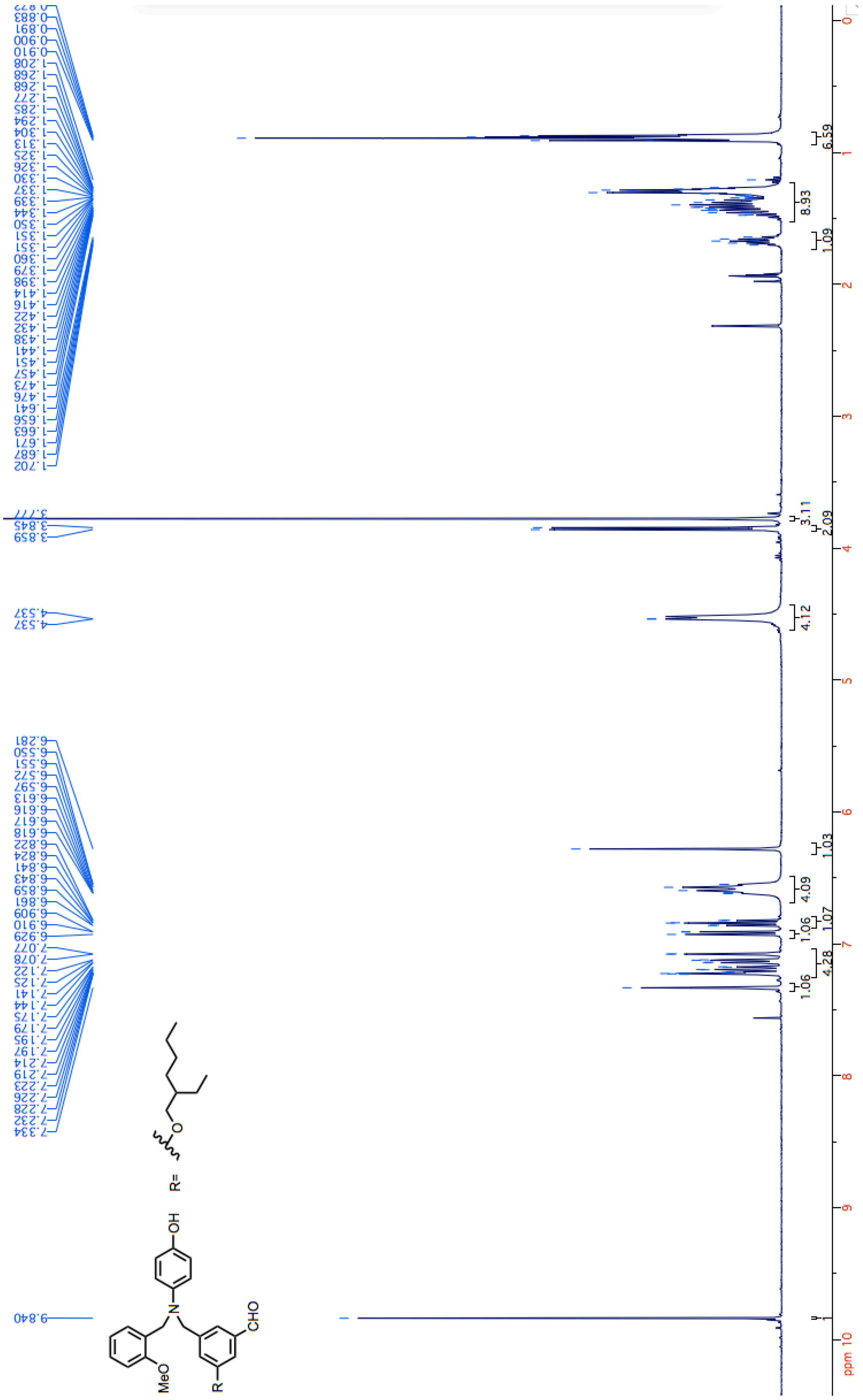
0.883
0.891
0.900
0.910
1.208
1.268
1.268
1.277
1.285
1.294
1.304
1.313
1.325
1.326
1.330
1.337
1.339
1.344
1.350
1.351
1.351
1.360
1.379
1.398
1.414
1.416
1.422
1.432
1.438
1.441
1.451
1.457
1.473
1.476
1.641
1.656
1.663
1.671
1.687
1.702
3.777
3.845
3.859
4.537
4.537
6.281
6.550
6.551
6.572
6.597
6.613
6.616
6.617
6.618
6.822
6.824
6.841
6.843
6.859
6.861
6.909
6.910
6.929
7.077
7.078
7.122
7.125
7.141
7.144
7.175
7.179
7.195
7.197
7.214
7.219
7.223
7.226
7.228
7.232
7.334
9.840
MeO
N
OH
CHO
R
R=
O
6.59
8.93
1.09
3.11
2.09
4.12
1.03
4.09
1.06
1.07
4.28
1.06
1
ppm
0
1
2
3
4
5
6
7
8
9
10

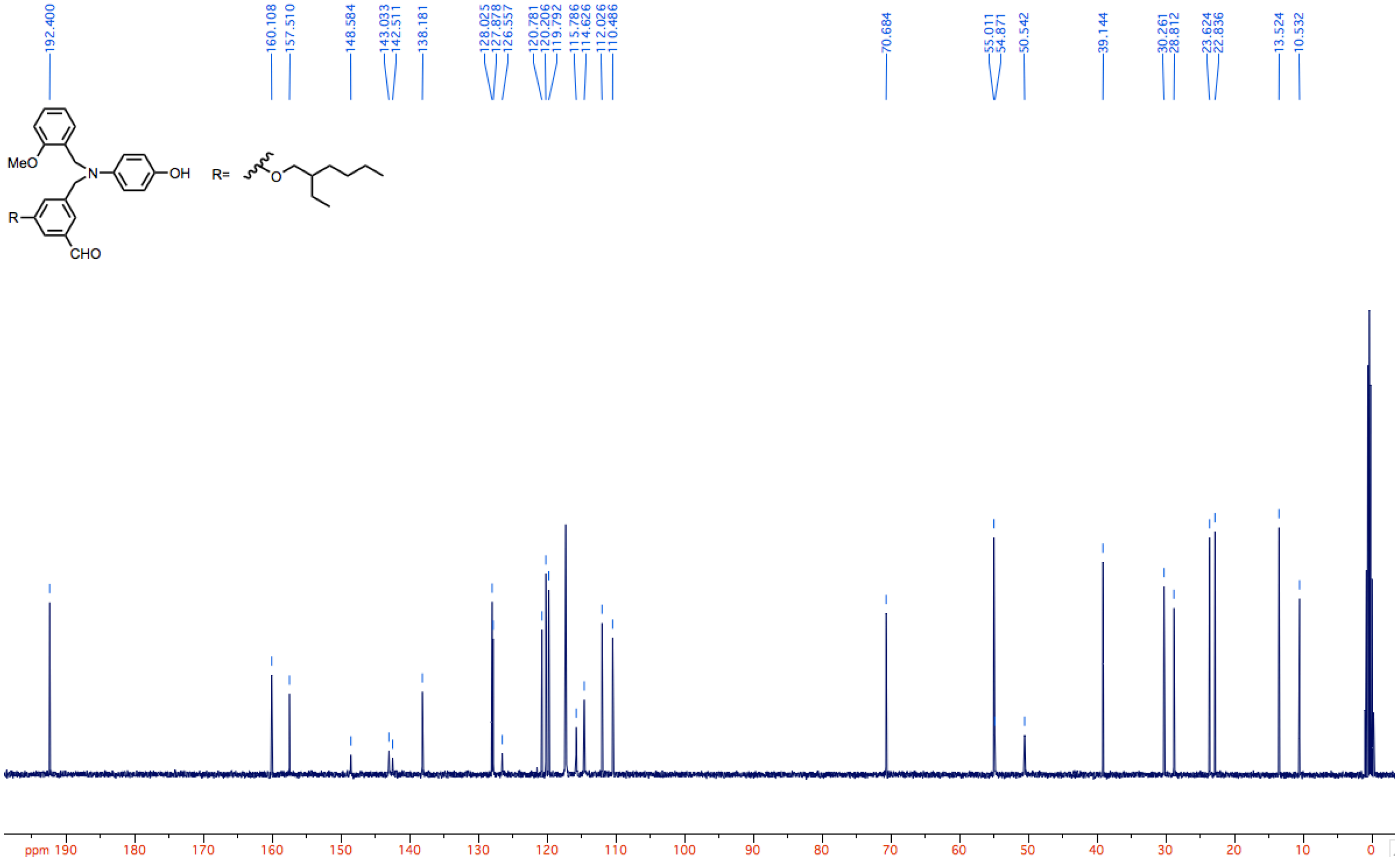

192.400
160.108
157.510
148.584
143.033
142.511
138.181
128.025
127.878
126.557
120.781
120.206
119.792
115.786
114.626
112.026
110.486
70.684
55.011
54.871
50.542
39.144
30.261
28.812
23.624
22.836
13.524
10.532
MeO
N
OH
R
CHO
R=
O
ppm 190
180
170
160
150
140
130
120
110
100
90
80
70
60
50
40
30
20
10
0

Synthesis of 10b

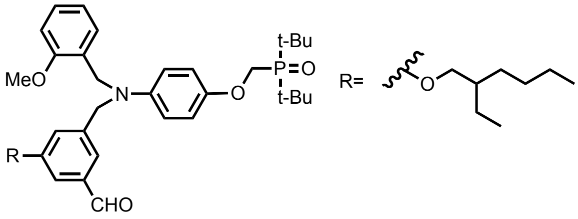

A mixture of **9** (0.80 g, 2.6 mmol), **1b** (0.45 g, 1.1 mmol) and NaBH(AcO)$_3$ (0.66 g, 3.1 mmol) in DCE (5 ml) dried with molecular sieves, was stirred under nitrogen at room temperature for 12 h. The solution was then washed with saturated aqueous $NaHCO_3$ (1 x 10 ml), water (1 x 10 ml) and brine (1 x 10 ml) and the solvent was removed under reduced pressure. The crude product was then diluted with DCM (10ml) and left stirring with HCl 4M (10ml) for 12 h at room temperature. The solution was then washed with saturated aqueous $NaHCO_3$ (1 x 20 ml), water (1 x 20 ml) and brine (1 x 20 ml), dried with $MgSO_4$, and the solvent was removed under reduced pressure. The crude material was purified by column chromatograph on silica eluting with $CHCl_3$/MeOH (99:1). The product was isolated as a orange oil (0.51 g, 71%).

^{1}H NMR (500 MHz, CD$_3$CN): δ 9.86 (s, 1H), 7.34 (s, 1H), 7.24-7.20 (m, 2H), 7.11 (d, J = 7, 1H), 7.06 (s, 1H), 6.95 (d, J = 8, 1H), 6.86-6.83 (m, 1H), 6.79 (d, J = 9, 2H), 6.61 (d, J = 9, 2H), 4.62 (s, 2H), 4.58 (s, 2H), 4.24 (d, J = 6, 2H), 3.87 (d, J = 6, 2H), 3.80 (s, 3H), 1.69-1.64 (m, 1H), 1.48-1.25 (m, 26 H), 0.90-0.86 (m, 6H);
^{31}P NMR (202.4 MHz, CD$_3$CN): δ 55.29;
^{13}C NMR (126 MHz, CD$_3$CN): δ 193.4, 161.1, 158.5, 151.5, 151.5, 144.6, 143.8, 139.2, 129.1, 128.6, 127.3, 121.4, 121.2, 120.6, 116.1, 114.8, 113.2, 111.5, 71.7, 64.3, 63.8, 56.0, 55.6, 51.4, 40.1, 36.1, 35.6, 31.2, 29.7, 26.8, 24.6, 23.8, 14.4, 11.4;
MS (ES+): m/z (%) = 650.4 (100) [M+H$^+$];
HRMS (ES+): calcd for $C_{39}H_{57}NO_5P$ 650.3974 found 650.3993.
FT-IR (thin film): ν_{max} /cm^{-1} 2958, 2929, 2871, 1698, 1593, 1512.

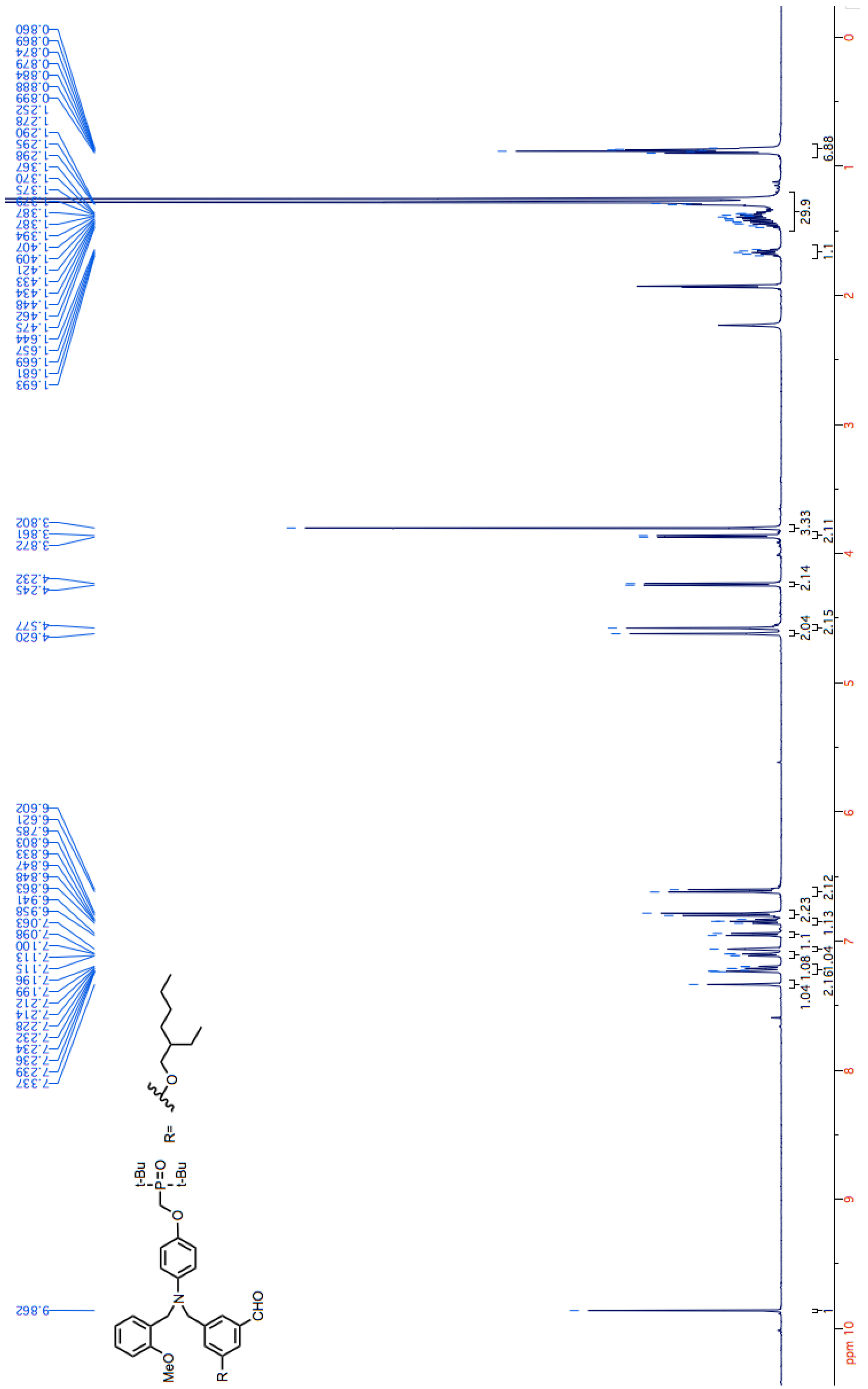

0.860
0.869
0.874
0.879
0.884
0.888
0.899
1.252
1.278
1.290
1.295
1.298
1.367
1.370
1.375
1.382
1.387
1.394
1.407
1.409
1.421
1.433
1.434
1.448
1.462
1.475
1.644
1.657
1.669
1.681
1.693
3.802
3.861
3.872
4.232
4.245
4.577
4.620
6.602
6.621
6.785
6.803
6.833
6.847
6.848
6.863
6.941
6.958
7.063
7.098
7.100
7.113
7.115
7.196
7.199
7.212
7.214
7.228
7.232
7.234
7.236
7.239
7.337
9.862
MeO
N
O
t-Bu
P=O
t-Bu
CHO
R
R=
O
6.88
29.9
1.1
3.33
2.11
2.14
2.04
2.15
2.12
2.23
1.13
1.1
1.04
1.08
2.16
1.04
1
ppm

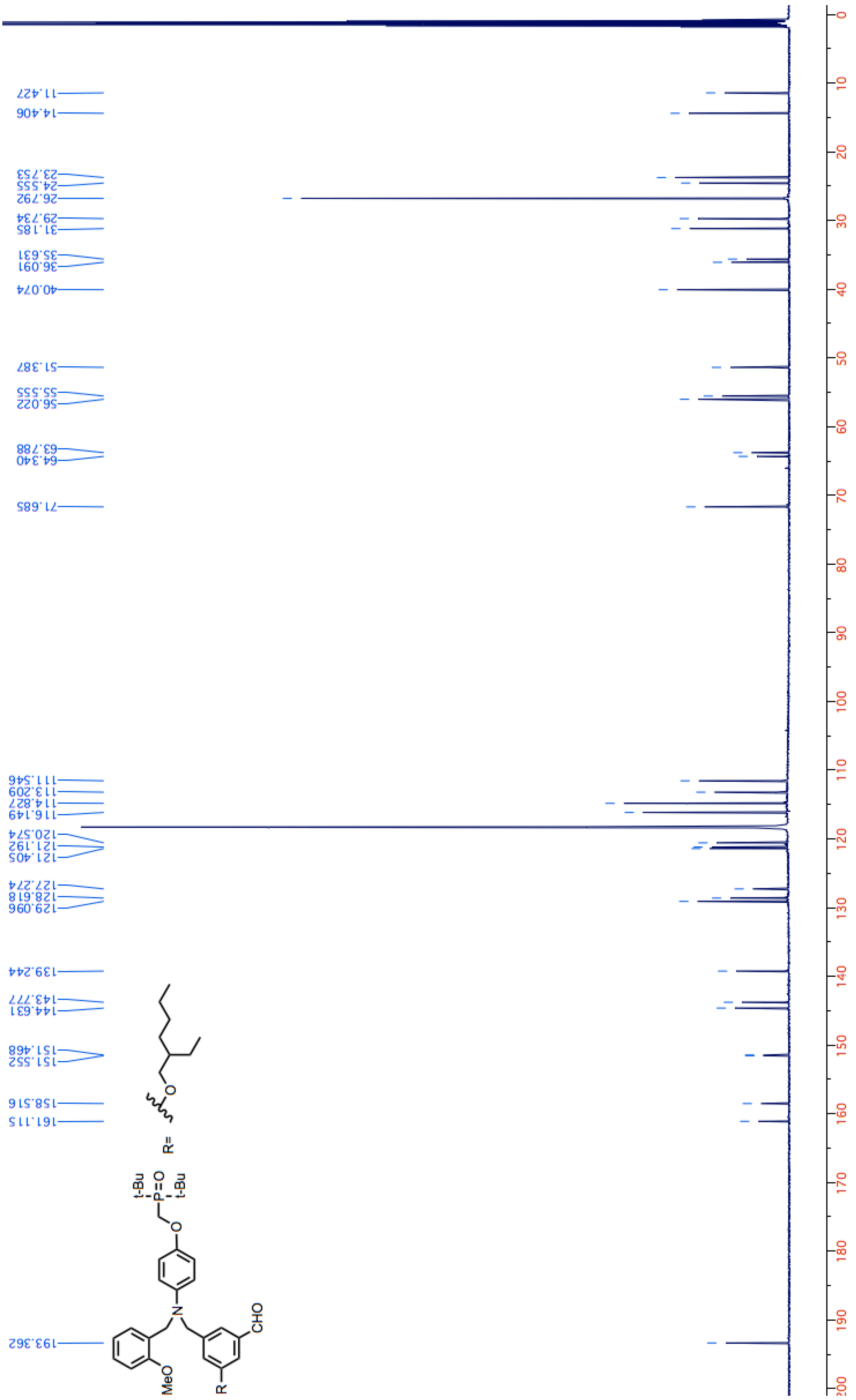
11.427
14.406
23.753
24.555
26.792
29.734
31.185
35.631
36.091
40.074
51.387
55.555
56.022
63.788
64.340
71.685
111.546
113.209
114.827
116.149
120.574
121.192
121.405
127.274
128.618
129.096
139.244
143.777
144.631
151.468
151.552
158.516
161.115
193.362
t-Bu
P=O
t-Bu
R=
MeO
N
CHO
R
0
10
20
30
40
50
60
70
80
90
100
110
120
130
140
150
160
170
180
190
200

Synthesis of 11a

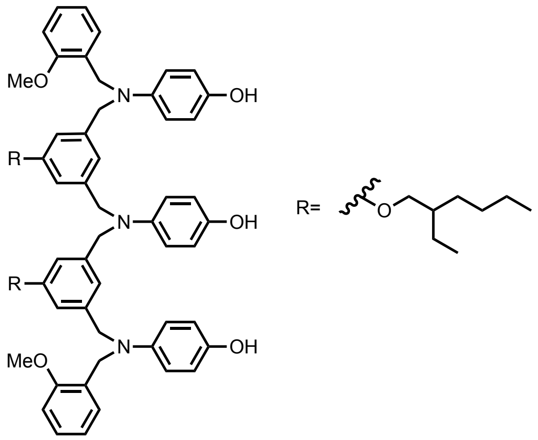

A mixture of **10a** (0.17 g, 0.37 mmol), of *para*-aminophenol (0.01 g, 0.092 mmol) and $NaBH(AcO)_3$ (0.11 mg, 0.51 mmol), AcOH (4 eq) in DCE (300 μ l) dried with molecular sieves, was stirred under nitrogen at room temperature for 12 h. The solution was then washed with saturated aqueous $NaHCO_3$ (1 x 10 ml), water (1 x 10 ml) and brine (1x 10 ml) dried with $MgSO_4$ and the solvent was removed under reduced pressure. The crude product was then purified by column chromatograph on silica eluting with hexane /EtOAc (70:30). The product was isolated as a red oil (10 mg, 11%).

^{1}H NMR (500 MHz, CD_3CN): δ 7.19-7.16 (td, J = 8, J = 1, 2H), 7.10-7.09 (m, 2H), 6.91 (d, J = 8 , 2H), 6.81 (td, J = 7, J = 1, 2H), 6.71 (s, 2H), 6.62 (s, 2H), 6.59 (s, 2H), 6.57-6.49 (m, 12H), 4.42 (s, 8H), 4.29 (s, 4H), 3.77 (s, 6H), 3.70 (d, J = 6, 4H), 1.60-1.56 (m, 2H), 1.42-1.22 (m, 16H), 0.87-0.83 (m, 12H);

^{13}C NMR (126.7 MHz, CD_3CN): δ 160.8, 158.5, 149.9, 149.4, 143.8, 142.6, 142.4, 128.9, 128.9, 127.9, 121.2, 119.2, 117.1, 116.6, 116.5, 115.7, 112.7, 112.5, 111.5, 71.3, 56.7, 56.5, 56.0, 51.4, 40.2, 31.3, 29.8, 24.6, 23.8, 14.5, 11.5;

MS (ES+): m/z (%) = 1028.6 (100) [$M+H^+$];

HRMS (ES+): calcd for $C_{66}H_{82}N_3O_7$ 1028.6153 found 1028.6177;

FT-IR (thin film): ν_{max} /cm^{-1} 3347, 2956, 2923, 2853, 1596, 1513.

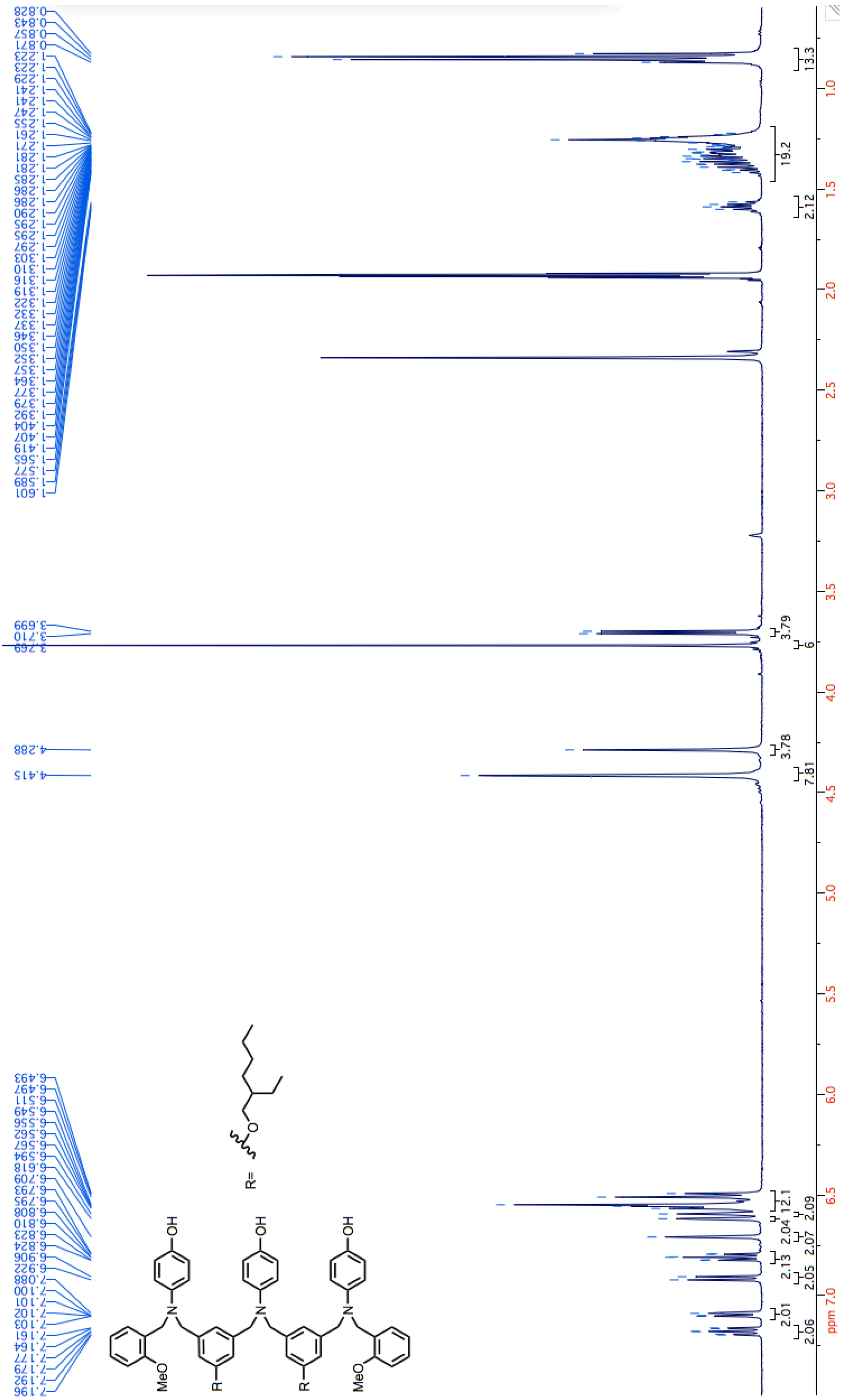

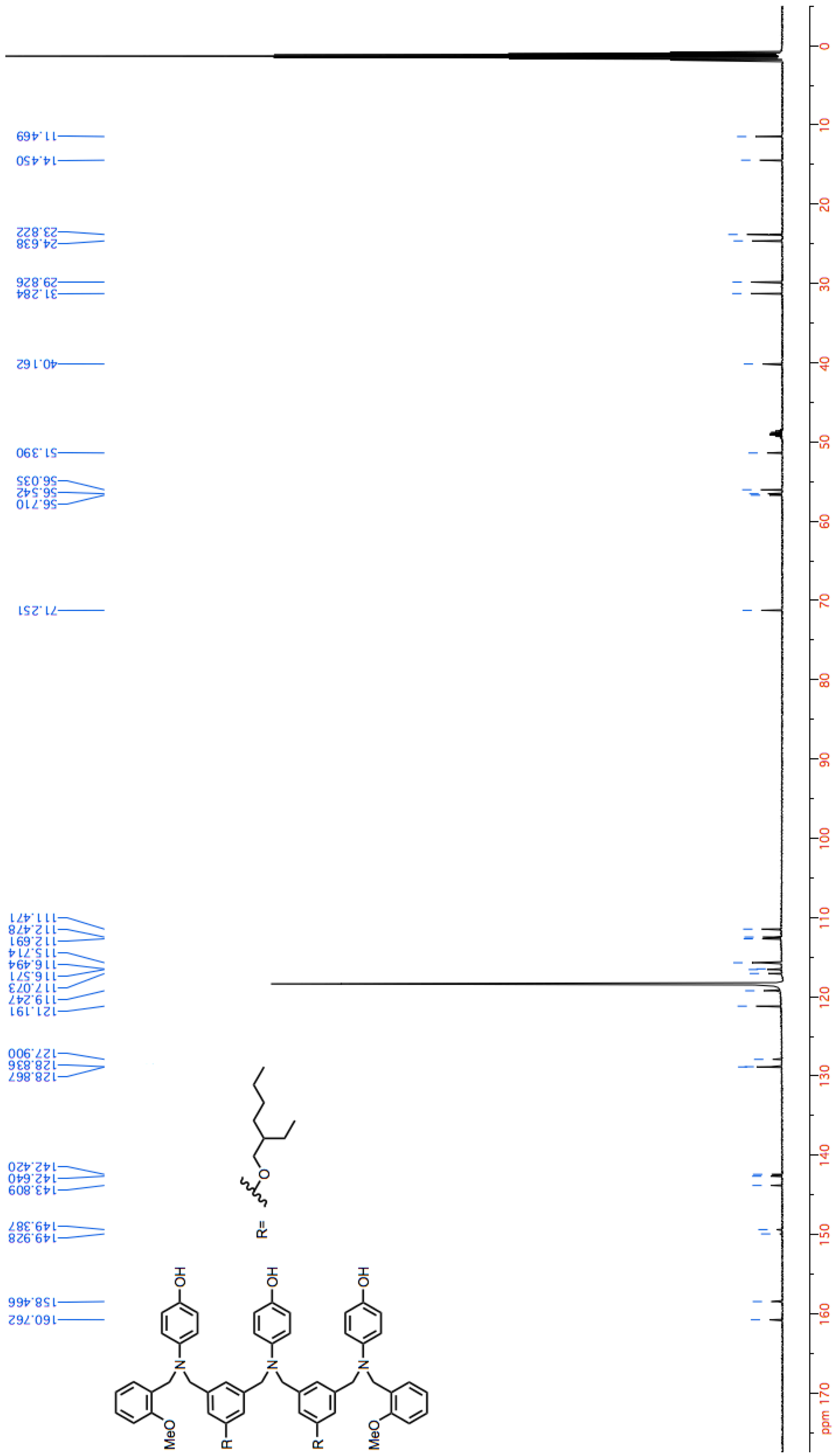
11.469
14.450
23.822
24.638
29.826
31.284
40.162
51.390
56.035
56.542
56.710
71.251
111.471
112.478
112.691
115.714
116.494
116.571
117.073
119.247
121.191
127.900
128.836
128.867
142.420
142.640
143.809
149.387
149.928
158.466
160.762
OH
N
MeO
R
R=
O
ppm

Synthesis of 11b

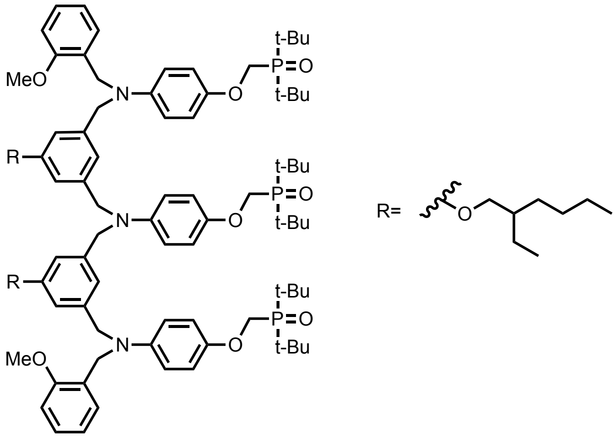

A mixture of **10b** (195 mg, 0.31 mmol), of di-*tert*-butyl((4-((2-methoxybenzyl)amino)phenoxy)methyl)phosphine oxide (21.0 mg, 0.07 mmol), NaBH(AcO)$_3$ (90.0 mg, 0.42 mmol) and AcOH (4 eq) in DCE (300 μ l) dried with molecular sieves, was stirred under nitrogen at room temperature for 12 h. The solution was then washed with saturated aqueous NaHCO$_3$ (1 x 10 ml), water (1 x 10 ml) and brine (1 x 10 ml), dried with MgSO$_4$ and the solvent was removed under reduced pressure. The crude product was then purified by column chromatograph on silica eluting with EtOAc/MeOH (90:10). The product was isolated as an orange oil (91 mg, 83%).

^{1}H NMR (500 MHz, CD$_3$CN): δ 7.17-7.14 (m, 2H), 7.02 (d, J = 7, 2H), 6.89 (d, J = 8.0, 2H), 6.77 (t, J = 7, 2H), 6.73-6.70 (m, 6H), 6.64 (s, 2H), 6.60 (s, 2H), 6.56-6.50 (m, 8H), 4.43 (s, 4H), 4.41 (s, 4H), 4.29 (s, 4H), 4.22-4.19 (m, 6H), 3.73 (s, 6H), 3.67 (d, J = 6, 4H), 1.57-1.52 (m, 2H), 1.35-1.19 (m, 70H), 0.84-0.78 (m, 12H);

^{31}P NMR (202.4 MHz, CD$_3$CN): δ 55.43, 55.41;

^{13}C NMR (125.7 MHz, CD$_3$CN): δ 160.8, 158.4, 151.68, 151.6, 151.3, 151.2, 145.0, 145.0, 142.3, 142.1, 128.9, 128.4, 127.3, 121.2, 118.6, 116.1, 116.0, 115.7, 114.7, 112.5, 112.2, 111.4, 71.2, 64.4, 63.8, 56.1, 56.1, 55.9, 51.2, 40.1, 36.1, 35.6, 31.2, 29.8, 26.8, 23.8, 14.5, 11.5;

MS (ES+): m/z (%) = 1550.9 (100) [M+H$^+$];

HRMS (ES+): calcd for $C_{93}H_{139}N_3O_{10}P_3$ 1550.9673 found 1550.9702.

FT-IR (thin film): ν_{max} /cm^{-1} 2957, 2924, 2855, 1596.

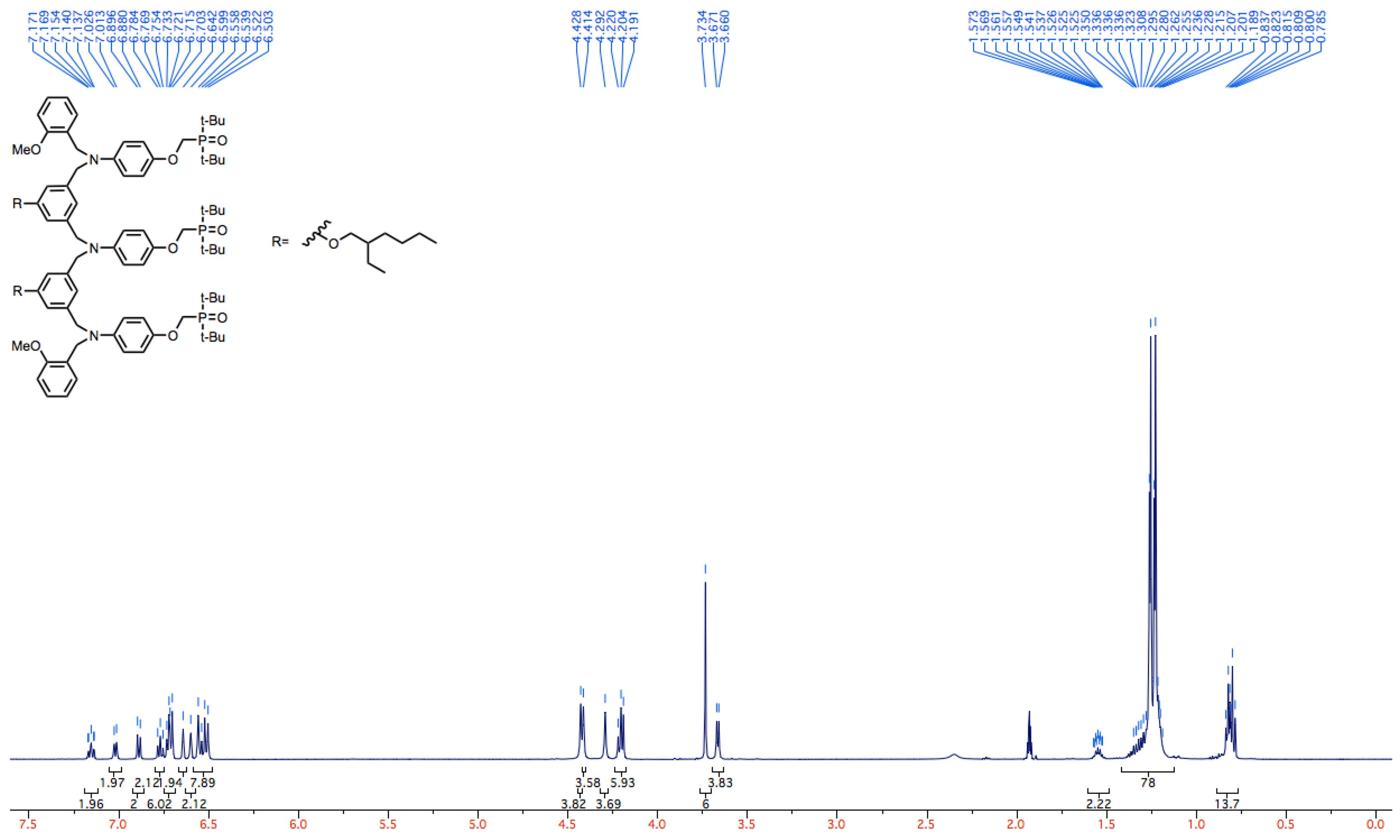
7.171 7.169 7.154 7.140 7.137 7.026 7.013 6.896 6.880 6.784 6.769 6.754 6.733 6.721 6.715 6.703 6.642 6.599 6.558 6.539 6.522 6.503
4.428 4.414 4.292 4.220 4.204 4.191
3.734 3.671 3.660
1.573 1.569 1.561 1.557 1.549 1.541 1.537 1.526 1.525 1.525 1.350 1.336 1.336 1.336 1.323 1.308 1.295 1.280 1.262 1.255 1.236 1.228 1.215 1.207 1.201 1.189 0.837 0.823 0.815 0.809 0.800 0.785
MeO
N
O
t-Bu
P=O
t-Bu
R
R=
O
1.97
2.12
1.94
7.89
1.96
2
6.02
2.12
3.58
5.93
3.82
3.69
3.83
6
78
2.22
13.7
7.5 7.0 6.5 6.0 5.5 5.0 4.5 4.0 3.5 3.0 2.5 2.0 1.5 1.0 0.5 0.0

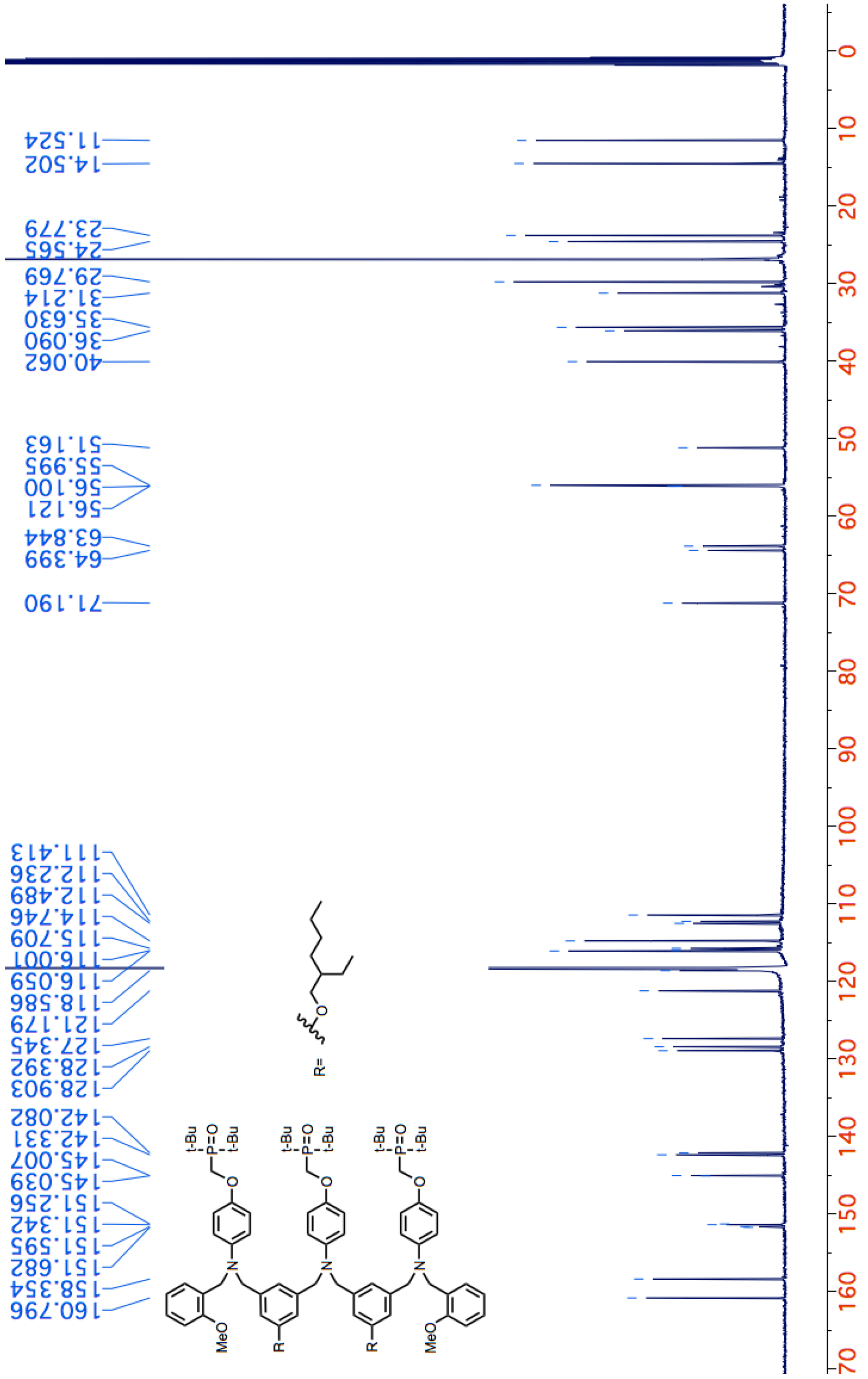
11.524
14.502
23.779
24.565
29.769
31.214
35.630
36.090
40.062
51.163
55.995
56.100
56.121
63.844
64.399
71.190
111.413
112.236
112.489
114.746
115.709
116.001
116.059
118.586
121.179
127.345
128.392
128.903
142.082
142.331
145.007
145.039
151.256
151.342
151.595
151.682
158.354
160.796
R=
MeO
t-Bu

Synthesis of 13a

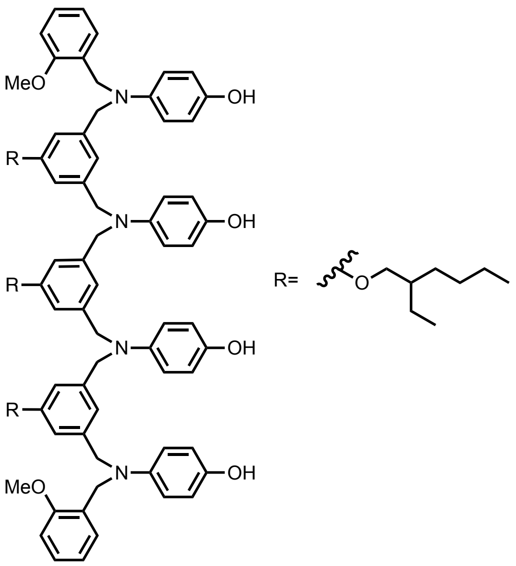

A mixture of **10a** (0.21 g, 0.44 mmol), of **12a** (0.05 g, 0.11 mmol) and $NaBH(AcO)_3$ (0.13 g, 0.61 mmol), AcOH (4 eq) in DCE (500 μl) dried with molecular sieves, was stirred under nitrogen at room temperature for 6h. The solution was then washed with saturated aqueous $NaHCO_3$ (1 x 10 ml), water (1 x 10 ml) and brine (1 x 10 ml), dried with $MgSO_4$ and the solvent was removed under reduced pressure. The crude product was then purified by column chromatograph on silica eluting with hexane /Et_2O (50:50). The product was isolated as a red oil (0.56 g, 37%).

^{1}H NMR (400 MHz, CD_3CN): δ 7.19-7.15 (m, 2H), 7.09-7.07 (m, 2H), 6.91-6.89 (m, 2H), 6.82-6.78 (m, 2H), 6.69 (s, 2H), 6.66 (s, 1H), 6.61-6.47 (m, 22H), 6.301 (s, 2H), 6.236 (s, 2H), 4.40 (s, 8H), 4.27 (s, 8H), 3.75 (s, 6H), 3.70-3.67 (m, 6H), 1.61-1.54 (m, 3H), 1.42-1.19 (m, 24H), 0.86-0.81 (m, 18H);

^{13}C NMR (100.6 MHz, CD_3CN): δ 159.7, 157.4, 148.9, 148.3, 142.8, 142.7, 141.6, 141.3, 141.2, 127.8, 127.7, 126.8, 120.1, 118.6, 118.2, 116.0, 115.5, 115.4, 114.6, 111.7, 111.6, 111.4, 110.4, 70.2, 55.6, 55.5, 55.0, 50.3, 39.1, 30.2, 28.8, 23.6, 22.8, 13.4, 10.4;

MS (ES+): m/z (%) = 1367.8 (100) $[M+H^+]$;

HRMS (ES+): calcd for $C_{88}H_{111}N_4O_9$ 1367.8351 found 1367.8411;

FT-IR (thin film): ν_{max} /cm^{-1} 3342, 2962, 2923, 2854, 1595, 1513.

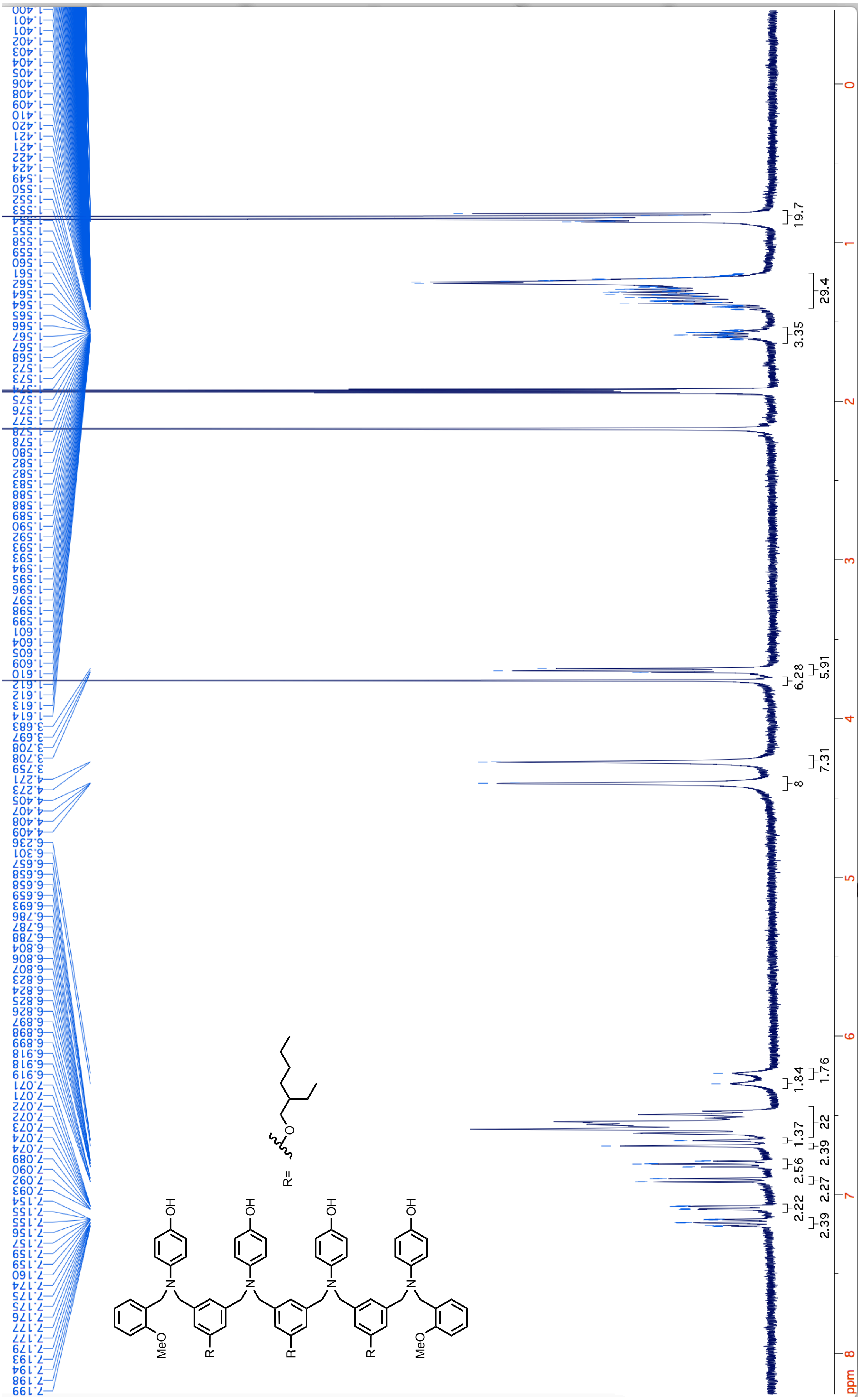

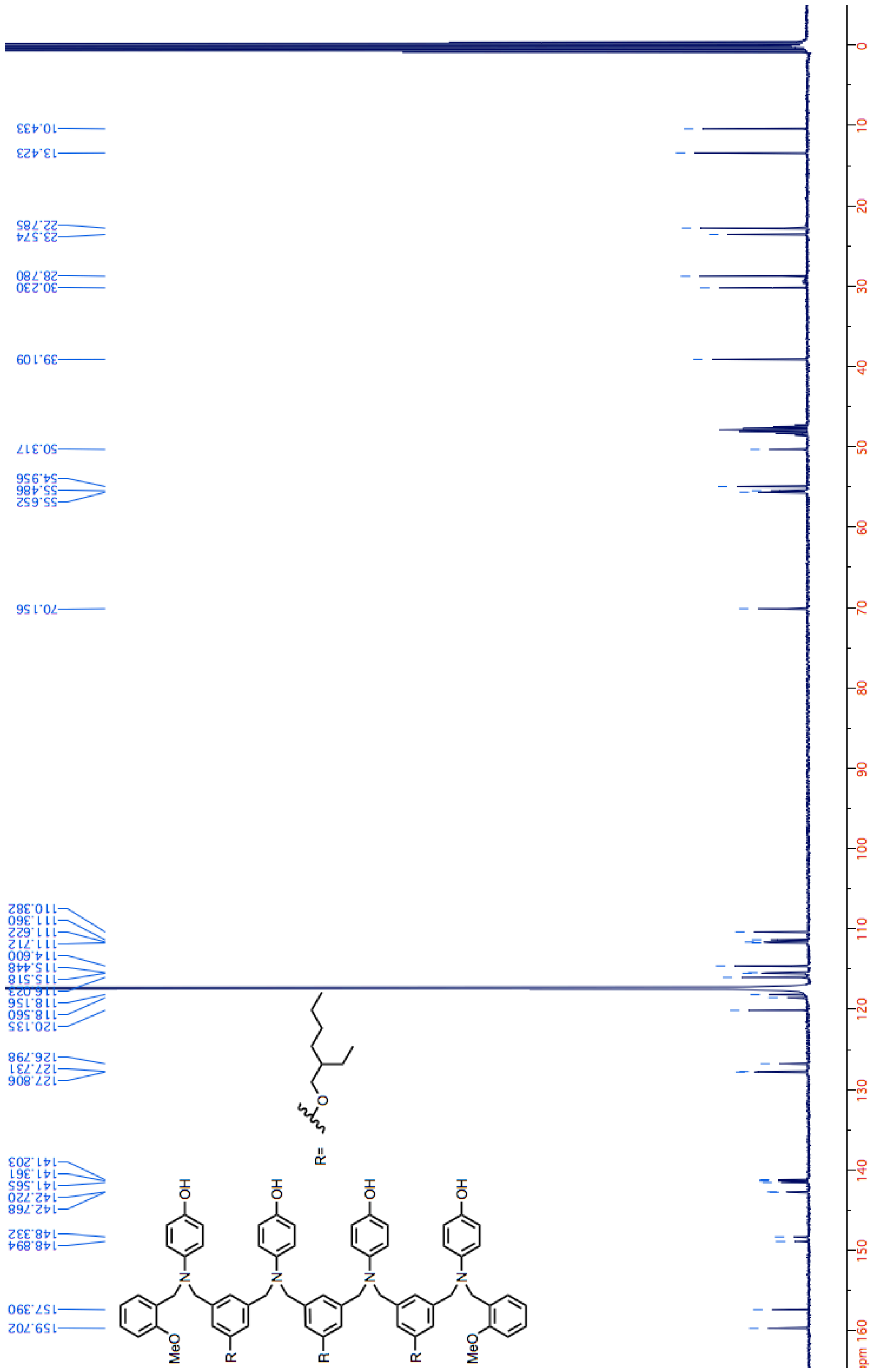

10.433
13.423
22.785
23.574
28.780
30.230
39.109
50.317
54.956
55.486
55.652
70.156
110.382
111.360
111.622
111.712
114.600
115.448
115.518
116.023
118.156
118.560
120.135
126.798
127.731
127.806
141.203
141.361
141.565
142.720
142.768
148.332
148.894
157.390
159.702
OH
N
MeO
R
R=
O
ppm

Synthesis of 13b

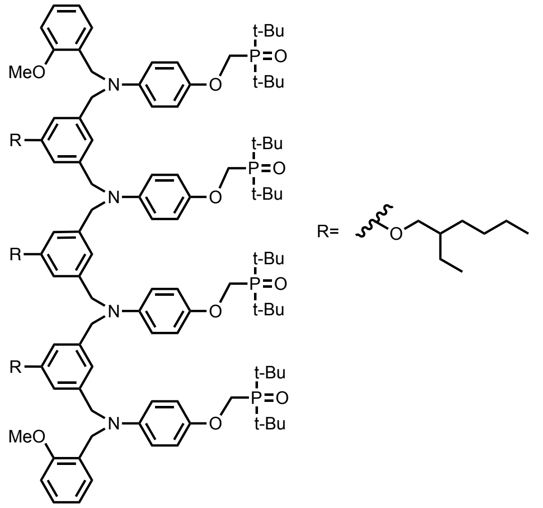

A mixture of **10b** (85 mg, 0.1 mmol), of **12b** (26 mg, 0.03 mmol) and $NaBH(AcO)_3$ (40 mg, 0.2 mmol), AcOH (4 eq) in DCE (100 μl) dried with molecular sieves, was stirred under nitrogen at room temperature for 24 h. The solution was then washed with saturated aqueous $NaHCO_3$ (1 x 10 ml), water (1 x 10 ml) and brine (1 x 10ml), dried with $MgSO_4$ and the solvent was removed under reduced pressure. The crude product was then purified by column chromatograph on silica eluting with EtOAc/MeOH (90:10). The product was isolated as an orange oil (24 mg, 36%)

^{1}H NMR (400 MHz, CD_3CN): δ 7.18-7.14 (m, 2H), 7.02-7.00 (m, 2H), 6.90-6.88 (m, 2H), 6.79-6.69 (m, 10H), 6.63-6.49 (m, 17H), 4.42 (s, 4H), 4.41 (s, 4H), 4.28 (s, 8H), 4.21-4.18 (m, 8H), 3.73 (s, 6H), 3.65 (d, J = 6, 6H), 1.57-1.51 (m, 3H), 1.34-1.17 (m, 96H), 0.84-0.77 (m, 18H);

^{31}P NMR (162.0 MHz, CD_3CN): δ 55.24, 55.22;

^{13}C NMR (100.6 MHz, CD_3CN): δ 159.81, 159.76, 157.4, 150.74, 150.63, 150.36, 150.25, 144.08, 144.00, 141.4, 141.09, 141.01, 127.9, 127.4, 126.3, 120.2, 118.0, 117.6, 115.07, 115.03, 114.7, 113.7, 111.58, 111.52, 111.2, 110.4, 70.2, 63.5, 62.8, 55.14, 55.02, 50.2, 39.1, 35.2, 34.6, 30.2, 28.8, 25.9, 23.6, 22.8, 13.5, 10.5;

MS (ES+): m/z (%) = 2064.3 (100) [M+H$^+$];

HRMS (ES+): calcd for $C_{124}H_{187}N_4O_{13}P_4$ 2064.3045 found 2064.3040;

FT-IR (thin film): ν_{max} /cm^{-1} 2957, 2925, 2871, 1595, 1512.

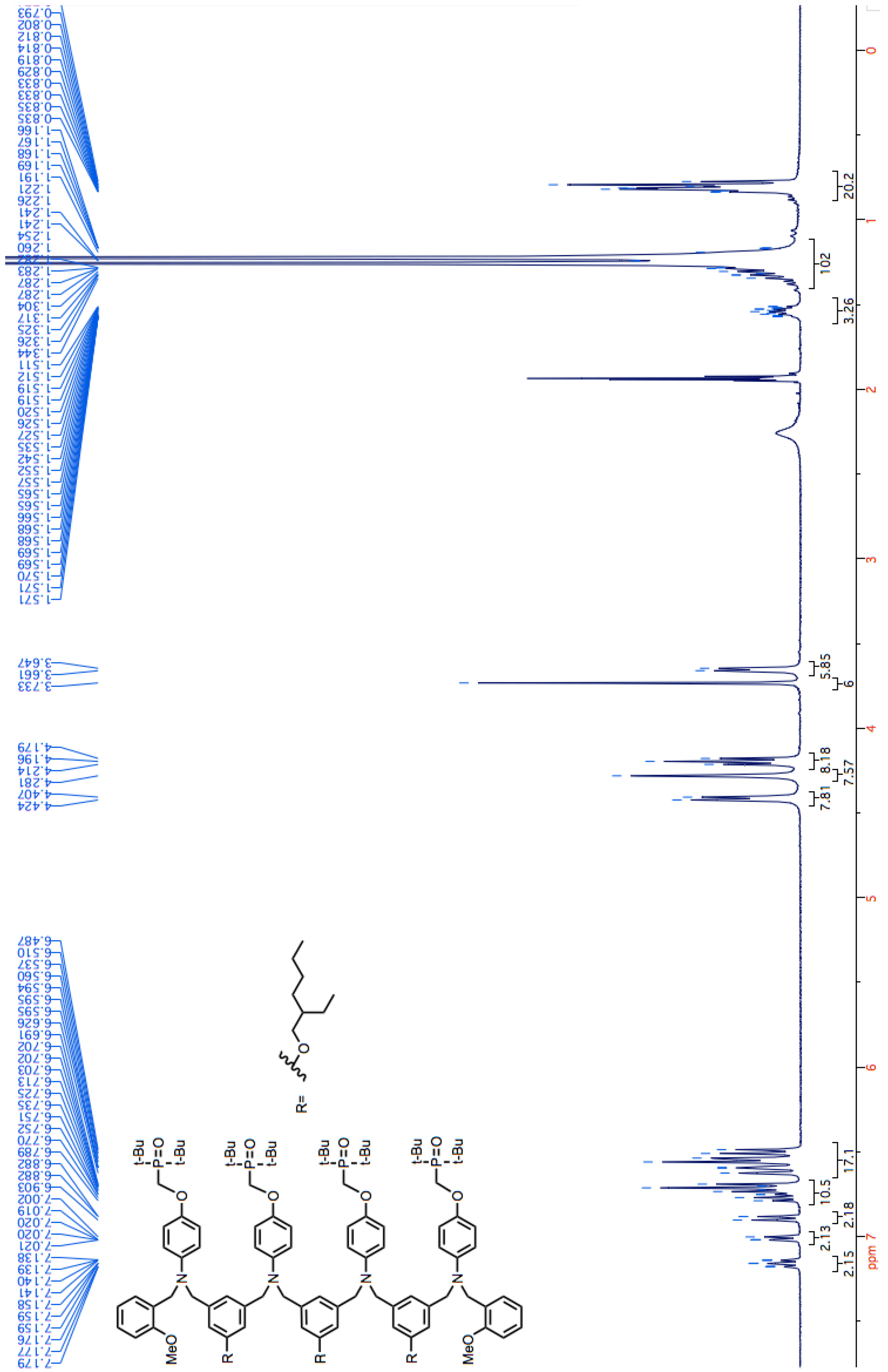
3.647
3.661
3.733
4.179
4.196
4.214
4.281
4.407
4.424
20.2
102
3.26
5.85
6
8.18
7.57
7.81
17.1
10.5
2.18
2.13
2.15
ppm
t-Bu
P=O
MeO
R
N
O
R=

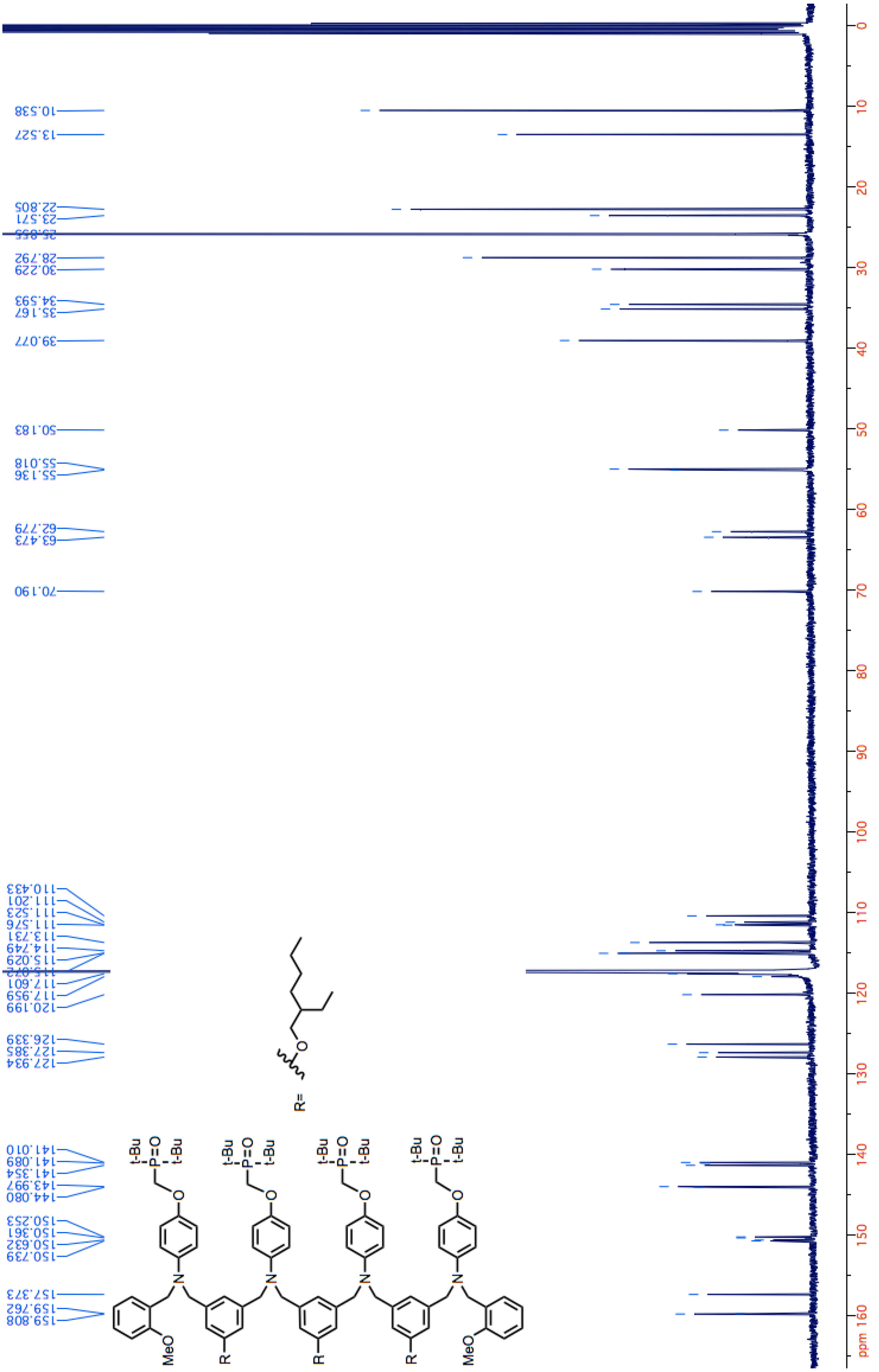
10.538
13.527
22.805
23.571
28.792
30.229
34.593
35.167
39.077
50.183
55.018
55.136
62.779
63.473
70.190
110.433
111.201
111.523
111.576
113.731
114.749
115.029
117.601
117.959
120.199
126.339
127.385
127.934
141.010
141.089
141.354
143.997
144.080
150.253
150.361
150.632
150.739
157.373
159.762
159.808
ppm

Binding studies

All binding constants were measured by means of NMR titrations. A known concentration of host solution (0.5-1 mM) in deuterated toluene or chloroform was prepared. A fraction of the host stock solution (0.45-0.6 ml) was transferred to a NMR tube. The guest solution (1-10 mM) was then prepared by dissolving it in the host stock solution. In this way the concentration of host is maintained constant throughout the titration. ^{31}P NMR spectra were recorded after successive additions of aliquots of guest solution. The observed changes in chemical shift were analysed using a purpose-written fitting program in Microsoft Excel. Errors are quoted as two times the standard deviation.

a)

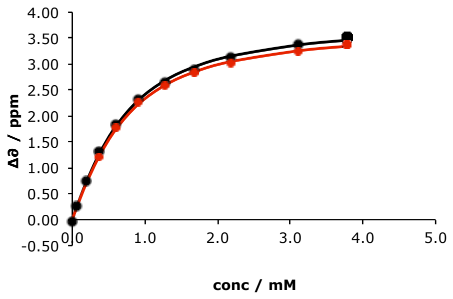

b)

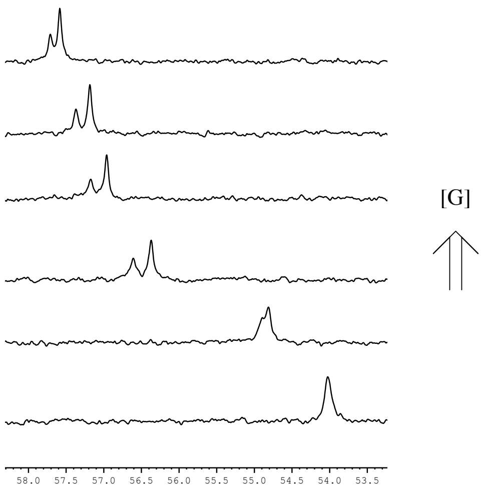

c)

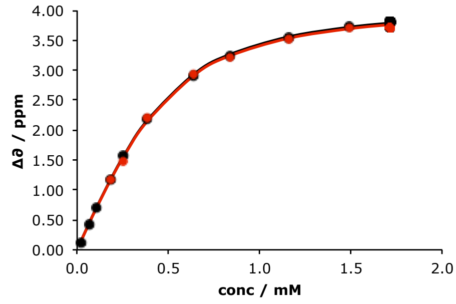

d)

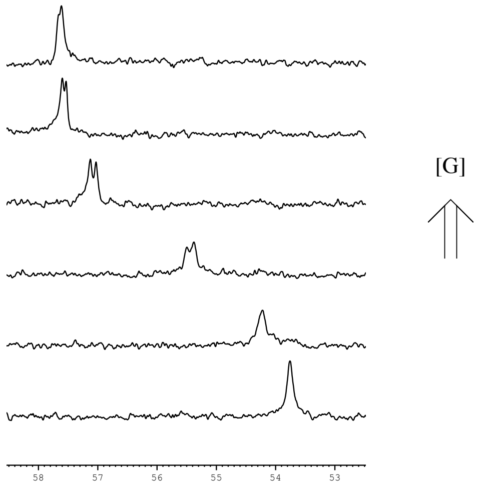

Figure S1 a) ^{31}P NMR chemical shift as a function of guest concentration for addition of 5a to 5b. The line represents the best fit to a 1:1 binding isotherm; b) 162 MHz ^{31}P NMR titration data for addition of 5a to 5b in toluene-*d*8 at 298 K; c) ^{31}P NMR chemical shift as a function

of guest concentration for addition of 7a to 7b. The line represents the best fit to a 1:1 binding isotherm; d) 162 MHz ^{31}P NMR titration data for addition of 7a to 7b in toluene-*d*8 at 298 K.

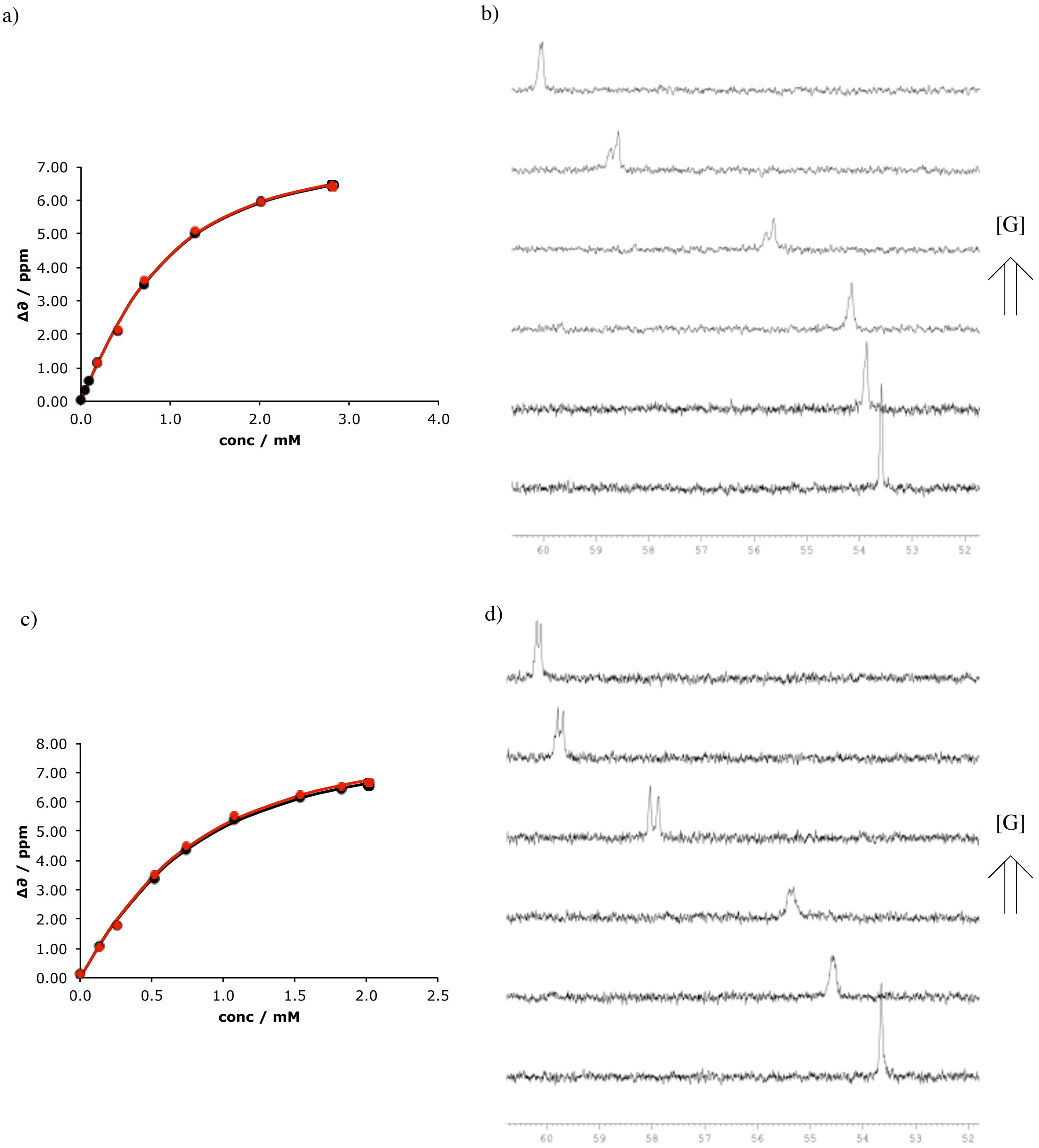

Figure S2 a) ^{31}P NMR chemical shift as a function of guest concentration for addition of 11a to 11b. The line represents the best fit to a 1:1 binding isotherm; b) 162 MHz ^{31}P NMR titration data for addition of 11a to 11b in toluene-*d*8 at 298 K; c) ^{31}P NMR chemical shift as a function of guest concentration for addition of 13a to 13b. The line represents the best fit to a 1:1 binding isotherm; d) 162 MHz ^{31}P NMR titration data for addition of 13a to 13b in toluene-*d*8 at 298 K.

Molecular mechanic calculations[2]

Molecular mechanic calculations were performed using MacroModel version 9.8 (Schrödinger Inc.). All structures were minimized first and the minimized structures were then used as the starting molecular structures for all MacroModel conformational searches. The force field used was MMFFs as implemented in this software. The charges were defined by the force field library and no cut off were used for non-covalent interaction. H-bonds were fixed by constraining the distance between the phenolic hydroxyl and phosphine oxide functionalities to 2 ± 1 Å. A Polak-Ribiere Conjugate Gradient (PRCG) was used and each structure was subjected to 10000 iterations. The minima converged on a gradient with a threshold of 0.01. Conformational search was performed from previously minimized structures using 10000 steps. Only the structures in a 5 $kJ \cdot mol^{-1}$ windows from the global minimum were analysed.

X-ray structure of the AA 2-mer of backbone N8

Pure compound (3 mg) was dissolved in MeCN (1 mL) in an NMR tube, resulting in crystallization after 3 days at room temperature. Crystals suitable for X-ray crystallography were selected using an optical microscope and examined at 100 K on a Bruker SMART APEX-II CCD diffractometer operating with a Cu Kα sealed tube X-ray source. The structures were solved using SHELXL-97 and refined using WinGX V1.64.05.23.24. All non-hydrogen atoms were refined anisotropically. Hydrogen atoms were placed in idealised position.

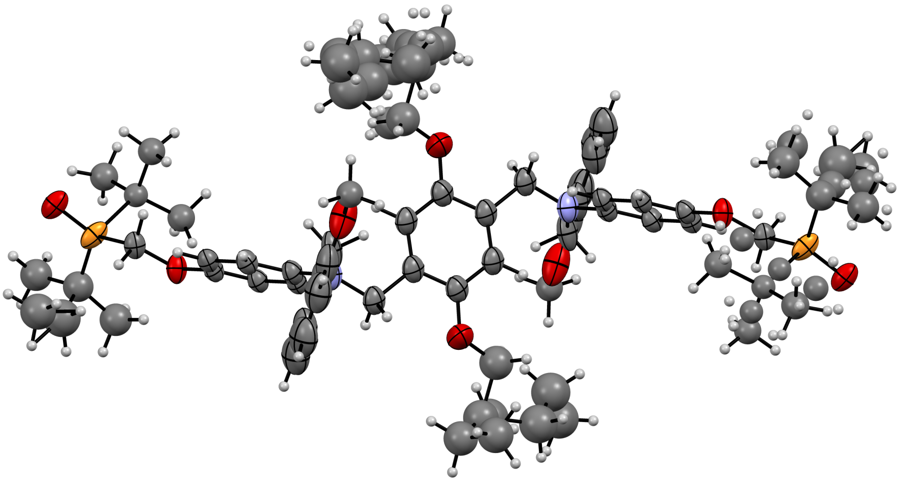

Figure S3. X-ray structure of derivative AA 2-mer (backbone N8) in ORTEP view (ellipsoids are drawn at 50% probability level).

**Formula**	$C_{70}H_{107}N_2O_8P_2$
**Temperature / K**	100
**Space Group**	$P2_1/c$
**Cell Lengths/ Å**	a 21.0973 (5) b 11.5790 (3) c 14.960 (4)
**Cell Angles/ °**	α 90 β 109.03 (2) γ 90
**Cell Volume/ Å^3**	3454.81
**Z**	2
**R factor**	15.4

References

1 C. Xue; F. T., *J. Org. Chem.*, **2003**, 68, 4417.

2 MacroModel, version 9.8, Schrödinger, LLC, New York, NY, 2014.
